# The Emotional Crying Behavior Dataset (ECBD): A comprehensive resource to study the multifaceted nature of emotional crying

**DOI:** 10.3758/s13428-025-02766-4

**Published:** 2025-09-10

**Authors:** Monika Wróbel, Janis Zickfeld, Paweł Ciesielski

**Affiliations:** 1 Institute of Psychology, University of Lodz, Lodz, Poland; 2 Department of Management, Aarhus University, Fuglesangs Allé 4, 8210 Aarhus V, Denmark; 3 Faculty of Psychology and Cognitive Sciences, Adam Mickiewicz University, Poznań, Poland

**Keywords:** Crying, Emotional tears, Dynamic stimuli, Emotional expression, Video dataset

## Abstract

Emotional crying is a complex and multifaceted expression that is frequently observed in humans. Its communicative effects have been recently studied in more detail. However, many studies focus on just one specific feature of emotional crying, most often emotional tears, neglecting the complex nature of the expression. Reports about crying episodes observe that tears most commonly occur in combination with other features such as facial expressions, vocalizations, gestures, and varied temporal dynamics. This shortcoming in research is mostly explained by a lack of adequately controlled stimuli depicting different crying features. Here, we provide a solution for this problem by introducing the Emotional Crying Behavior Dataset (ECBD), an openly available resource of 500 videos depicting 10 actors posing variations in tear intensity, facial expression intensity, vocalizations, gestures, and temporal dynamics, and the combination thereof. We present two studies (*N* = 2,729) providing evidence for the validity of the dataset. In addition, we developed a static supplementary resource (ECBD-S) with 70 pictures depicting variations in tears and facial expression intensity that was successfully validated across two studies (*N* = 601). Overall, our findings support the validity of this new stimulus set that closes a gap in the research on the interpersonal effects of emotional crying. The dataset is openly available for non-commercial research purposes at https://zenodo.org/records/15147817.

**Supplementary Information:**

The online version contains supplementary material available at 10.3758/s13428-025-02766-4.

## Introduction

Emotional crying constitutes a ubiquitous, uniquely human behavior that serves as a strong signal in social communication (Vingerhoets, 2013; Zickfeld & Wróbel, 2024). Especially emotional tears have been found to elicit increased helping and social support intentions in others (Gračanin et al., 2017; Zickfeld & Wróbel, 2024; Zickfeld et al., 2021). Given its potential relevance in social interactions, scholars have recently investigated the role of perceptions and evaluations of emotional crying across a range of situations, cultures, and outcomes (Gračanin et al., 2021; Hendriks & Vingerhoets, 2006; MacArthur & Shields, 2019; Van de Ven et al., 2017; Vingerhoets et al., 2016; Zickfeld et al., 2021). The majority of these studies have tested the signal effects of emotional crying by comparing static images of criers and non-criers, most commonly by manipulating visible tears (Krivan & Thomas, 2020; Zickfeld & Wróbel, 2024), which has been criticized for limited ecological validity (Barthelmäs et al., 2024). However, emotional crying is considered a complex and multifaceted expressive behavior that goes beyond shedding emotional tears (Barthelmäs et al., 2024; Murube, 2009; Vingerhoets, 2013). An emotional crying episode might consist of expressive features (e.g., facial expressions), vocalizations (e.g., sobbing, wailing), gestures or body movements (e.g., face touching), or a combination of two or several of those (Barthelmäs et al., 2024; Green et al., 2011). Similarly, the intensity of these features and the temporal dynamics might strongly impact how the crier and their behaviors are evaluated and perceived. The current reliance on static stimuli that only differ in one crying feature – emotional tears – might strongly impact the generalizability of findings (see Barthelmäs et al., 2024). To close this gap, we introduce a validated dataset including dynamic posed presentations of different crying features and their combinations – the Emotional Crying Behavior Dataset (ECBD). The dataset was constructed using simulated tears, that is, eye drops applied to the actors’ faces before the recording of each video. This technique is very common among professional actors, enabling them to produce natural-looking tears (Sack, 2024; Yekanians, 2019). However, its use in psychological studies is limited, as to the best of our knowledge, only two studies (both using a single female actor) successfully applied this method (Reed et al., 2015, 2019). Nevertheless, in contrast to relying on tears shed spontaneously (e.g., Bobowik et al., 2024; Küster et al., 2022a), which, as we elaborate below, are difficult to standardize, using simulated tears enabled us to create a rich, fully balanced stimulus set.

## Emotional crying

Emotional crying is loosely defined as crying in response to an emotional event or part of an emotional episode (Vingerhoets, 2013).^1^ In addition, most researchers agree that emotional crying can consist of different *features* (Barthelmäs et al., 2022). Such features include shedding of emotional tears, specific facial expressions, vocalizations such as sobbing, screaming, or whining (Green et al., 2011), body movements and gestures such as touching or hiding the face, or eye redness (due to prolonged irritation). While this list is not exhaustive, it is unclear which of these features are necessary or sufficient for an expression to be characterized as emotional crying. Without a doubt, a sad facial expression is not necessarily interpreted or perceived as emotional crying. Some scholars have argued that emotional tears are the most dominant feature of emotional crying (Gračanin et al., 2018), but there are few systematic or empirically grounded efforts to provide a comprehensive definition of emotional crying and identify which of its features are primary or secondary. Similarly, unique features are often synonymously referred to as *emotional crying*. For instance, studies use this term when focusing on emotional tears only (e.g., Zickfeld et al., 2021) or manipulating other features except tears (e.g., Fischer et al., 2013).

Considering the complexity of emotional expressions and their temporal dynamics (Cowen & Keltner, 2020; Jack et al., 2014), it seems vital to systematically assess the interplay of different features of emotional crying and go beyond single features. Retrospective self-reports suggest that several features of emotional crying are present when thinking about episodes of emotional crying (Barthelmäs et al., 2024). The same study only observed one single episode (out of 509) in which tears dominated, and other features were mostly absent. At the same time, the importance of focusing on several features is emphasized by vastly contrasting findings of studies focused on single features only. For instance, research on the perception of visual crying (i.e., emotional tears) has mainly identified positive reactions, such as intentions to support the crier (Zickfeld et al., 2021), whereas research on vocalizations of crying showed that prolonged acoustic crying might trigger more negative reactions such as frustration or anger (Barr et al., 2014). To our knowledge, there exists no project systematically testing a combination of the different features of emotional crying and evaluating their importance. We think that one main reason for this gap is the availability of highly controlled stimuli expressing these (combinations of) features. To elucidate this problem, we will turn to reviewing common practices and resources in studying the expressive signals of emotional crying.

## Stimuli and datasets to study emotional crying

While there exists a myriad of approaches, responses to emotional crying have been mostly studied using three different experimental methods, including perceptions of a) descriptive vignettes, b) static pictures, or c) dynamic videos. As an exception, to our knowledge, one study has investigated the presence of emotional tears in an actual interaction (Hill & Martin, 1997), and one recent paper investigated retrospective reports of perceptions of actual emotional crying episodes (Barthelmäs et al., 2024). In addition, there are several stimulus sets that were mostly created ad-hoc, with only one being systematically validated (Küster et al., 2022a, 2022b). An overview of the most common stimuli and datasets employed in the study of emotional crying is provided in Table 1. Note that there exist additional studies using stimuli depicting emotional crying, but they are not listed as they mainly focus on one or two targets only (e.g., Aragón & Clark, 2018; Bobowik et al., 2023, 2024; Reed et al., 2015, 2019).

**Table 1:**
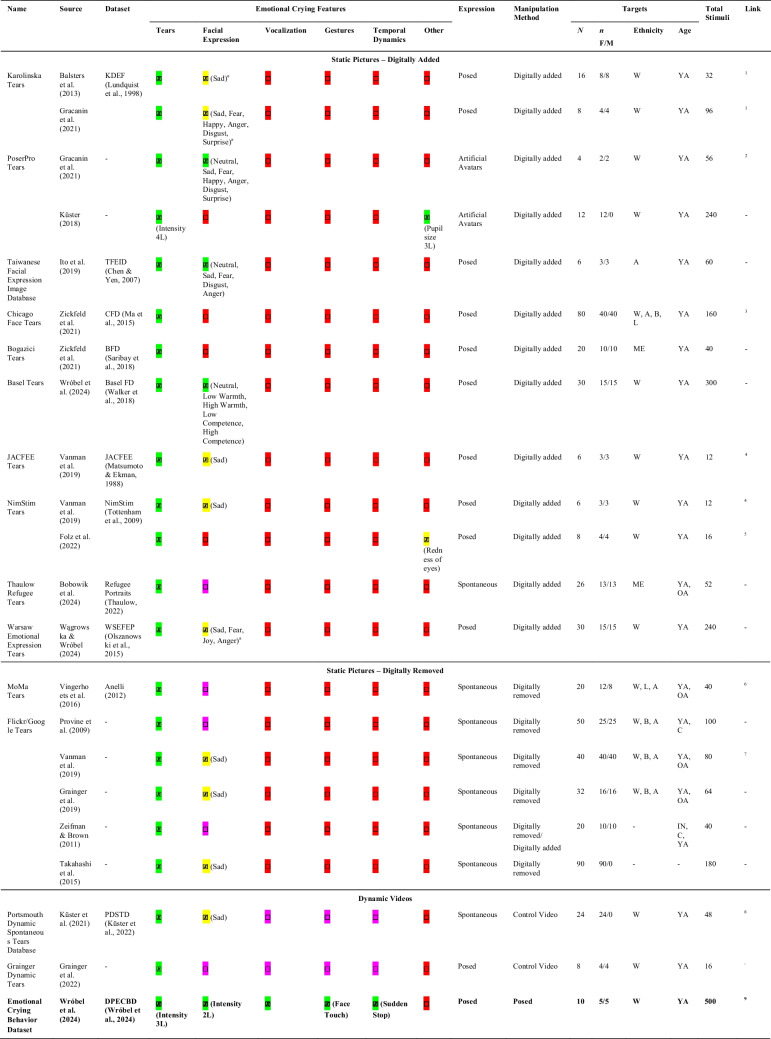
Overview of common resources and datasets to investigate emotional crying perceptions

= Manipulated full factorial;  = Manipulated but not full factorial (e.g., no control condition with the absence of this factor);  = Not manipulated; likely to vary on this factor; **=** Not manipulated; *n* F/M = Number of female/male targets; Ethnicity: W = White/Caucasian; B = Black; A = Asian, ME = Middle Eastern; Age: IN = Infant, C = Children, YA = Younger Adults, OA = Older Adults; 2L = 2 levels, 3L = 3 levels, 4L = 4 levels. ^a^Neutral control not included in the study but available as part of the KDEF and WSEFEP. ^1^ https://www.kdef.se/ (only original stimuli without tears); ^2^ https://osf.io/dx8cg/; ^3^ https://www.chicagofaces.org/resources/; ^4^ https://osf.io/s4q8d/; ^5^https://dataverse.nl/file.xhtml?fileId=199518&version=2.0;^6^https://osf.io/bmn2v;^7^https://osf.io/49xzv/;^8^https://osf.io/uyjeg/?view_only=24474ec8d75949ccb9a8243651db0abf;^9^https://zenodo.org/records/15147817

### Descriptive Vignettes

One approach to studying responses to emotional crying has been using descriptive vignettes (Hendriks et al., 2008; Wong et al., 2011; Gallegos et al., 2019; Yasuhara & Takehara, 2023). In this scenario, participants receive a description of someone crying. The main advantage is that different crying features can be easily manipulated, as no graphical stimuli need to be created or edited. Yet, most studies using this approach focused only on one or two visual features of crying. Importantly, visual, acoustical, and movement features of emotional crying are more difficult to manipulate using vignettes. Participants can imagine someone shedding tears, but this is likely not as strong as observing an actual person shedding tears or crying, which is probably one main reason why researchers have preferred graphical stimuli.

### Static Pictures

The majority of recent experimental studies have tested responses to emotional crying by showing participants variants of a static picture of the same target with and without crying features (for a review, see Zickfeld & Wróbel, 2024). There are two main approaches by either taking *control* pictures and digitally or physically *adding* features (Balsters et al., 2013; Bobowik et al., 2023; Folz et al., 2022; Gračanin et al., 2021; Ito & Ong, 2023; Reed et al., 2019; Zickfeld et al., 2021) or by taking *crying* pictures and digitally *removing* features (Picó et al., 2020; Provine et al., 2009; Van de Ven et al., 2017; Grainger et al., 2019, 2022; Vingerhoets et al., 2016; Zeifman & Brown, 2011; Zickfeld et al., 2018; Zickfeld & Schubert, 2018). Most of these studies manipulated visual features, including visual tears or facial expressions.

Studies using stimuli with added features typically take two routes. In one option, a *neutral* posed expression of a target is used as the baseline to which tears are added (Küster, 2015; Reed et al., 2015; Zickfeld et al., 2021). Such tears can be added digitally using photo editing software (Küster, 2015) or physically by using eye drops and then taking a picture of the target (Reed et al., 2015). These stimuli have the advantage that they can fully isolate the effect of emotional tears, but the manipulated pictures typically lack other crying features, which might strongly impact their ecological validity and how authentic these are perceived. This might be further limited by whether the added tears look realistic (e.g., naturally reflect light or flow in a direction resembling a natural tear flow, matching facial anatomy). In addition, few studies systematically control for the intensity of tears, redness of the eyes, or pupil size (Folz et al., 2022; Küster, 2018), but such factors are typically ignored. Common datasets within this approach include modified images of the Chicago Face Database (Ma et al., 2015), Bogazici Face Database (Saribay et al., 2018), or Karolinska Directed Emotional Faces (Lundqvist et al., 1998) (see Table 1). In another option, researchers take a target posing an emotional facial expression as the baseline to which tears are then digitally added (Balsters et al., 2013; Bobowik et al., 2023; Ito et al., 2019). Such facial expressions are typically characterized as *sadness*, but some studies also focus on other expressions associated with emotions such as *happiness*, *anger*, or *disgust* (Gračanin et al., 2021; Ito & Ong, 2023; Ito et al., 2019). One main advantage is that they provide more ecologically valid stimuli that also show a specific facial expression feature instead of a *neutral* expression that is uncommon for emotional crying (Barthelmäs et al., 2024). At the same time, it gets more difficult to isolate and separate the effects of tears and facial expression features of emotional crying if no *neutral* control target is employed. Stimuli collections within this approach include modified images of the Karolinska Directed Emotional Faces (Lundqvist et al., 1998), Taiwanese Facial Expression Image Database (Chen & Yen, 2007), or NimStim Set of Facial Expressions (Tottenham et al., 2009) (Table 1).

Studies using stimuli with removed features take a different approach. Here, researchers collect a set of targets showing genuine emotional crying features and then remove such features digitally using photo editing software (Grainger et al., 2019; Provine et al., 2009; Vingerhoets et al., 2016; Zeifman & Brown, 2011). These approaches differ in whether stimuli are sourced via the internet (Grainger et al., 2019; Provine et al., 2009; Zeifman & Brown, 2011) or taken by professional photographers (Vingerhoets et al., 2016). Most commonly, these approaches only remove tears and do not alter facial expressions or body movements. Their main advantage is the fact that the target shows a genuine and spontaneous emotional expression (including realistically looking tears), which aligns with a recent increasing call for spontaneous instead of posed expression stimuli, given their higher ecological validity (Krivan & Thomas, 2020; Küster et al., 2022a, 2022b). At the same time, the baseline does not typically show a full absence of crying features, thereby reducing experimental control. An overview of different resources using this approach is provided in Table 1.

To summarize, static pictures are commonly used based on their simplicity and the possibility of manipulating (visual) crying features. Approaches adding tears (posed expression) typically provide more experimental control, while approaches removing tears (spontaneous expression) typically provide more ecological validity. Given the static nature of pictures, the manipulation of movements, vocalizations, or temporal dynamics is not possible for any of these approaches, representing one major disadvantage in the use of static images. As presented in Table 1, the reviewed resources differ substantially in the number of included images and how these vary on demographic variables, with the majority including only a handful of targets limited to White/Caucasian younger adults. Similarly, only a small number of reviewed resources can be openly accessed.

### Dynamic videos

Few studies have employed video stimuli showing dynamics and temporal changes in emotional crying (Aragón & Clark, 2018; Bobowik et al., 2024; Grainger et al., 2022; Küster et al., 2022a, 2022b; Reed et al., 2015, 2019). These studies mainly differ in whether they include posed (Reed et al., 2015, 2019) or spontaneous natural expressions (Bobowik et al., 2024; Grainger et al., 2022; Küster et al., 2022a, 2022b). Similar to static images, studies either focus on specifically manipulating a few crying features, most prominently emotional tears, by using eye drops (Reed et al., 2015, 2019), instructing targets to think of emotional events (Grainger et al., 2022), or taking footage of actual criers and then trying to match control videos of the same person without crying features (Bobowik et al., 2024; Küster et al., 2022a, 2022b). These approaches again have their advantages, with the first one more experimentally controlling for the presence or absence of different crying features and the second and third ones providing more ecologically valid depictions.

Videos depicting posed vs. spontaneous crying, similar to static images showing tears added vs. removed, might also differ in how tears *per se* look. Using standardized settings (e.g., good lighting conditions, minimal movement, professional actors) and methods (e.g., simulating tears via eye drops) may result in creating videos with tears that are very well visible and unambiguous, while footage of people crying spontaneously (especially when these people move) may show tears less explicitly. Notably, this difference in explicitness is well-recognized in studies on posed vs. spontaneous emotional expressions in general (Cong et al., 2025; Krumhuber et al., 2021), but its implications for the overall perception of tears and crying (e.g., their intensity or authenticity) remain unknown.

Most studies use one or two targets and create video stimuli for a specific purpose. An exception is the Portsmouth Dynamic Spontaneous Tears Database (PDSTD; Küster et al., 2022a, 2022b), which includes dynamic stimuli depicting 24 female targets with or without tears that have been explicitly validated (Table 1). While the PDSTD features spontaneous and dynamic reactions of emotional crying, it has the main drawback that it does not explicitly control for other emotional crying features than tears, and only includes female targets. For instance, some targets in the dataset show intense facial expressions, body movements, or vocalizations that the dataset does not control for. Therefore, it is difficult to systematically evaluate the unique and combined effects of different crying features using this dataset. In addition, the dataset and other video stimuli do not explicitly differentiate the intensity levels of the features (e.g., some targets might shed more intense and others more subtle tears). Such features and their levels could be potentially coded ad-hoc, but it is possible that not enough variation exists due to the sourcing of the stimuli.

Overall, dynamic stimuli provide more fine-grained and detailed information about crying episodes than static stimuli. Still, they are used less frequently, possibly due to only a few datasets being available and presenting dynamic stimuli being more costly in online studies. In addition, just as static images, the majority of dynamic stimuli focus on manipulating one aspect of emotional crying – visual tears.

## Introducing the Emotional Crying Behavior Dataset (ECBD)

Emotional crying is a multifaceted behavior, and to be able to study it validly, adequate stimulus material is necessary. The currently available stimuli (Table 1) capture broad features and variations in emotional crying but mainly fall short on four main aspects. First, they mostly focus on one specific feature of emotional crying, namely emotional tears, neglecting other features that are not always or never experimentally manipulated. For instance, while tear intensity has been identified as an important variable in influencing perceptions of crying (MacArthur & Shields, 2019; Zickfeld & Wróbel, 2024), few of the available stimuli manipulate or control for this aspect (see Küster, 2018, for an exception). Similarly, none of the resources reviewed in Table 1 controls for vocalizations such as sobbing, which have been considered important features of emotional crying (Barthelmäs et al., 2024). Second, as the available datasets focus on a particular feature of emotional crying, they often fail to test combinations of features. For example, is someone shedding tears combined with an intense facial expression and sobbing perceived differently than someone shedding subtle tears with a natural facial expression and no vocalizations? To our knowledge, no set of stimuli can experimentally test such questions. Third, as emotional crying is a complex expression including temporal dynamics, dynamic instead of static stimuli are needed. While some dynamic stimuli exist, most studies still focus on static pictures, most likely due to availability and ease-of-use constraints. Fourth, while at least one exception exists (Küster et al., 2022a, 2022b), few of the available stimuli are explicitly validated and mostly in an ad hoc manner. In addition, a limited number is openly available. Some resources might be available upon request to the authors, but it is likely that some stimuli cannot be shared due to using proprietary content from a third-party dataset or due to missing consent by targets.

To address these four shortcomings, we introduce the Emotional Crying Behavior Dataset (ECBD), to our knowledge, the most comprehensive dataset of video stimuli depicting various crying features and their combinations. The ECBD includes 10 professional actors (women and men), depicting a combination of five different crying features, including facial expression intensity (natural vs. exaggerated), tear intensity (no tears vs. subtle tears vs. intense tears), vocalizations (quiet vs. loud), body movements (no face touching vs. face touching), and the temporal dynamic of crying (sudden vs. gradual stop of crying). All features are combined in a factorial design, resulting in 50 unique videos per actor (including one baseline video with all features being absent and one baseline video with face touching only) and a final set of 500 videos. The resource is openly available for non-commercial research purposes via Zenodo (https://zenodo.org/records/15147817).^2^

All videos were validated across two studies, including a total of *N* = 2,729 participants. In Study 1 (*n* = 1,100), we tested whether the manipulated features are perceived as intended (for instance, whether the targets showing exaggerated facial expressions are perceived as having more exaggerated facial expressions than those showing subtle facial expressions). In Study 2 (*n* = 1,629), we measured participants’ inferences about the targets, which enabled us to test the so-called *tearing effect*, a well-established tendency to perceive crying individuals as sadder (and more helpless) compared to a control (Küster et al., 2022a, 2022b; Ong & Ito, 2022; Provine et al., 2009; Zickfeld et al., 2021). In addition, we asked participants to rate the overall intensity of the target’s expression (in contrast to Study 1, where we asked specifically about facial expression intensity). To supplement the dataset for contexts in which presenting video stimuli is unfeasible (e.g., slow internet connection, pen-paper questionnaires), we also provide a validated set of 70 pictures (ECBD-S) based on the video stimuli depicting targets across the features of facial expression and tear intensity. This dataset was again validated across two studies, including a total of *N* = 601 participants, in order to evaluate ratings of manipulated features and test whether these features replicate a tearing effect.

We will first detail the video stimulus development of the ECBD and then present the two pre-registered studies to validate the dataset. Afterward, we will focus on the additional resources, including static images based on the original videos, their development, and two studies validating them.

## Open science & ethical considerations

All four studies reported in the manuscript were pre-registered (https://osf.io/g63ny/registrations). All materials, data, and syntaxes for these studies are available at https://osf.io/g63ny/. The dataset, including all dynamic and static stimuli, is openly available for non-commercial research purposes upon request at https://zenodo.org/records/15147817. All participants in the current studies provided informed consent, and the studies were approved by the ethical review board of the University of Lodz. Actors included in the dataset signed release forms stating that their depictions can be used for non-commercial research purposes.

## Video dataset validation

### Video stimulus development

Based on previous definitions and findings (e.g., Barthelmäs et al., 2022, 2024; Zickfeld & Wróbel, 2024) and two preliminary studies conducted for a different project (see Supplementary Material Section 1), we identified five main and common features of emotional crying that should be depicted in the current stimuli. First, we focused on emotional tears, which have been considered the most common and primary expression of emotional crying (e.g., Gracanin et al., (Gračanin et al., 2018)). Based on previous studies (e.g., Küster, 2018) and the results of Preliminary Study 2, where participants listed the intense flow of tears as an important crying feature (Supplementary Material Section 1.2), we focused on three different intensities in tears, namely *no tears*, *subtle tears*, and *intense tears,* operationalizing them as “no tears visible,” “1-2 tears visible,” and “several tears visible”, respectively. Second, we focused on facial expressions that are typically associated with emotional crying (Barthelmäs et al., 2024; preliminary studies - Supplementary Material Section 1). As different facial expressions and emotions can be associated with emotional crying (Vingerhoets, 2013), we mainly instructed actors to show expressions of crying but did not specify a particular emotion. Most actors depicted the typical *sadness* expression^3^ which has been most strongly associated with emotional crying (Ito et al., 2019, 2023). We focused on depicting two different intensities of the expression: a more *natural* expression and an *exaggerated* one. Third, vocalizations are commonly reported for (intense) emotional crying (Barthelmäs et al., 2024; Cordaro et al., 2016). Therefore, we focused on depictions with and without vocalizations. Due to the complexity of different vocalizations, we only included their absence or presence. Fourth, gestures or body movements are commonly reported for emotional crying (Barthelmäs et al., 2024; current pilot studies in Supplementary Material Section 1). In our preliminary studies, face-touching behavior was mentioned most frequently for this feature, which is why we focused on depictions of face-touching. Fifth, the temporal dynamics of crying behavior can be important. The preliminary studies identified frequent mentions of criers suddenly stopping their crying behavior, especially when they were perceived as manipulative. For this reason, we included the fact of whether the crying behavior suddenly stopped or gradually faded out. Crossing these five factors, we obtained a total combination of 48 possibilities (e.g., no tears – natural expression – no vocalization – no face touching – gradual stop; subtle tears – exaggerated expression – vocalization – face touching – sudden stop, etc.). We also added two control conditions, one with all factors absent and one with all factors absent and face touching present. Thus, we focused on 50 combinations of features per actor (Supplementary Table 1).

**Fig. 1:**
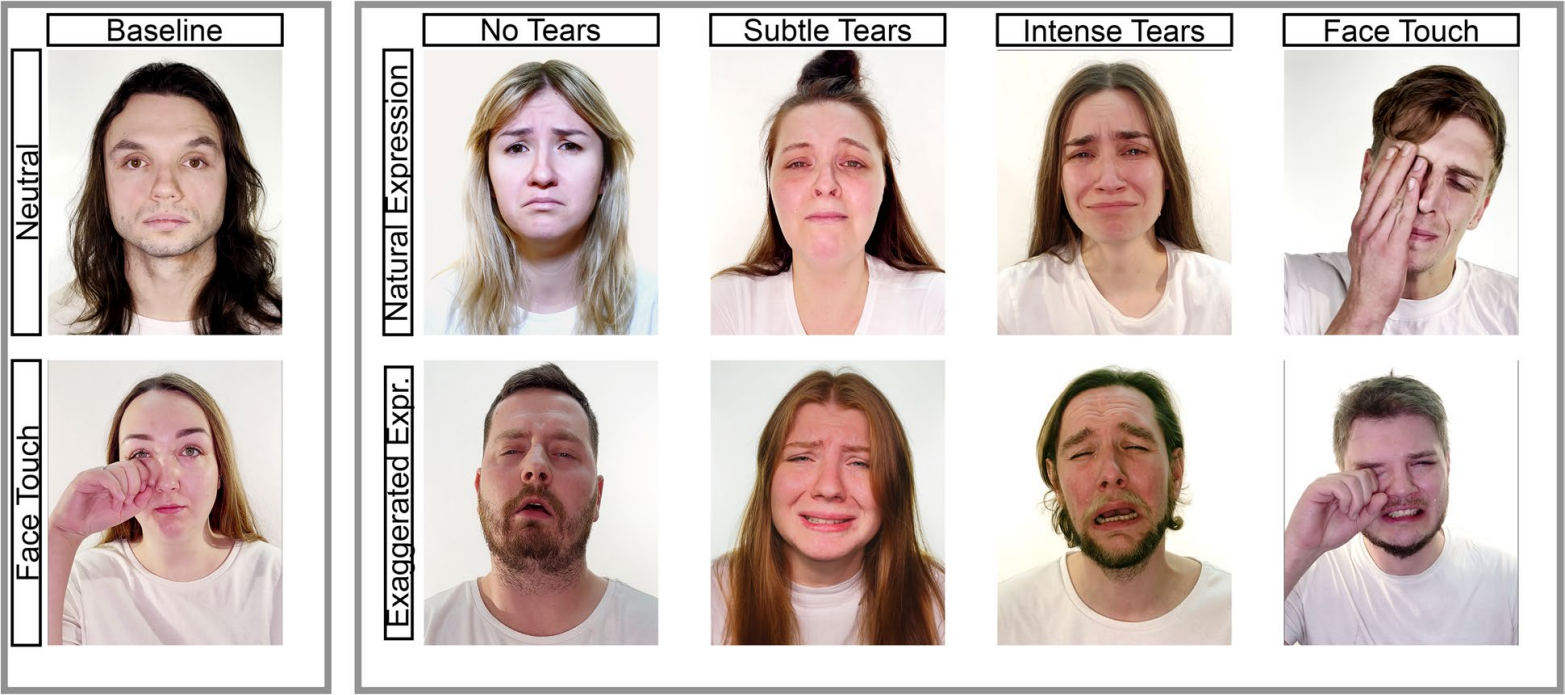
Example stills from the ECBD showing the baseline pictures (left) and crying pictures (right) with facial expression intensity, tear intensity, and face touching. All pictures except for the face-touching pictures are taken from the final validated static dataset (ECBD-S)

Videos were produced by a film production agency (“315 Studio”, https://315studio.pl) that was provided with explicit instructions on the 50 combinations (Supplementary Table 1). The agency recruited 10 professional Polish actors (5 women, 5 men) who signed a consent form that their videos could be used and distributed for non-commercial scientific research purposes. Each actor was provided with instructions to show behaviors based on the 50 possible combinations. For instance, in the combination of subtle tears, natural expression, face touching, loud crying, and gradual stopping, actors were instructed to perform all these behaviors at the same time. Videos were recorded using a Nikon D7500 at a frame rate of 60 fps. Actors were recorded from the shoulders upwards, looking directly at the camera. All the actors wore white T-shirts and were filmed against a white backdrop. T-shirts were replaced if they got wet from crying. Tears were simulated by using water droplets in different intensities applied to the eye to ensure their visibility. The amount of tears needed (40 videos with some form of tears per actor, including possible repetitions) made it impossible to elicit tears via emotional sources (e.g., watching a sad movie) or employing menthol tear sticks. Sudden stopping was performed by the actor by switching all expressions to neutral at the end of the video. Gradual stopping was achieved by fading the video to black. For each combination, several videos were recorded at about 10 s length (*M* = 11.04, *SD* = 1.73, min = 4.71, max = 15.93). After receiving videos from the agency, all authors screened videos based on the presence or absence of the different features and selected the videos that met the intended criteria or provided feedback to the agency to re-record the videos that failed to meet the criteria. This procedure typically took three rounds back and forth until all videos were obtained (see Supplementary Section 2 for more detailed information). Examples of the combinations of different features are provided in Figure 1.

### Video validation: Study 1

We performed a first validation with ratings assessing the five manipulated features to evaluate how well they were depicted in each video.

#### Method

##### Participants 

We registered to obtain at least 20 ratings per video. Since the total pool consisted of 500 videos and each participant rated 10 videos in this study, 1,000 participants were needed. This enabled us to reduce fatigue effects and make sure that each participant was not exposed to the same actor more than once. To account for possible exclusions and random distribution, we registered recruiting 1,100 participants.

A total of 1,129 UK-based participants were sampled via Prolific.com for a 10-minute study with £1.10 as compensation. As registered, we excluded *n* = 28 participants because they failed at least one of the two attention checks and *k* = 342 observations because they spent less than 15 s on the video and rating page (this excluded all observations of *n* = 1 participant). The final sample size included 1,100 participants (542 males, 545 females, 9 non-binary, 4 other) ranging from 18 to 79 years of age (*M* = 43.4, *SD* = 14.2). Due to random distribution, on average 21.34 (*SD* = 4.59) ratings were obtained per video (9 to 35 ratings per video; 30 videos with less than 15 ratings). In total, the dataset included *k* = 10,668 ratings.

##### Design & procedure

 We employed a 10 (actor) x 3 (tears: no tears vs. subtle tears vs. intense tears) x 2 (facial expression: natural vs. exaggerated) x 2 (touching: no face touching vs. face touching) x 2 (vocalizations: quiet vs. loud) x 2 (stopping: sudden vs. gradual) mixed design. This resulted in 480 possible combinations, and we also included two control videos per actor, thereby having 500 possible combinations and videos in total.

After providing informed consent, participants were presented with instructions. First, they were asked to watch and listen to a video to adjust their audio volume for the study. In addition, they were shown a video comprehension check to determine whether they watched the video with sound. The video depicted a bird, but the audio included a cat meowing. Thus, participants were only able to correctly answer the comprehension question if they both watched and listened to the video. Participants failing the video comprehension check the first time (*n* = 72) were shown the video and video comprehension check once more. If they failed repeatedly, the survey was automatically terminated (*n* = 3). After the video comprehension check, participants were shown 10 videos (development described above), one randomly chosen video per actor, in random order. After the 5th and 10th videos, participants were presented with an attention check (“Please select ‘3’ on the scale”). Finally, participants completed information regarding their gender, age, and nationality.

##### Measures

 For each video, participants completed the same measures asking about the intensity of tears (“This person… is not shedding tears at all” (1)/“shedding a lot of tears” (7)), facial expression (“This person… has a very subtle facial expression” (1)/“has an exaggerated facial expression” (7)), face touching (“This person… is not covering/touching their face” (1)/“is covering/touching their face a lot” (7)), vocalizations (“This person… is crying very quietly” (1)/is crying very loudly” (7)), and sudden stopping (“This person… does not suddenly change their expression to neutral at the end of the video” (1)/“suddenly changes their expression to neutral at the end of the video” (7)).

##### Statistical analysis

All analyses for this and the other studies reported in this manuscript were performed in *R* v.4.4.1 (R Core Team, 2024). For data wrangling, we employed *tidyverse* (v.2.0.0; Wickham et al., 2019) and *janitor* (v.2.2.0; Firke, 2023). For graphical illustrations we used *concaveman* (v.1.1.0; Gombin et al., 2020), *ggforce* (v.0.4.2; Pedersen, 2024), *ggradar* (v.0.2; Bion, 2024), *ggpubr* (v.0.6.0; Kassambara, 2023), *gghalves* (v.0.1.4; Tiedemann, 2022), *papaja* (v.0.1.2; Aust & Barth, 2023), *NbClust* (v.3.0.1; Charrad et al., 2014), *see* (v.0.9.0; Lüdecke et al., (Lüdecke et al., 2021)), and *viridis* (v.0.6.5; Garnier et al., 2024). For multilevel models, we employed *lme4* (v.1.1.35.5; Bates et al., 2015), *sjPlot* (v.2.8.16; Lüdecke, 2024), *performance* (v.0.12.4; Lüdecke, (Lüdecke et al., 2021)), *robustlmm* (v.3.3.1; Koller, 2016), and *emmeans* (v.1.10.3; Lenth, 2024). For inter-rater reliability, we used *psych* (v.2.4.6.26; Revelle, 2024), *irr* (v.0.84.1; Gamer & Lemon, 2019), and *irrCAC* (v.1.3; Gwet, 2023).

##### Multilevel models 

For all multilevel models in the manuscript, we employed the *lme4* package (Bates et al., 2015). We fitted models with restricted maximum likelihood (REML) estimation. In case of non-convergence, we re-fitted models using the Nelder-Mead optimization (note that this step was not preregistered). As registered, we fitted models with random effects for participants and videos nested in actors, but did not include random slopes due to model complexity. For Study 2, we also nested participants in sample. We checked model assumptions using the *performance* package (Lüdecke et al., 2021). Many models violated assumptions of homoscedasticity and some normality of residuals. Although multilevel models have been considered rather robust to assumption violations (Schielzeth et al., 2020), we refitted models using robust estimation using the *robustlmm* package (Koller, 2016). We provide results of the registered models in the manuscript and additional information on robust models in the Supplementary Materials. Note that robust model estimation was not preregistered.

#### Results

##### Norming data

Norming data for each video can be found at https://osf.io/tegw2/. This includes average ratings of tear intensity, facial expression, face touching, vocalizations, and sudden stopping for each video.

##### Video validation

**Fig. 2:**
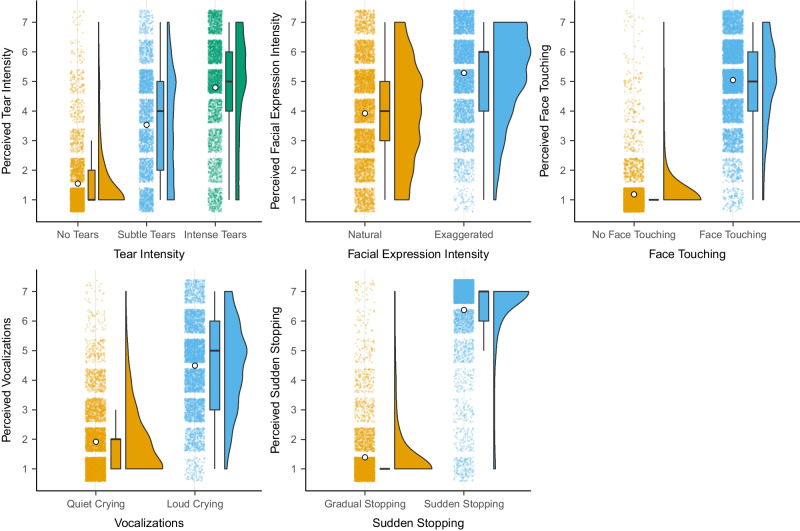
Overview of perception ratings across all participants (*n* = 10,668) for the five different variables: perceived tear intensity, perceived facial expression intensity, perceived face touching, perceived vocalizations, and perceived sudden stopping. Raincloud plots show **a**) individual data points, **b**) boxplots with median (thick horizontal line), lower, and upper quartiles, and **c**) data distributions. White dots indicate descriptive means

**Fig. 3:**
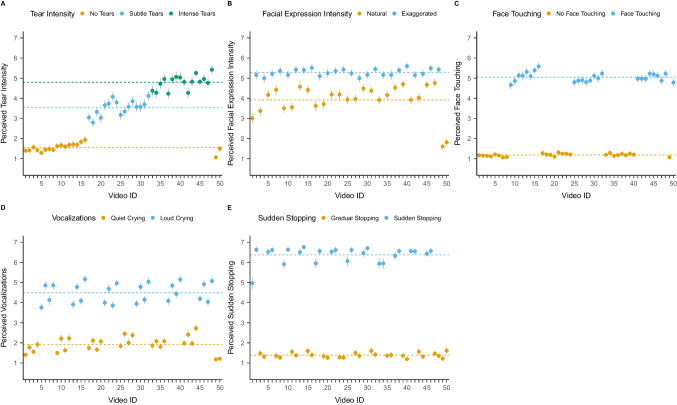
Overview of perception ratings of tear intensity (**A**), facial expression intensity (**B**), face touching (**C**), vocalizations (**D**), and sudden stopping (**E**). Dots represent means and lines 95% CIs. Horizontal dashed lines represent respective subgroup means

To check the validity of each video, we first conducted a multilevel model with one of the five measures (tear intensity, facial expression, face touching, vocalizations, or sudden stopping) as the outcome variable and the respective factor coding for the absence/presence of that manipulation as a predictor. We also added participants as random effects and videos nested in actors.^4^ An overview of ratings is presented in Figure 2, and detailed models are provided in Supplementary Section 3.1 (Supplementary Tables 2-6). For tear intensity, we observed that videos that should show *subtle tears* were rated as higher in tear intensity (*M* = 3.55, *SE* = 0.09) than videos that should show no tears (*d* = 0.97 [0.90, 1.04], *p* < 0.001). Similarly, videos that should show *intense tears* were rated as higher in tear intensity (*M* = 4.80, *SE* = 0.09) than no tears videos (*d* = 1.58 [1.51, 1.65], *p* < 0.001) and *subtle tears* videos (*d* = 0.60 [0.53, 0.68], *p* < 0.001). We also observed that videos including actors showing an exaggerated facial expression were rated higher in facial expression intensity (*M* = 5.29, *SE* = 0.14) than actors instructed to show a natural facial expression (*M* = 3.90, *SE* = 0.14; *d* = 0.80 [0.72, 0.87], *p* < 0.001). Similarly, videos of actors instructed to touch their faces were perceived as including more face touching (*M* = 5.05, *SE* = 0.06) compared to videos that should show no face touching (*M* = 1.18, *SE* = 0.06; *d* = 1.73 [1.70, 1.76], *p* < 0.001). Videos that should include crying vocalizations were rated as louder (*M* = 4.53, *SE* = 0.09) compared to videos that should include no vocalizations (*M* = 1.88, *SE* = 0.09; *d* = 1.43 [1.37, 1.49], *p* < 0.001). Finally, videos that should include a sudden stopping of the crying expression were more likely to be perceived as such (*M* = 6.39, *SE* = 0.03) compared to videos that should not show a sudden stop (*M* = 1.39, *SE* = 0.03; *d* = 1.80 [1.77, 1.83], *p* < 0.001). Random effects for all models suggested variation across participants and videos. We observed smaller variations across actors. An overview of ratings for the individual videos across all ten actors is provided in Figure 3.

Further, we computed a dichotomous variable for each perception rating, indicating the absence (below the mean) or presence of the variable (above the mean). For tear intensity, we coded subtle tears as matching for a rating between 2 and 4 and intense tears for a rating between 4 and 7 (this was not preregistered). We observed that perceptions of sudden stopping (or gradual stopping) matched on average in 93.85% of ratings (*SD* = 24.03), face touching (or no face touching) matched on average in 91.35% (*SD* = 28.12), and loud crying (or quiet crying) matched on average in 81.96% cases (*SD* = 38.45). We observed lower matching for facial expression intensity and tear intensity. First, facial expression intensity matched on average in 66.52% of ratings (*SD* = 47.19). Tear intensity on average for 61.16% of ratings (*SD* = 48.74). This is not surprising as rating the absence of a behavior (e.g., face touching absent or present) is easier than rating the intensity of a behavior (e.g., tears subtle or intense). A detailed overview of matching per video and actor is provided in Supplementary Figure 6.

Finally, we investigated inter-rater reliability and agreement across measures and videos. A detailed overview is provided in Supplementary Section 3.3. First, we tested the consistency of each of the five perception ratings and observed that variation was mainly attributed to differences between and not within videos. This was again somewhat smaller for ratings of tear intensity and facial expression intensity. Second, we tested the inter-rater reliability across the five perception ratings for each video separately by computing intraclass correlation coefficients (ICC) and Gwet’s (2023) agreement coefficient (AC; Supplementary Table 7 and Supplementary Figure 7). Inter-rater reliability was moderate to high for the large majority of videos, and we only observed poor reliability for a small portion (22 out of 500, see Supplementary Table 7 and Supplementary Figure 8). These analyses were not pre-registered.

We also performed exploratory hierarchical clustering of all videos, which can be found in Supplementary Section 3.4 (Supplementary Figure 9).

## Video validation: Study 2

We performed a second validation of the videos with ratings of the observers’ inferences, emotions, and behavior in response to the videos. These ratings were based on previous studies (for a review, see Zickfeld & Wróbel, 2024). The main purpose of Study 2 was for a different project, which is why some measures or sample size determination are not related to the current validation.

### Method

#### Participants 

We registered to recruit 400 participants per sample and a total of 1,600 participants (*k* = 6,400 observations). This was based on sample size estimation for random forest models. Based on this number, around 12 ratings were expected per individual video. However, we were mainly interested in ratings across videos, which is why this was deemed adequate.

We recruited a total of 1,692 participants via Prolific.com, sampling participants located in Canada (*n* = 410), South Africa (*n* = 417), Poland (*n* = 409), and Scandinavia (*n* = 456). Note that we originally registered to recruit the fourth sample from participants located in Sweden. However, after recruiting around half of the intended sample, we noticed that we were not able to recruit more people, which is why we also opened recruitment to people located in Denmark and Norway. We employed the same exclusion criteria as in Study 1. A total of *n* = 14 participants failed the comprehension video check twice (see below), *n* = 1 failed the attention check, and *k* = 271 observations were excluded because participants spent less than the full length of the video on the page (this included all four videos from 4 additional participants).^5^ In addition, *n* = 58 participants were excluded because they provided no data (including those who failed the comprehension checks twice). One of these participants was excluded because the system did not record the displayed video, and the response could not be used further (see Supplementary Table 8 for detailed exclusion criteria).

The final sample included a total of *N* = 1,629 participants and *k* = 6,261 observations (776 male, 819 female, 27 non-binary, 4 other, 3 missing) ranging from 18 to 73 years of age (*M* = 30.7, *SD* = 9.5). A detailed overview of the separate samples per location is provided in Supplementary Table 8.

#### Design & procedure

 We employed the same design as in Study 1, including a total of 500 videos.

After providing informed consent, participants were presented with instructions. First, they were shown the same “bird/cat” video and video comprehension check as in Study 1. Participants failing the video comprehension check the first time (*n* = 112) were shown the check again. If they failed twice, the survey was automatically terminated (*n* = 14). After the video comprehension check, participants were shown four videos randomly chosen from the total pool, but always just one video per actor. After the 4th video, participants were presented with an attention check (“Please select ‘3’ on the scale”). Finally, participants completed information regarding their gender, age, and nationality.

#### Measures

After each video, participants completed the same 11 items in a fixed order. All items were rated on a 7-point scale from *not at all* (1) to *very much* (7), and most of them were employed for the purposes of a different project.

#### Expression related 

Participants completed four items regarding the specific expression, focusing on the authenticity (“How authentic do you think the expression of this person is?”), intensity (“How intense do you think the expression of this person is?”), appropriateness (“How appropriate do you think the expression of this person is?”), sadness (“How sad do you think the expression of this person is?”) of the expression.

#### Inferences

In addition, participants rated how warm (“How warm does this person appear to you?”), helpless (“How helpless does this person appear to you?”), manipulative (“How manipulative does this person appear to you?”), and honest (“How honest does this person appear to you?”) they perceived the person.

#### Felt Emotions

Participants also rated how compassionate (“When seeing this person, I feel compassionate.”) and distressed (“When seeing this person, I feel upset”) they felt when seeing the picture.

#### **Social Support Intentions**

Finally, participants rated how much support they would offer to the person (“I would offer support to this person”).

### Results

#### Norming data

Norming data for each video across samples can be found at https://osf.io/tegw2/. This includes average ratings of all measured variables.

#### Video validation

We registered two main hypotheses to be tested as validation (see https://osf.io/ut6xm for registration). First, we hypothesized that “[v]ideos including targets shedding tears are perceived as expressing more sadness and helplessness compared to videos without tears.” Second, we hypothesized that “[v]ideos including exaggerated expressions are perceived as more intense than natural expression videos.” We tested both hypotheses using multilevel models with participants nested in countries and videos nested in actors as random intercepts (detailed models Supplementary Section 4.2, Supplementary Tables 9-11).

**Fig. 4:**
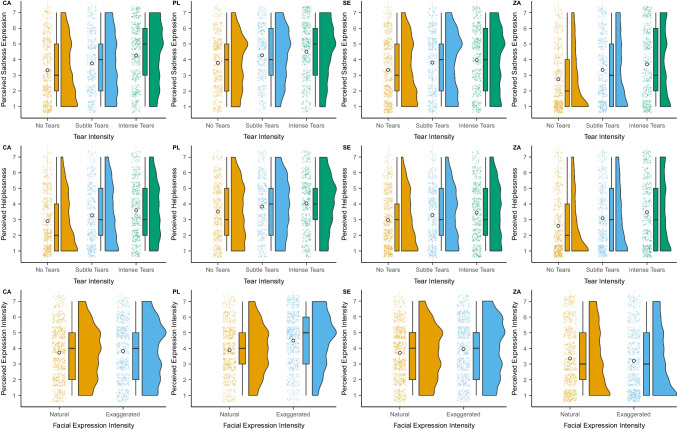
Overview of perception ratings (perceived sadness expression, perceived helplessness, and perceived expression intensity) for different types of tear intensity and facial expression intensity across the four samples. CA = Canada, PL = Poland, SE = Sweden (Scandinavia), ZA = South Africa. Raincloud plots show a) individual data points, b) boxplots with median (thick horizontal line), lower, and upper quartiles, and c) data distributions. White dots indicate descriptive means

Videos including subtle (Sadness Expression: *M* = 3.78, *SE* = 0.23, Perceived Helplessness: *M* = 3.36, *SE* = 0.20) or intense tears (Sadness Expression: *M* = 4.12, *SE* = 0.23, Perceived Helplessness: *M* = 3.65, *SE* = 0.20) were rated higher on perceived sadness of the expression and perceived helplessness compared to videos without tears (Sadness Expression: *M* = 3.29, *SE* = 0.23, Perceived Helplessness: *M* = 3.00, *SE* = 0.19). Tearful videos (subtle or intense) were rated significantly higher in sadness expression and perceived helplessness compared to videos without tears (Sadness Expression: *d* = 0.51 [0.39, 0.63], *t*(470.97) = 8.32, *p* = < 0.001; Perceived Helplessness: *d* = 0.41 [0.28, 0.53], *t*(467.27) =6.52, *p* < 0.001). Similarly, intense tear videos were perceived as higher in sadness expressions compared to subtle tear videos (Sadness Expression: *d* = 0.17 [0.08, 0.27], *t*(479.27) = 3.50, *p* < 0.001; Perceived Helplessness: *d* = 0.15 [0.06, 0.25], *t*(475.30) = 3.10, *p* = 0.002; Figure 4).

For facial expression, we observed that exaggerated facial expressions were perceived as more intense (*M* = 3.87, *SE* = 0.22) compared to natural facial expressions (*M* = 3.66, *SE* = 0.22), *d* = 0.11 [0.04, 0.19], *t*(477.29) = 2.97, *p* = 0.003. Comparisons across samples revealed that exaggerated expressions were rated as significantly more intense for the Polish sample (*d* = 0.35 [0.16, 0.55]), non-significantly more intense for the Swedish (*d* = 0.14 [−0.02, 0.31]), and Canadian samples (*d* = 0.08 [−0.12, 0.27]), but actually less intense for the South African sample (*d* = −0.08 [−0.27, 0.11]). In general, intensity ratings in the South African sample were lower (*M* = 3.28, *SD* = 1.99) compared to the other three samples (CA: *M* = 3.78, *SD* = 1.79, PL: *M* = 4.19, *SD* = 1.72, SE: *M* = 3.82, *SD* = 1.79).

## Discussion

Across more than 2,500 participants and 16,000 video ratings, we provide a first validation of the ECBD focusing on variations in tear intensity, facial expression intensity, body movements (face touching), vocalizations, and the temporal dynamics of crying. Altogether, we were successful at differentiating across these factors. Binary manipulations (absence vs. presence of behavior) showed typically stronger effect sizes compared to continuous manipulations (intensity of behavior). Further, we validated the *tearing effect*, the tendency of tearful individuals to be perceived as sadder and more helpless, across our stimuli and different samples.

In Study 2, we found a weaker effect of differentiating between facial expression intensity levels. This could be attributed to a different item formulation, focusing on overall expression intensity instead of facial expression intensity or cross-cultural differences, suggesting that the overall expression intensity might be evaluated differently in South Africa than in other countries we included. At the same time, the non-intuitive direction of this effect in the South African sample, showing that exaggerated expressions were rated as less intense than natural facial expressions, might equally suggest that participants in the South African sample completed the study less carefully (for instance, the number of failed video comprehension checks was also the highest in this subsample).

As expected, we also observed variation across the different actors. We provide norming data to possibly select only the highest-rated stimuli, but we should also note that such variation mirrors between and within variance in real-life emotional expressions. One possible disadvantage of the current dataset might be that it can be difficult to implement in contexts where static images are preferable. For this reason, we also performed an additional validation of static images obtained from the dynamic stimuli.

## Picture dataset development & validation

We supplemented the video dataset by developing static stimuli. This was done for situations in which a researcher wants to assess different crying features but does not have the possibility to use dynamic stimuli due to reasons such as limited internet availability or connection, time constraints, or using a pen-and-paper questionnaire. Since dynamic and temporal developments are difficult to depict using static images, this dataset should be considered a supplement to the main dataset.

### Picture stimulus development

Pictures were taken from the video stimuli, which were described earlier. Based on the dynamic nature of some of them, we only focused on the features that could be presented in a static form, that is, the intensity of tears (no tears, subtle tears, intense tears) and the intensity of facial expressions (natural, exaggerated). This resulted in six possible combinations. We added a control comparison (with all factors absent), thus resulting in seven combinations for each actor and 70 stimuli in total (for examples, see Figure 1). Each of the eligible 250 videos was screened by two of the authors, and stills were created for frames that were considered to represent the two factors with the highest precision. At least two pictures were created for each of the 70 combinations. These selected stimuli were evaluated by three independent judges with the aim of choosing one final picture. The final 70 pictures were tested in a first validation round, including 301 participants (Study 3a). An overview of the procedure and results is provided in the Supplementary Section 5.

Based on this validation study, we identified pictures that were rated higher/lower than what would be expected. We used a mean rating of 5 as a cutoff, identifying pictures categorized as showing *subtle tears* that were rated higher than 5 on tear intensity and pictures categorized as showing *intense tears* rated lower than 5 on tear intensity. For facial expression, we identified pictures categorized as a *natural expression* rated higher than 5 on expressiveness and pictures categorized as an *exaggerated expression* rated lower than 5 on facial expressiveness. This resulted in 20 pictures, on average, not rated as intended. For these pictures, alternatives were selected in a second round of sourcing static images. All final images were aligned as best as possible by a professional graphic designer with regard to quality, framing, and brightness. The 50 images from round 1 and the updated 20 images were then validated in an additional study as described below.

### Picture validation: Study 3b

#### Method

##### Participants 

We registered to obtain at least 20 ratings per picture. Since the total pool consisted of 70 pictures and, to minimize fatigue, each participant rated five pictures, around 280 participants were needed. To account for possible exclusions and random distribution we registered to recruit 300 participants.

A total of 311 UK-based participants were sampled via Prolific.com for a 6-minute study with £0.75 as compensation. Eleven participants were excluded because they failed an attention check. The final sample size included 300 participants (149 males, 146 females, 3 non-binary, 2 other) ranging from 18 to 84 years of age (*M* = 40, *SD* = 13.1). The majority (*n* = 262) indicated a UK nationality. On average, 21.43 (*SD* = 3.82) ratings were obtained per picture (8 to 30 ratings per picture; 3 pictures with less than 15 ratings). In total, the dataset included 1500 ratings.

##### Design & procedure

 We employed a 10 (actor) x 3 (tears: no tears vs. subtle tears vs. intense tears) x 2 (expression: natural vs. exaggerated) mixed design. This resulted in 60 possible combinations, and we also included one control picture per actor, thereby having 70 possible combinations and pictures in total.

After providing informed consent, participants were presented with instructions. Participants were shown five pictures of the total pool of 70 pictures (development described above). For each participant, 5 of the 10 actors were randomly chosen, and for each actor, it was randomly determined which combination of factors would be presented. After the last picture, participants were presented with an attention check (“Please select ‘3’ on the scale”). Finally, participants completed information regarding their gender, age, and nationality.

##### Measures

For each picture, participants completed the same 13 items in fixed order. First, participants completed validation measures asking about the intensity of tears (“This person… is not shedding tears at all” (1)/“is shedding subtle tears” (4)/“is shedding intense tears” (7)) and facial expression intensity (“This person’s facial expression is… subtle” (1)/“moderate” (4)/“exaggerated” (7)). Afterward, participants completed the same additional items as in Study 2 on expression (including overall expression intensity), inferences, felt emotions, and social support intentions.

### Results

#### Norming data

 Norming data for all pictures can be accessed at https://osf.io/udt7h.

#### Picture validation

**Fig. 5:**
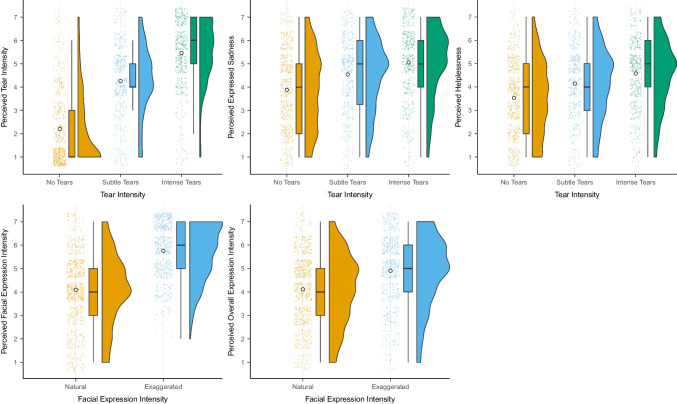
Overview of perception ratings across all participants (*N* = 1,500) for five different variables: perceived tear intensity, perceived expressed sadness, perceived helplessness, perceived facial expression, perceived overall expression intensity. Raincloud plots show a) individual data points, b) boxplots with median (thick horizontal line), lower, and upper quartiles, and c) data distributions. White dots indicate descriptive means

To check the validation of the pictures, we first conducted five multilevel models. The first three models included tear intensity as the predictor (contrasts coded: -${~}^{2}\!\left/ \!{~}_{3}\right.$ no tears, ${~}^{1}\!\left/ \!{~}_{3}\right.$ subtle tears, ${~}^{1}\!\left/ \!{~}_{3}\right.$ intense tears; 0 no tears, -.5 subtle tears,.5 intense tears) and perceived tear intensity, perceived expressed sadness, and perceived helplessness as outcomes. The other two models included facial expression as the predictor (natural vs. exaggerated) and perceived facial expression and perceived overall expression intensity as outcomes. For all models, we added participants as random effects and videos nested in actors. An overview of ratings is presented in Figure 5 and detailed models are presented in Supplementary Tables 17-21. For tear intensity, we observed that pictures that should show *subtle tears* (*M* = 4.27, *SE* = 0.15) and *intense tears* (*M* = 5.54, *SE* = 0.15) were rated as higher in tear intensity than pictures that should show no tears (*M* = 2.15, *SE* = 0.13, *d* = 1.34 [1.21, 1.48], *t*(58.38) = 19.43, *p* < 0.001). Similarly, pictures that should show *intense tears* were rated as higher in tear intensity than subtle tear pictures (*d* = 0.62 [0.44, 0.80], *t*(56.83) = 6.90, *p* < 0.001). Pictures including subtle (*M* = 4.56, *SE* = 0.18) or intense tears (*M* = 5.06, *SE* = 0.18) were rated as expressing more sadness than pictures with no tears (*M* = 3.84, *SE* = 0.17, *d* = 0.58 [0.42 0.75], *t*(58.01) = 6.92, *p* < 0.001). At the same time, intense tears were perceived as expressing slightly more sadness than subtle tear pictures (*d* = 0.30 [0.08, 0.51], *t*(56.30) = 2.72, *p* = 0.009). We also observed that actors with subtle (*M* = 4.16, *SE* = 0.15) or intense tears (*M* = 4.57, *SE* = 0.15) were rated as more helpless than actors with no tears (*M* = 3.50, *SE* = 0.13, *d* = 0.54 [0.39, 0.68], *t*(56.97) = 7.29, *p* < 0.001). At the same time, actors with intense tears were rated as significantly more helpless than actors with subtle tears (*d* = 0.25 [0.06, 0.44], *t*(54.76) = 2.61, *p* = 0.012).

We also observed that videos including actors showing an exaggerated facial expression were rated higher in perceived facial expression intensity (*M* = 5.81, *SE* = 0.14) than actors instructed to show a natural facial expression (*M* = 4.05, *SE* = 0.13; *d* = 1.09 [0.86, 1.31], *t*(58.44) = 9.51, *p* < 0.001). Similarly, the overall expression intensity of actors instructed to show an exaggerated facial expression was perceived as more exaggerated (*M* = 4.94, *SE* = 0.14) compared to actors instructed to show a natural expression (*M* = 4.05, *SE* = 0.13, *d* = 0.56 [0.36, 0.76], *t*(58.07) = 5.47, *p* < 0.001). Compared to the first picture validation round, overall differences in subtle and intense tears and natural and exaggerated expressions were stronger (Supplementary Material Section 5).

**Fig. 6:**
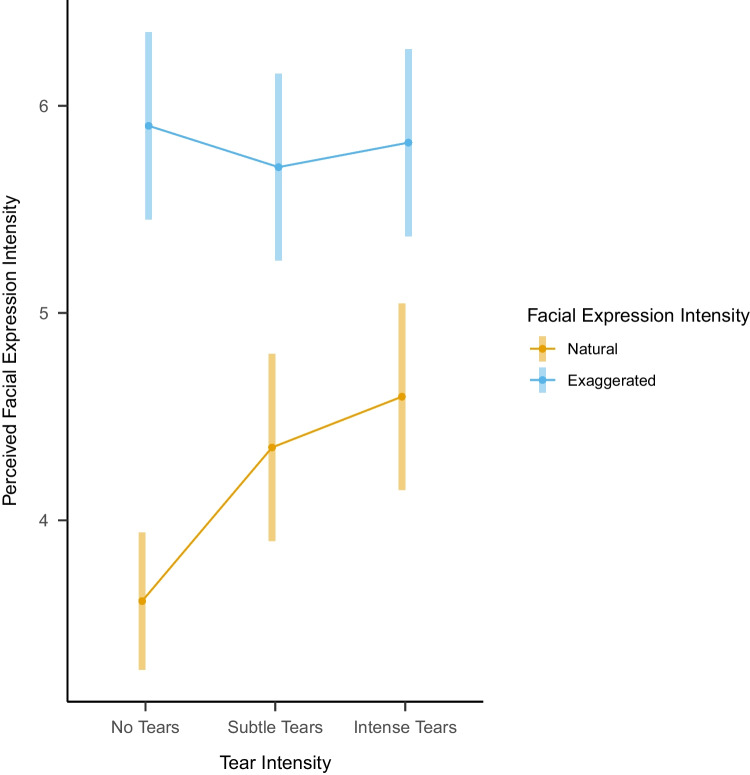
Overview of interaction between tear intensity level and facial expression intensity for ratings of perceived facial expression intensity using estimated means. Error bars represent 95% confidence intervals

**Fig. 7:**
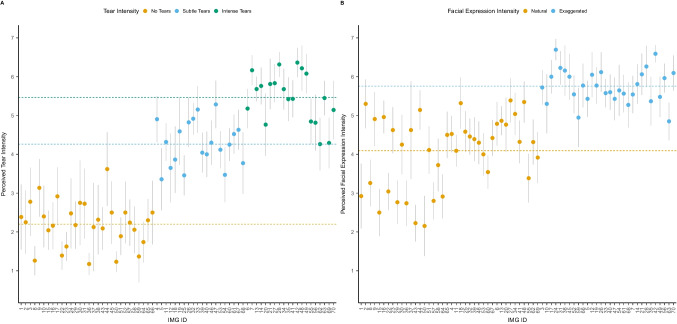
Overview of perception ratings of tear intensity (**A**), facial expression (**B**). Dots represent means and lines 95% CIs. Colored dashed lines represent respective subgroup means

We also explored the interaction between tear intensity and facial expression for each of the ratings. We observed a statistically significant interaction between facial expression intensity and tear intensity for the perceived facial expression intensity rating (η2(2) = 8.37, *p* = 0.015). Tears, whether subtle or intense, increased perceived facial expression intensity ratings only for natural facial expressions but not for exaggerated facial expressions (Figure 6). Yet, given that facial expression intensity for exaggerated facial expressions was scored very high, it is possible that tears did not increase these ratings due to a ceiling effect. An overview of ratings for perceived tear intensity and perceived facial expression intensity per picture is provided in Figure 7.

Further, we computed a dichotomous variable for each perception rating, indicating the absence (below the mean) or presence of the variable (above the mean). For tear intensity, we coded subtle tears as matching for a rating between 2 and 4 and intense tears for a rating between 4 and 7 (this was not registered). Again, we observed medium matching for facial expression intensity and tear intensity. First, facial expression intensity matched on average in 70% of ratings (*SD* = 45.84), while tear intensity matched on average for 57.93% of ratings (*SD* = 49.38). A detailed overview of matching per video and actor is provided in Supplementary Figure 14. Again, validation was most difficult for subtle tear pictures that showed the lowest match percentage. Matching percentages increased slightly from the first picture validation round (2.32pp for tear intensity, 3.49pp for facial expression intensity). A detailed comparison between ratings for pictures in validation round 1 and validation round 2 is provided in Supplementary Figure 15.

Finally, we explored inter-rater reliability as in Study 1, and detailed results are provided in Supplementary Section 6.2. We observed acceptable inter-rater reliability for the majority of pictures but noticed low inter-rater reliability for a small portion of pictures (13 out of 70; Supplementary Table 22).

## General discussion

Emotional crying is a complex and multifaceted expression consisting of different features. However, there is a strong lack of research investigating perceptions and responses to different features of emotional crying and their combination (Krivan & Thomas, 2020). Here, we provide a validation of the Emotional Crying Behavior Dataset (ECBD), enabling researchers to study the complex interactions of emotional crying features using experimentally controlled stimuli. Thereby, the ECBD addresses four main gaps in the literature by a) providing systematic variation in features beyond emotional tears, b) enabling an experimental test of a combination of different crying features, c) allowing for a test of temporal dynamics in emotional crying, and d) providing a systematic validation of the stimuli across two studies and different samples.

### The ECBD

The validation supports the manipulation of the different features. Intense tears were perceived as more intense, exaggerated facial expressions were perceived as more exaggerated, and participants correctly perceived vocalizations, gestures such as face touching, and temporal dynamics such as a sudden stop of crying. Notably, the features that were manipulated as either present or absent (vocalization, gesture, temporal dynamics) elicited stronger effects across videos compared to features that varied in their level of intensity (i.e., tear and facial expression intensity). We acknowledge that performing binary judgment on whether a behavior is absent or present is easier than judging the intensity of a behavior. In line with this reasoning, we observed that participants found it easier to differentiate between stimuli showing no tears vs. subtle tears than between stimuli showing subtle vs. intense tears, providing support for the notion that the intensity of tears is better described as a continuum than a dichotomous variable (see Küster, 2018). Of importance here, we operationalized the intensity of tears as their quantity (1-2 tears vs. several tears), and when internally validating the stimuli, we paid careful attention to whether this condition was met. On the one hand, this enabled us to ensure that tears were well visible, but on the other, finding the right balance between the visibility of tears and their subtleness was somewhat challenging. One reason for that might be that we employed standardized conditions (in particular, good lighting and frontal view) and water droplets, due to which, in many cases, even “subtle” tears looked quite pronounced, which is a frequently mentioned characteristic of posed emotional expressions in general (Cong et al., 2025; Krumhuber et al., 2021). At the same time, we believe this good visibility of tears makes our dataset useful for machine-learning approaches to visual tear detection (e.g., Küster et al., 2022a, 2022b).

Further, effects slightly varied across different actors, which is a common observation in stimulus sets (Zickfeld et al., 2018), mirroring the variance in people’s physical appearance and expressivity observed in real life (Satchell et al., 2023). For example, a study trying to replicate an effect of people crying with tears being perceived as less competent found that the initial effects were most likely stimulus-specific, highlighting the need for multiple and diverse stimuli (Zickfeld et al., 2018). The same holds true for the current dataset. For generalization purposes, we recommend always using varied stimulus sets rather than basing any tests or conclusions on single stimuli (Satchell et al., 2023). Our validation provides strong evidence for the successful manipulation of features across all stimuli and actors. To account for variation in stimuli and actors, studies are recommended to employ multilevel methods (Judd et al., 2012; Küster et al., 2022a, 2022b). For instances in which presentation time is limited and only a few stimuli can be selected, we provide norming data that allow researchers to select stimuli scoring highest or lowest on a variable of interest to maximize the effectiveness of the experimental manipulation.

Further, our validation confirmed that the current stimuli produce the *tearing effect*, the common finding that individuals shedding tears are perceived as sadder or helpless (e.g., Balsters et al., 2013; Küster et al., 2022a, 2022b; Ong & Ito, 2022; Reed et al., 2019). This is a crucial observation, suggesting that our stimuli are perceived as intended not only in terms of behavioral features but also in terms of their social signal value (for details, see a recently proposed social glue model of tears; Zickfeld & Wróbel, 2024). We also observed acceptable to high inter-rater reliability for the majority of stimuli in the dataset. Variation in raters also mirrors the variance of evaluations in real life. Importantly, if researchers opt to employ single instead of varied stimuli from the dataset, we recommend taking into account inter-rater reliability statistics and using stimuli with high levels of inter-rater reliability.

We extended the dataset of dynamic stimuli by developing a static picture-based variant sourced from the dynamic stimuli. Due to the dynamic nature of the original stimuli, we focused on two main features, namely, tear and facial expression intensity, that could be reliably depicted in a static form. Even though this is somewhat limited, there exists no static image resource that experimentally manipulates and crosses these two factors (Table 1.), providing evidence for the need for such a validated resource. The main purpose of this supplementary static picture resource would be for research contexts that aim to test different features of crying but do not have the capacity or possibility to use dynamic stimuli, such as in situations with a limited internet connection or when using pen-and-paper questionnaires. We successfully validated the static stimulus dataset; however, validation was more difficult compared to the dynamic stimuli. Especially for tear intensity, many stimuli needed to be re-done and re-evaluated to reach satisfactory levels of discrimination, lending additional support for the continuous nature of this feature (Küster, 2018). Again, we provide detailed norming data for each stimulus that might guide in selecting the most effective pictures if needed. This also highlights that the experimental strength of the dynamic stimuli is superior and points to a general observation that for complex and multifaceted expressions such as emotional crying, the use of dynamic stimuli is inevitable. The current dataset aids researchers in achieving such a level of experimental control.

### Limitations and future directions

Recent calls have highlighted the need for spontaneous and natural stimuli in emotion expression (Krivan & Thomas, 2020; Küster et al., 2022a, 2022b). We agree with this, but relied on posed expressions for the current dataset for two main reasons. First, the primary focus of the set was on different crying features and providing high internal validity and experimental control to distinguish among these features and a combination of them. The more features were present in the current stimuli, the more difficult it was to effectively discriminate among features, especially those focusing on intensity. Spontaneously generating crying features under controlled circumstances is extremely difficult (Vingerhoets & Bylsma, 2016). Individuals will use different crying features depending on the trigger and the context (Barthelmäs et al., 2024). Instructing them to only depict specific features will strongly impact the spontaneousness of the response and possibly reduce the perceptions of the authenticity of crying. To tackle this problem and to successfully develop stimuli that differ in crying features or include a combination thereof, we think that using professional actors posing these features was the most feasible and valid option. While beliefs of being manipulated by crying are an important concern (Ten Brinke et al., 2012; Van Roeyen et al., 2020), people are, on a frequent basis, influenced by the displays of professional actors when consuming movies or TV series. From an ethical perspective, actors needed to display tears for more than 40 instances. These were often recorded repeatedly, making it nearly impossible to expect someone genuinely (and controllably) crying that many times while keeping the production feasible.

Second, the posed nature of the current dataset also allows its juxtaposition with different situational contexts, such as more positive situations. Most spontaneous resources focus on inducing crying in response to negative or sad situations (Küster et al., 2022a, 2022b), and it might be difficult to use these stimuli when studying the perception of tears of joy. The targets in our dataset were not instructed to show specific emotions, and no specific emotions were induced in them. Hence, even though most of them portrayed sadness-like expressions, there is some variety in this regard, making it possible to sample stimuli with expressions of more positive emotions. To make such sampling more efficient and valid, future studies might supplement our validation efforts by systematically coding the targets’ facial activity using automated FACS coding (Bartlett et al., 2004) and providing normative ratings of this activity.

Altogether, the current dataset has a strong focus on experimental control of the different crying features. Thereby, the ecological validity of the stimuli is reduced. We think this presents an important limitation, but we see an easy solution to this problem. If a researcher is interested in experimental control of different crying features, the current dataset provides a valid opportunity. However, if a researcher is interested in less experimental control but more spontaneous and ecologically valid stimuli, the availability of the validated PDSTD (Küster et al., 2022a, 2022b) can address this need. In the end, a valid and reliable study of emotional crying requires both experimental control and ecological validity, which affords the existence of both datasets as they appeal to different needs. We also think that future research on crying, similar to studies on emotional expression in general (Cong et al., 2025; Krumhuber et al., 2021), should systematically compare posed and spontaneous depictions of crying to elucidate possible effects these two types of stimuli might have on the overall perception of the crier. For instance, the already mentioned visibility of tears, which is likely much higher for posed than spontaneous crying expressions, might affect the perceptions of not only expression intensity but also authenticity (Zickfeld & Wróbel, 2024). Cross-dataset comparisons could also help address the issue of how to best operationalize the difference between subtle vs. intense tears. Here, we employed the operationalization based on the number of tears, but it is possible that other tear characteristics (e.g., their specific flow or reflectivity) might contribute to perceptions of intensity as well.

Similar to previous dynamic datasets (Küster et al., 2022a, 2022b), our stimuli contain no verbal information, so they can be used in various cultural and language contexts. However, they are limited on factors such as ethnicity and age. All targets in the current dataset are White and of a younger age. Extending such resources to different ethnicities and infants, children, or older adults is essential in the future to provide a more comprehensive picture of emotional crying and increase the generalizability of studies relying on these resources.

Another limitation, also pertaining to other stimuli (Table 1), is that our targets are not shown in any specific context (all actors are presented against a white, standardized background), making it difficult to test how the target’s expression interacts with contextual factors (except those inherently linked to the actors themselves such as their gender or physical appearance; Hess & Hareli, 2016). Yet, it is possible to provide such contextual information, starting from as simple (but not ideal) additions as verbal descriptions or vignettes and ending with more ecologically valid solutions such as designing 3D virtual humans based on our stimuli and placing them in specific contexts (Bartl et al., 2021).

Finally, due to randomization of stimuli, not all stimuli received the same number of raters, which might also affect individual inter-rater reliability. Indeed, a small portion of the dataset exhibited low inter-rater reliability. Nevertheless, by far the majority showed good to high inter-rater reliability, and variations in raters’ evaluations reflect variance in real-world contexts. As detailed earlier, our recommendation is to always use varied stimulus sets, which should overcome issues with low inter-rater reliability. If researchers can only rely on single or a few stimuli, we recommend selecting stimuli showing high discrimination and inter-rater reliability.

## Conclusion

Despite these limitations, we think that the ECBD will provide a valuable tool to researchers studying emotional crying. As the first comprehensive dataset, it allows researchers to experimentally test the influence of different crying features on perceptions and outcomes. For example, studies focusing on single features have sometimes observed contradicting outcomes (Zickfeld & Wróbel, 2024). The dataset allows researchers to study such features not only in isolation but also in combination with each other, providing a more valid picture of crying behaviors in real life. As emotional crying is a multifaceted response (Barthelmäs et al., 2024), the field needs to move beyond a focus on single features. The ECBD provides a first comprehensive starting point to investigate different features and move the field towards a more comprehensive approach.

## Supplementary Information

Below is the link to the electronic supplementary material.

Supplementary file1 (DOCX 6085 KB)

## Data Availability

All data are available at https://osf.io/g63ny/

## Footnotes

1 Emotional crying or emotional tears are differentiated from *basal* tears that lubricate the cornea and *reflex* tears due to irritation of the eye (Murube, 2009).

2 Researchers willing to use the ECBD should go to https://zenodo.org/records/15147817 and request permission to access the files, stating their (1) affiliation and (2) the intended use of the dataset.

3 We did not systematically code the actors’ facial expressions but noticed this feature when we internally validated the videos (which required watching them several times). Most actors portrayed sadness by adopting prototypical characteristics of this expression (downturned mouth and increased activation of corrugator muscles).

4 Model failed to converge for the sudden stopping predictor. We refitted the model using the Nelder-Mead optimization, which solved convergence issues. Note, that this step was not preregistered.

5 Note that we originally registered to exclude participants spending less than 15 s on the page but realized that many videos were shorter than this number, so we used video length as a better indicator of whether participants stayed on the page for the full time of the video.

## Code availability

All syntaxes are available at https://osf.io/g63ny/
