# Supplementary material for: The Emotional Crying Behavior Dataset (ECBD): A comprehensive resource to study the multifaceted nature of emotional crying
Source: Behav Res Methods. 2025 Sep 10;57(10):281. doi: 10.3758/s13428-025-02766-4 (PMC12423160; doi:10.3758/s13428-025-02766-4)
Supplement: Supplementary file 1 — Supplementary file1 (DOCX 6085 KB) [file 13428_2025_2766_MOESM1_ESM.docx]

**The Emotional Crying Behavior Dataset (ECBD):**

**A comprehensive resource to study the multifaceted nature of emotional crying**

**Supplementary Material**

**Section 1 – Preliminary Studies**

We conducted two preliminary studies to inform the selection of crying features employed in the dataset. Both studies were originally conducted for a different purpose, and they are described here for completeness. Data, analyses scripts, and materials for the two preliminary studies are available here: <https://osf.io/nvjfe/>

**1.1 Preliminary Study 1**

***Method***

**Participants.** We recruited 194 Polish participants (144 women, 45 men, 5 other), using convenience sampling. Their age ranged from 17 to 58 years (*M* = 26.06, *SD* = 7.37). All participants provided informed consent and were not paid for participation.

**Procedure and Materials.** The study was conducted online. After providing informed consent and information about their age and gender, participants were asked to recall a situation in which they experienced being manipulated with tears/crying. Those unable to recall such a situation were asked to indicate they had no such experiences. The remaining participants were asked open questions about the motivation of the crier (“What do you think the motivation of the crier was?”) and the expressive behaviors of the crier (“How did you recognize that the crying was used for manipulative purposes? Did they use any specific behaviors?”). They were also asked to rate

how likely the crier was, in general, to manipulate others, look for other people’s attention, and lie to others. Answers were given on a scale from 1 (*not at all*) to 7 (*very much*).

After providing answers to these questions, participants were asked to recall a situation in which they used tears/crying to manipulate others (again, those unable to recall such a situation were asked to indicate that they had no such experiences). They were also asked two questions regarding their motivation (“What was your motivation?”) and expressive behaviors (“Did you use any specific techniques/behaviors?”). Following that, participants were provided with a short explanation regarding the aim of the study.

***Results***

**Being Manipulated with Crying.** A total of 115 (59.30%) participants declared that they had experienced being manipulated with tears/crying. Their responses to open questions were subjected to content analysis performed by two coders. The aim of the analysis was to identify common patterns in participants’ answers. When asked about the motivation of the crier, participants focused on the following themes: asking someone for favor/money/other goods (24.35%), evoking compassion (11.30%), achieving forgiveness (10.43%), making someone feel guilty (8.70%), getting attention (8.70%), avoiding social responsibility for doing something wrong (8.70%). Some also mentioned that the crier used crying to change someone’s behavior towards them (8.70%), but this motive was limited to romantic relationships (e.g., “to get back together after we’ve split up”). Finally, some participants said the crier’s motivation was to change someone’s mind/decision (16.52%), but responses representing this theme were very general and overlapped with other themes.

When asked about the factors that made them realize that tears were used for manipulative purposes, participants most frequently pointed to the sudden/unexpected stop of crying (15.65%), acoustic signals such as sobbing or wailing (13.91%), face touching, e.g., wiping tears, covering the eyes/face (7.83%), and exaggerated/theatrical facial expression/grimace (6.96%). Less frequent themes were lack of tears (3.48%), change to another emotional state (2.61%), and sudden/unexpected start of crying (2.61%).

**Manipulating with Crying.** Ninety-eight (50.50%) participants declared that they had also used tears/crying to manipulate others. The content analysis showed that participants’ motives represented the same themes as those identified in the first recall task. These included themes such as evoking compassion (15.31%), asking someone for favor/money/other goods (13.20%), getting attention (12.20%), achieving forgiveness (8.16%), making someone feel guilty (7.14%), and avoiding social responsibility for doing something wrong (7.14%). Two frequent motives were also changing someone’s behavior (10.20%) and changing someone’s mind (13.26%), but responses representing these themes either referred to exclusively romantic relationships or were too general. When asked about the specific techniques/behaviors participants used to evoke tears, most of them said they recalled a situation that made them cry in the past (17.35%) However, no participants provided examples of specific expressive behaviors associated with their manipulative crying (except one who mentioned acoustic signals).

**1.2 Preliminary Study 2**

***Method***

# **Participants.** We recruited a total of *n* = 250 participants (122 males, 125 females) on Prolific.com, ranging from 19 to 79 years of age (*M* = 40.58, *SD* = 12.47). A total of *n* = 238 were UK nationals (US: *n* = 10, other: *n* = 2). Participants were paid £1.30 for a 10-minute survey.

# **Procedure and Materials.** After providing informed consent, participants completed demographic items regarding gender, age, and nationality and were instructed that the present survey was interested in exploring the frequency of being manipulated with crying or tears. For that reason, participants were first asked to think of a situation from their past in which they “experienced being manipulated with crying/tears” and asked to recall the situation as clearly as possible. Then, they completed several items asking about the specific situation, the motivation of the crier, the crier, and specific expressive behaviors. After providing responses on being manipulated with crying/tears, participants were always asked to recall another situation in which they “used crying/tears to manipulate others”. Items were the same as for the previous situation, while referring to other targets as the *manipulee*. When asked about the specific situation in which they experienced being manipulated/manipulated themselves, participants indicated whether they could recall or had never experienced such a situation. In case participants reported they never experienced a situation the items referring to the different aspects of the situation were skipped.

First, participants were asked to provide a written description of the specific context of the situation in which they were manipulated/used crying or tears. Then, they were asked to indicate the motivation of the crier (either the other target or themselves, depending on the specific situation). Answer options included “get attention”, “evoked compassion/sympathy”, “ask someone for a favor/money/other goods”, “make someone feel guilty”, “avoid social responsibility for doing something wrong”, “achieve forgiveness”, and “other, namely…”. These items were informed based on the results of Preliminary Study 1 and participants were able to select multiple options that applied.

Afterward, we asked who was the crier/manipulee in the specific situation, including answer options “romantic partner”, “family member”, “close friend”, “colleague”, “acquaintance”, “stranger”, and “other, namely…”. Participants then completed how close they felt to the crier/manipulee on a 7-point scale from “not at all (1)” to “very much (7)”. Then, participants reported which expressions or behaviors the crier or they used for manipulation included options based on the findings of Preliminary Study 1: “sudden/unexpected stop of crying”, “acoustic signals (e.g., sobbing, wailing)”, “exaggerated/theatrical facial expression/grimace”, “face touching (e.g., wiping tears, covering eyes)”, “excessive flow of tears”, and “other, namely…”. Again, participants were able to select multiple options.

Finally, we asked how frequently participants encounter being manipulated with tears/manipulating with tears on a scale including “never,” “less than once a year,” “once a year,” “once a month,” “once a week,” and “daily.”

After completing both situations and their respective items, participants were presented with the 12 items (e.g., “I tend to lack remorse”) of the Dark Triad Dirty Dozen (Jonason & Webster, 2010) completed on a 7-point scale from *strongly disagree* (1) to *strongly agree* (7).

***Results***

**Being Manipulated with Crying.** A total of 223 participants (89.20 %) reported

that they could recall being manipulated with crying or tears. An overview of the specific frequency of experiencing being manipulated with crocodile tears is provided in Supplementary Figure 1. The majority of participants reported that being manipulated with crying occurred rarely, while 7.6% indicated that it might happen on a weekly or even daily basis.

Focusing on the crier or manipulator, the majority indicated that they felt manipulated by their romantic partner or a family member, most typically their child (see Supplementary Figure 2). This was reflected in the fact that the participants felt quite close to the crier (*M* = 4.49, *SD* = 2.02).

Focusing on the motivations of manipulating others with crying, an overview is presented in Supplementary Figure 3, A. The majority of participants who described a situation indicated that others used crying to evoke compassion (64%) or make the participant (or another person) feel guilty (56%). Getting attention (32%) or asking someone for a favor (30%) was reported less often but still prominent. The majority of participants selected more than one motivation (68.61%). The combination of evoking compassion and making someone feel guilty was mentioned frequently. Considering the expressive behavior of the crier (Supplementary Figure 3, C), the majority of participants providing a situation reported exaggerated facial expressions (60%) or face touching (59%). A smaller share mentioned a sudden stop of crying (41%) or acoustic signals (46%), whereas only about a third mentioned an excessive flow of tears being present (34%). Again, the majority of participants selected multiple expressions (76.23%). Participants mentioned most frequently that criers used acoustic signals and exaggerated facial expressions together.

**Manipulating with Crying.** A total of 82 participants (32.80 %) reported that they could recall manipulating others with crying or tears – far less than participants who could recall being the target of manipulation. An overview of the specific frequency of manipulating others with crocodile tears is provided in Supplementary Figure 1, B. The majority of participants reported that manipulating with crocodile tears is rarely used, with 23.20% using this tactic at least once a year. Similar to the previous situations of being manipulated with crying, participants reported using crying as a manipulation tool most frequently on their romantic partners or family members (74.39%, Supplementary Figure 2, B). Again, this was reflected in the fact that the participants felt close to their target of manipulation (*M* = 5.28, *SD* = 1.96).

Similar to situations of being manipulated, the majority of participants recalling a situation in which they manipulated others with crying reported evoking compassion as their main motivation (72%, Supplementary Figure 3, B), followed by making the other person feel guilty (37%). Asking someone for a favor (20%) and getting attention (13%) were also mentioned as potential motivations, but less frequently so. Again, the majority of participants selected more than one motivation (53.66%). Evoking compassion and making someone feel guilty were frequently mentioned as motivations occurring at the same time.

Finally, participants considered different expressive behaviors they used when manipulating others with crying (Supplementary Figure 3, D). Most frequently, they mentioned face touching (56%), an excessive flow of tears (48%), and an exaggerated facial expression (41%). In contrast to the situations about being manipulated, participants mentioned acoustic signals (30%) or a sudden stop of crying (18%) less frequently. It is possible that the latter is employed less deliberately but might occur more automatically once the specific goal is achieved. Again, the majority of participants selected more than one expression (60.98%). Exploring the relationship with expressive behaviors and motivations, we observed that face touching was frequently mentioned when the goal was to evoke compassion. Similarly, a sudden stop was often used when trying to get attention or making others feel guilty.

**Relation with Personality.** We explored the relationship between participants’ personality and the self-reported frequency of using crying to manipulate others. We observed a small positive correlation between self-reported frequency of manipulating others with crying and Machiavellianism, *rho* = 0.13. An overview is provided in Supplementary Figure 4. As high Machiavellianism is also associated with lower social trust, we explored the relationship between self-reported frequency of being manipulated with crying and Machiavellianism. We observed only a small positive correlation that was not statistically significant, *rho* = 0.06. In addition, we also explored the relationship between Machiavellianism and motivations and behaviors employed when manipulating others with tears. Machiavellianism correlated most strongly with using crying or tears to get attention and avoiding responsibility, and with using a sudden stop of crying (Supplementary Figure 5). Notably, the effects were small.

**Supplementary Figure 1**

*Overview of Frequency of Being Manipulated (A) or Manipulating (B) with Crying or Tears*


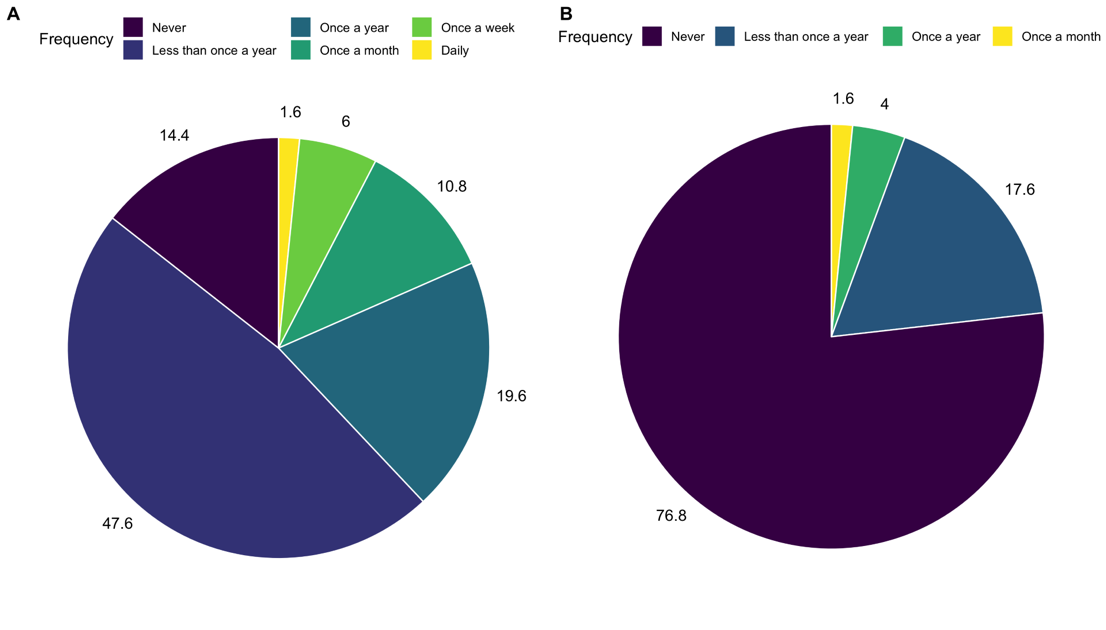


**Supplementary Figure 2**

*Overview of the Type of Person Manipulating (A) or Being the Target of Manipulation (B) with Crying or Tears*


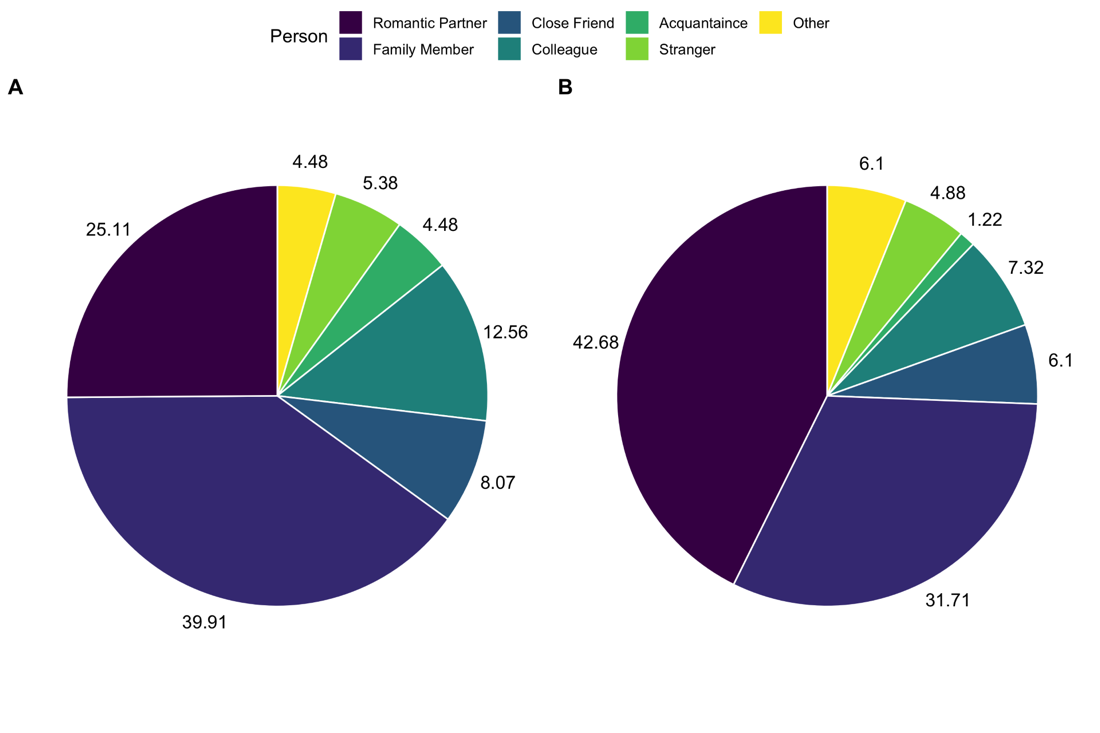


**Supplementary Figure 3**

*Overview of Frequency of Being Manipulated (A) or Manipulating (B) with Crying or Tears*


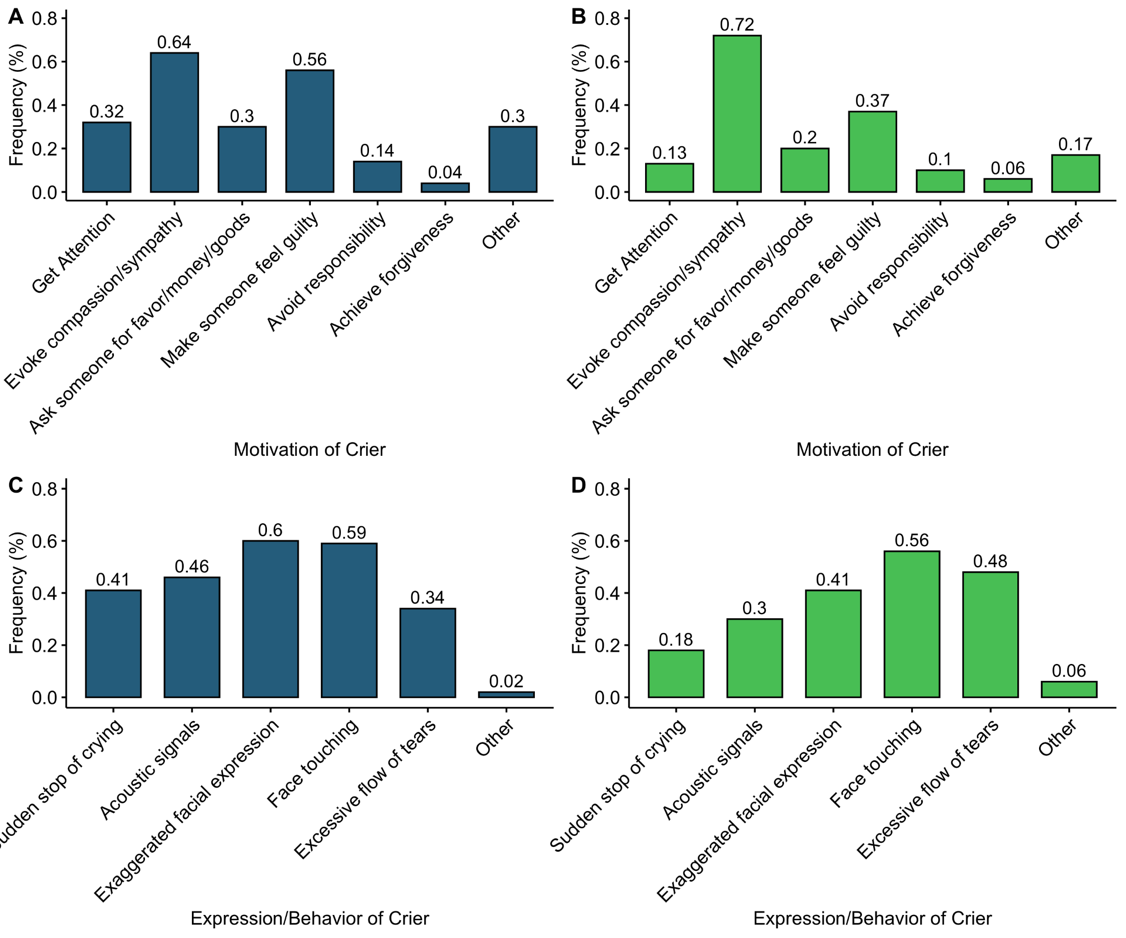


**Supplementary Figure 4**

*Scatterplot of the Relationship between Machiavellianism and the Frequency of Manipulating Others with Tears*

**
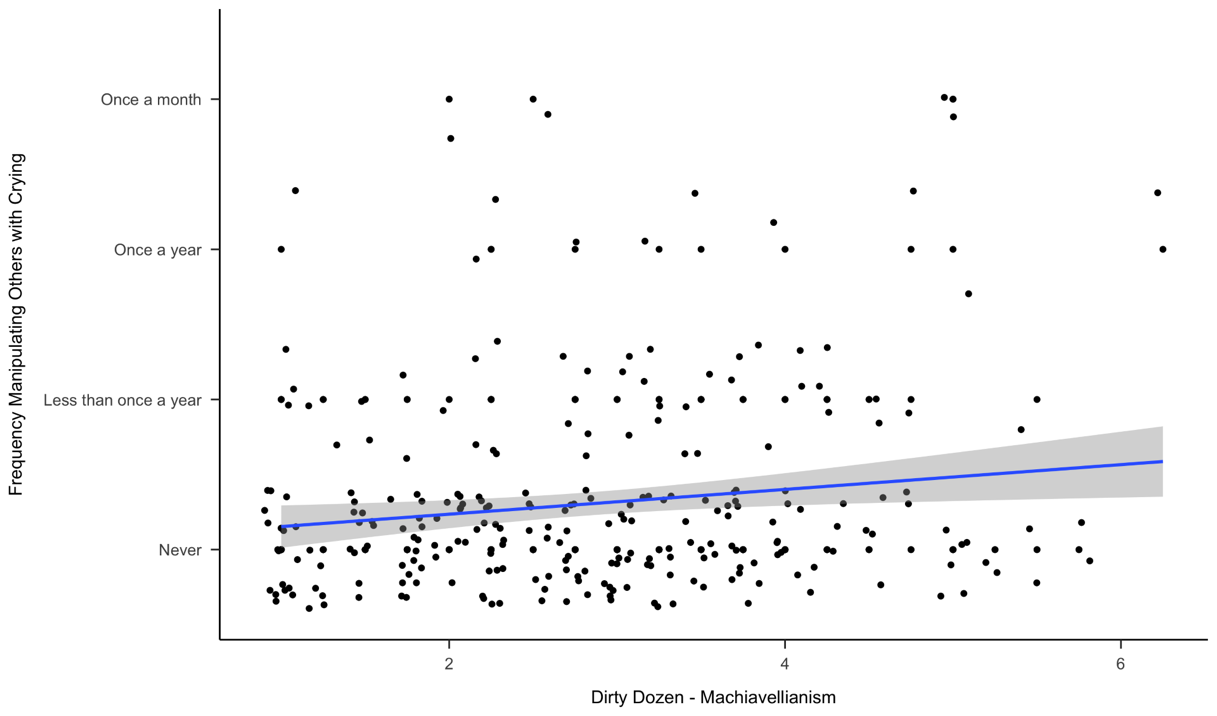
**

**Supplementary Figure 5**

*Correlation Plot of Point-Biserial Correlations Between Dark Triad Traits and Motivations and Behaviors of Manipulating Others with Tears*

**
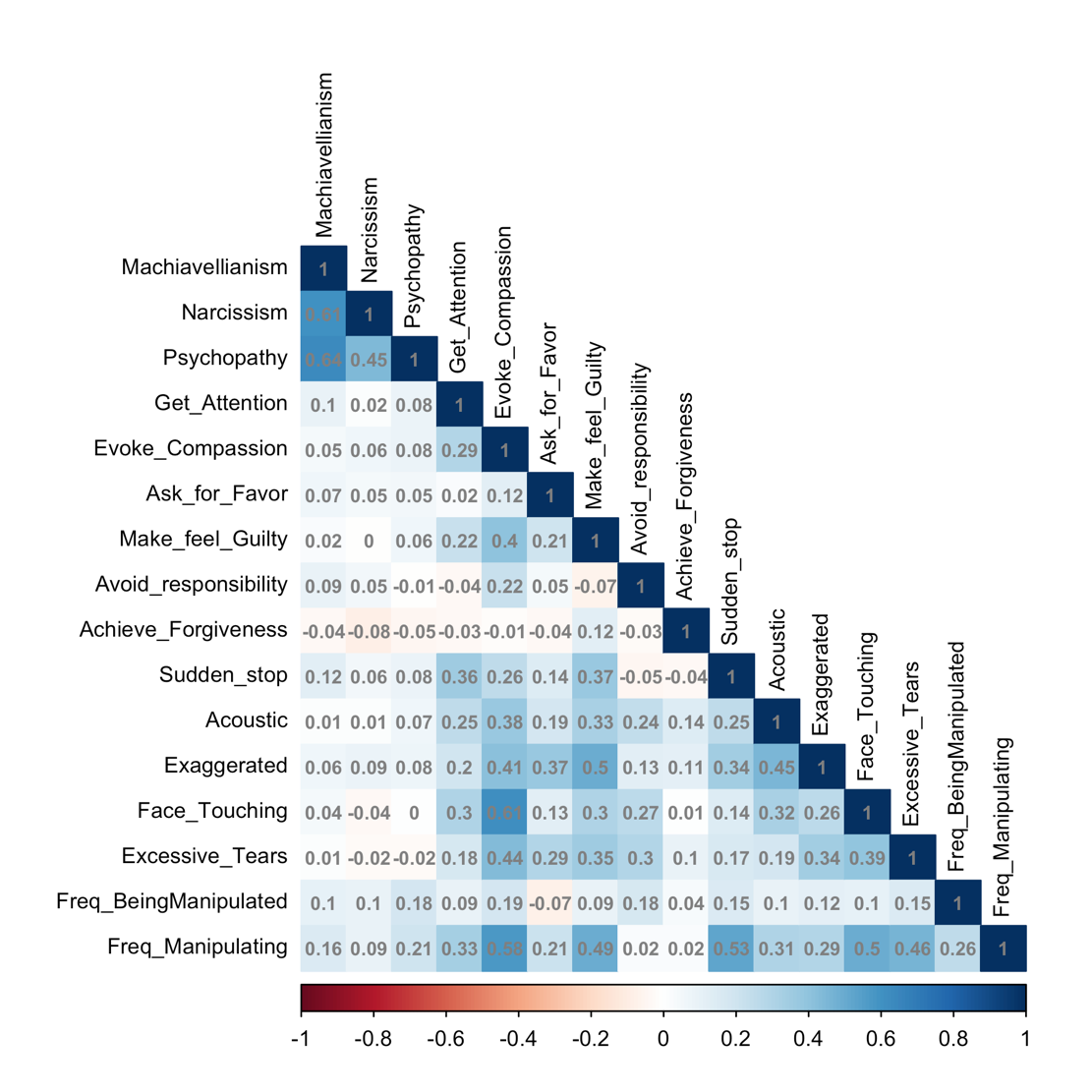
**

**Section 2 – Video Development**

Videos were produced by a film production agency (“315 Studio”, [https://315studio.pl](https://315studio.pl/)) that was provided with explicit instructions on the 50 combinations, detailed in Supplementary Table 1.

An overview of the internal video validation process for each actor and video is provided here: <https://osf.io/f3mc4>.

**Supplementary Table 1**

*Overview of the Combinations for Each Video*

| **Video ID** | **Tear Intensity** | **Facial Expression Intensity** | **Face Touching** | **Vocalizations** | **Sudden Stopping** |
| --- | --- | --- | --- | --- | --- |
| 1 | no tears | natural | no face touching (face fully visible) | silent crying | sudden stopping |
| 2 | no tears | exaggerated | no face touching (face fully visible) | silent crying | sudden stopping |
| 3 | no tears | natural | no face touching (face fully visible) | silent crying | gradual stopping |
| 4 | no tears | exaggerated | no face touching (face fully visible) | silent crying | gradual stopping |
| 5 | no tears | natural | no face touching (face fully visible) | sobbing/  wailing | sudden stopping |
| 6 | no tears | exaggerated | no face touching (face fully visible) | sobbing/  wailing | sudden stopping |
| 7 | no tears | natural | no face touching (face fully visible) | sobbing/  wailing | gradual stopping |
| 8 | no tears | exaggerated | no face touching (face fully visible) | sobbing/  wailing | gradual stopping |
| 9 | no tears | natural | face touching (face partially covered) | silent crying | sudden stopping |
| 10 | no tears | exaggerated | face touching (face partially covered) | silent crying | sudden stopping |
| 11 | no tears | natural | face touching (face partially covered) | silent crying | gradual stopping |
| 12 | no tears | exaggerated | face touching (face partially covered) | silent crying | gradual stopping |
| 13 | no tears | natural | face touching (face partially covered) | sobbing/  wailing | sudden stopping |
| 14 | no tears | exaggerated | face touching (face partially covered) | sobbing/  wailing | sudden stopping |
| 15 | no tears | natural | face touching (face partially covered) | sobbing/  wailing | gradual stopping |
| 16 | no tears | exaggerated | face touching (face partially covered) | sobbing/  wailing | gradual stopping |
| 17 | subtle tears  (1-2 tears) | natural | no face touching (face fully visible) | silent crying | sudden stopping |
| 18 | subtle tears  (1-2 tears) | exaggerated | no face touching (face fully visible) | silent crying | sudden stopping |
| 19 | subtle tears  (1-2 tears) | natural | no face touching (face fully visible) | silent crying | gradual stopping |
| 20 | subtle tears  (1-2 tears) | exaggerated | no face touching (face fully visible) | silent crying | gradual stopping |
| 21 | subtle tears  (1-2 tears) | natural | no face touching (face fully visible) | sobbing/  wailing | sudden stopping |
| 22 | subtle tears  (1-2 tears) | exaggerated | no face touching (face fully visible) | sobbing/  wailing | sudden stopping |
| 23 | subtle tears  (1-2 tears) | natural | no face touching (face fully visible) | sobbing/  wailing | gradual stopping |
| 24 | subtle tears  (1-2 tears) | exaggerated | no face touching (face fully visible) | sobbing/  wailing | gradual stopping |
| 25 | subtle tears  (1-2 tears) | natural | face touching (face partially covered) | silent crying | sudden stopping |
| 26 | subtle tears  (1-2 tears) | exaggerated | face touching (face partially covered) | silent crying | sudden stopping |
| 27 | subtle tears  (1-2 tears) | natural | face touching (face partially covered) | silent crying | gradual stopping |
| 28 | subtle tears  (1-2 tears) | exaggerated | face touching (face partially covered) | silent crying | gradual stopping |
| 29 | subtle tears  (1-2 tears) | natural | face touching (face partially covered) | sobbing/  wailing | sudden stopping |
| 30 | subtle tears  (1-2 tears) | exaggerated | face touching (face partially covered) | sobbing/  wailing | sudden stopping |
| 31 | subtle tears  (1-2 tears) | natural | face touching (face partially covered) | sobbing/  wailing | gradual stopping |
| 32 | subtle tears  (1-2 tears) | exaggerated | face touching (face partially covered) | sobbing/  wailing | gradual stopping |
| 33 | intense tears (several visible tears) | natural | no face touching (face fully visible) | silent crying | sudden stopping |
| 34 | intense tears (several visible tears) | exaggerated | no face touching (face fully visible) | silent crying | sudden stopping |
| 35 | intense tears (several visible tears) | natural | no face touching (face fully visible) | silent crying | gradual stopping |
| 36 | intense tears (several visible tears) | exaggerated | no face touching (face fully visible) | silent crying | gradual stopping |
| 37 | intense tears (several visible tears) | natural | no face touching (face fully visible) | sobbing/  wailing | sudden stopping |
| 38 | intense tears (several visible tears) | exaggerated | no face touching (face fully visible) | sobbing/  wailing | sudden stopping |
| 39 | intense tears (several visible tears) | natural | no face touching (face fully visible) | sobbing/  wailing | gradual stopping |
| 40 | intense tears (several visible tears) | exaggerated | no face touching (face fully visible) | sobbing/  wailing | gradual stopping |
| 41 | intense tears (several visible tears) | natural | face touching (face partially covered) | silent crying | sudden stopping |
| 42 | intense tears (several visible tears) | exaggerated | face touching (face partially covered) | silent crying | sudden stopping |
| 43 | intense tears (several visible tears) | natural | face touching (face partially covered) | silent crying | gradual stopping |
| 44 | intense tears (several visible tears) | exaggerated | face touching (face partially covered) | silent crying | gradual stopping |
| 45 | intense tears (several visible tears) | natural | face touching (face partially covered) | sobbing/  wailing | sudden stopping |
| 46 | intense tears (several visible tears) | exaggerated | face touching (face partially covered) | sobbing/  wailing | sudden stopping |
| 47 | intense tears (several visible tears) | natural | face touching (face partially covered) | sobbing/  wailing | gradual stopping |
| 48 | intense tears (several visible tears) | exaggerated | face touching (face partially covered) | sobbing/  wailing | gradual stopping |
| 49 | baseline | baseline | no face touching (face fully visible) | baseline | baseline |
| 50 | baseline | baseline | face touching (face partially covered) | baseline | baseline |

**Section 3 – Video Validation (Study 1) – Additional Analyses**

**3.1 Study 1 – Multilevel Models**

**Supplementary Table 2**

*Overview of Multilevel Model with Perceived Tear Intensity as the Outcome Variable and Tear Intensity (No Tears vs. Subtle Tears vs. Intense Tears) as the Predictor. Random Intercepts Based on Participants and Videos Nested in Actors. Left side shows the originally registered model and the right side shows the model with robust estimation using the robustlmm package.*

|  | **Perceived Tear Intensity**  **[Registered Model]** | | | | | | **Perceived Tear Intensity**  **[Robust Estimation]** | | | | |
| --- | --- | --- | --- | --- | --- | --- | --- | --- | --- | --- | --- |
| *Predictors* | *Estimates* | *std. Beta* | *CI* | *standardized CI* | *p* | *std. p* | *Estimates* | *std. Beta* | *CI* | *standardized CI* | *p* |
| (Intercept) | 1.53 | -0.82 | 1.37 – 1.70 | -0.90 – -0.74 | **<0.001** | **<0.001** | 1.45 | -0.85 | 1.28 – 1.63 | -0.94 – -0.77 | **<0.001** |
| Tear Intensity [No Tears vs. Subtle Tears] | 2.02 | 0.97 | 1.87 – 2.16 | 0.90 – 1.04 | **<0.001** | **<0.001** | 2.10 | 1.01 | 1.96 – 2.24 | 0.95 – 1.08 | **<0.001** |
| Tear Intensity [No Tears vs. Intense Tears] | 3.27 | 1.58 | 3.13 – 3.42 | 1.51 – 1.65 | **<0.001** | **<0.001** | 3.51 | 1.69 | 3.37 – 3.65 | 1.63 – 1.76 | **<0.001** |
| **Random Effects** | | | | | | | | | | | |
| σ^2^ | 1.64 | | | | | | 1.40 | | | | |
| τ_00_ | 0.45 _ID_ | | | | | | 0.38 _ID_ | | | | |
|  | 0.38 _Video_ID:Actor_ID_ | | | | | | 0.33 _Video_ID:Actor_ID_ | | | | |
|  | 0.04 _Actor_ID_ | | | | | | 0.05 _Actor_ID_ | | | | |
| ICC | 0.35 | | | | | | 0.35 | | | | |
| N | 1100 _ID_ | | | | | | 1100 _ID_ | | | | |
|  | 50 _Video_ID_ | | | | | | 50 _Video_ID_ | | | | |
|  | 10 _Actor_ID_ | | | | | | 10 _Actor_ID_ | | | | |
| Observations | 10666 | | | | | | 10666 | | | | |
| Marginal R^2^ / Conditional R^2^ | 0.424 / 0.624 | | | | | | 0.495 / 0.672 | | | | |

**Supplementary Table 3**

*Overview of Multilevel Model with Perceived Facial Expression Intensity as the Outcome Variable and Facial Expression Intensity (Natural vs. Exaggerated) as the Predictor. Random Intercepts Based on Participants and Videos Nested in Actors. Left side shows the originally registered model and the right side shows the model with robust estimation using the robustlmm package.*

|  | **Perceived Facial Expression Intensity [Registered Model]** | | | | | **Perceived Facial Expression Intensity [Robust Model]** | | | | |
| --- | --- | --- | --- | --- | --- | --- | --- | --- | --- | --- |
| *Predictors* | *Estimates* | *std. Beta* | *CI* | *standardized CI* | *p* | *Estimates* | *std. Beta* | *CI* | *standardized CI* | *p* |
| (Intercept) | 3.90 | -0.39 | 3.62 – 4.17 | -0.55 – -0.23 | **<0.001** | 4.08 | -0.29 | 3.89 – 4.27 | -0.39 – -0.18 | **<0.001** |
| Facial Expression Intensity (natural vs. exaggerated) | 1.40 | 0.80 | 1.26 – 1.53 | 0.72 – 0.87 | **<0.001** | 1.34 | 0.77 | 1.23 – 1.46 | 0.70 – 0.83 | **<0.001** |
| **Random Effects** | | | | | | | | | | |
| σ^2^ | 1.63 | | | | | 1.55 | | | | |
| τ_00_ | 0.36 _ID_ | | | | | 0.35 _ID_ | | | | |
|  | 0.49 _Video_ID:Actor_ID_ | | | | | 0.34 _Video_ID:Actor_ID_ | | | | |
|  | 0.18 _Actor_ID_ | | | | | 0.07 _Actor_ID_ | | | | |
| ICC | 0.39 | | | | | 0.33 | | | | |
| N | 1100 _ID_ | | | | | 1100 _ID_ | | | | |
|  | 50 _Video_ID_ | | | | | 50 _Video_ID_ | | | | |
|  | 10 _Actor_ID_ | | | | | 10 _Actor_ID_ | | | | |
| Observations | 10667 | | | | | 10667 | | | | |
| Marginal R^2^ / Conditional R^2^ | 0.155 / 0.481 | | | | | 0.164 / 0.439 | | | | |

**Supplementary Table 4**

*Overview of Multilevel Model with Perceived Face Touching as the Outcome Variable and Face Touching (No Face Touching vs. Face Touching) as the Predictor. Random Intercepts Based on Participants and Videos Nested in Actors. Left side shows the originally registered model and the right side shows the model with robust estimation using the robustlmm package.*

|  | **Perceived Face Touching**  **[Registered Model]** | | | | | **Perceived Face Touching**  **[Robust Model]** | | | | |
| --- | --- | --- | --- | --- | --- | --- | --- | --- | --- | --- |
| *Predictors* | *Estimates* | *std. Beta* | *CI* | *standardized CI* | *p* | *Estimates* | *std. Beta* | *CI* | *standardized CI* | *p* |
| (Intercept) | 1.18 | -0.89 | 1.06 – 1.30 | -0.94 – -0.83 | **<0.001** | 1.11 | -0.92 | 1.02 – 1.21 | -0.96 – -0.88 | **<0.001** |
| Face Touching (No vs. Face Touching) | 3.87 | 1.73 | 3.80 – 3.94 | 1.70 – 1.76 | **<0.001** | 4.10 | 1.84 | 4.05 – 4.15 | 1.81 – 1.86 | **<0.001** |
| **Random Effects** | | | | | | | | | | |
| σ^2^ | 0.95 | | | | | 0.45 | | | | |
| τ_00_ | 0.18 _ID_ | | | | | 0.07 _ID_ | | | | |
|  | 0.11 _Video_ID:Actor_ID_ | | | | | 0.06 _Video_ID:Actor_ID_ | | | | |
|  | 0.03 _Actor_ID_ | | | | | 0.02 _Actor_ID_ | | | | |
| ICC | 0.25 | | | | | 0.25 | | | | |
| N | 1100 _ID_ | | | | | 1100 _ID_ | | | | |
|  | 50 _Video_ID_ | | | | | 50 _Video_ID_ | | | | |
|  | 10 _Actor_ID_ | | | | | 10 _Actor_ID_ | | | | |
| Observations | 10666 | | | | | 10666 | | | | |
| Marginal R^2^ / Conditional R^2^ | 0.747 / 0.810 | | | | | 0.874 / 0.906 | | | | |

**Supplementary Table 5**

*Overview of Multilevel Model with Perceived Vocalizations as the Outcome Variable and Vocalizations (Quiet vs. Loud Crying) as the Predictor. Random Intercepts Based on Participants and Videos Nested in Actors. Left side shows the originally registered model and the right side shows the model with robust estimation using the robustlmm package.*

|  | **Perceived Vocalizations**  **[Registered Model]** | | | | | | **Perceived Vocalizations**  **[Robust Model]** | | | | |
| --- | --- | --- | --- | --- | --- | --- | --- | --- | --- | --- | --- |
| *Predictors* | *Estimates* | *std. Beta* | *CI* | *standardized CI* | *p* | *std. p* | *Estimates* | *std. Beta* | *CI* | *standardized CI* | *p* |
| (Intercept) | 1.88 | -0.70 | 1.71 – 2.05 | -0.79 – -0.61 | **<0.001** | **<0.001** | 1.79 | -0.75 | 1.60 – 1.98 | -0.85 – -0.64 | **<0.001** |
| Vocalizations (Quiet vs. Loud Crying) | 2.65 | 1.43 | 2.54 – 2.76 | 1.37 – 1.49 | **<0.001** | **<0.001** | 2.74 | 1.48 | 2.63 – 2.86 | 1.42 – 1.54 | **<0.001** |
| **Random Effects** | | | | | | | | | | | |
| σ^2^ | 1.01 | | | | | | 0.83 | | | | |
| τ_00_ | 0.38 _ID_ | | | | | | 0.34 _ID_ | | | | |
|  | 0.34 _Video_ID:Actor_ID_ | | | | | | 0.37 _Video_ID:Actor_ID_ | | | | |
|  | 0.06 _Actor_ID_ | | | | | | 0.07 _Actor_ID_ | | | | |
| ICC | 0.44 | | | | | | 0.48 | | | | |
| N | 1100 _ID_ | | | | | | 1100 _ID_ | | | | |
|  | 50 _Video_ID_ | | | | | | 50 _Video_ID_ | | | | |
|  | 10 _Actor_ID_ | | | | | | 10 _Actor_ID_ | | | | |
| Observations | 10666 | | | | | | 10666 | | | | |
| Marginal R^2^ / Conditional R^2^ | 0.495 / 0.716 | | | | | | 0.538 / 0.761 | | | | |

|  |  |
| --- | --- |

**Supplementary Table 6**

*Overview of Multilevel Model with Perceived Sudden Stopping as the Outcome Variable and Sudden Stopping (Gradual vs. Sudden) as the Predictor. Random Intercepts Based on Participants and Videos Nested in Actors. Note. Convergence issues in original model switching to Nelder-Mead optimization to resolve convergence issues. Left side shows the originally registered model and the right side shows the model with robust estimation using the robustlmm package.*

|  | **Perceived Sudden Stopping**  **[Registered Model]** | | | | | **Perceived Sudden Stopping**  **[Robust Model]** | | | | | |
| --- | --- | --- | --- | --- | --- | --- | --- | --- | --- | --- | --- |
| *Predictors* | *Estimates* | *std. Beta* | *CI* | *standardized CI* | *p* | *Estimates* | *std. Beta* | *CI* | *standardized CI* | *p* | *std. p* |
| (Intercept) | 1.39 | -0.87 | 1.29 – 1.49 | -0.91 – -0.84 | **<0.001** | 1.00 | -1.01 | 1.00 – 1.00 | -1.01 – -1.01 | **<0.001** | **<0.001** |
| Perceived Sudden Stopping [Gradual vs. Sudden] | 5.00 | 1.80 | 4.91 – 5.08 | 1.77 – 1.83 | **<0.001** | 6.00 | 2.16 | 6.00 – 6.00 | 2.16 – 2.16 | **<0.001** | **<0.001** |
| **Random Effects** | | | | | | | | | | | |
| σ^2^ | 1.20 | | | | | 0.00 | | | | | |
| τ_00_ | 0.08 _ID_ | | | | | 0.00 _ID_ | | | | | |
|  | 0.20 _Video_ID:Actor_ID_ | | | | | 0.00 _Video_ID:Actor_ID_ | | | | | |
|  | 0.01 _Actor_ID_ | | | | | 0.00 _Actor_ID_ | | | | | |
| ICC | 0.20 | | | | |  | | | | | |
| N | 1100 _ID_ | | | | | 1100 _ID_ | | | | | |
|  | 50 _Video_ID_ | | | | | 50 _Video_ID_ | | | | | |
|  | 10 _Actor_ID_ | | | | | 10 _Actor_ID_ | | | | | |
| Observations | 10667 | | | | | 10667 | | | | | |
| Marginal R^2^ / Conditional R^2^ | 0.807 / 0.845 | | | | | 1.000 / NA | | | | | |

**3.2 Study 1 – Radar Plots**

**Supplementary Figure 6**

*Radar Plots of Perception Match for Each of the 50 Videos (Video IDs from 1 to 50) for the 10 Different Actors. Radar Plots Define the Match Percentage Between Intended Manipulation and Perception Rating for Tear Intensity (TI), Facial Expression Intensity (FE), Face Touching (FT), Vocalizations (VO), and Sudden Stopping (SS)*


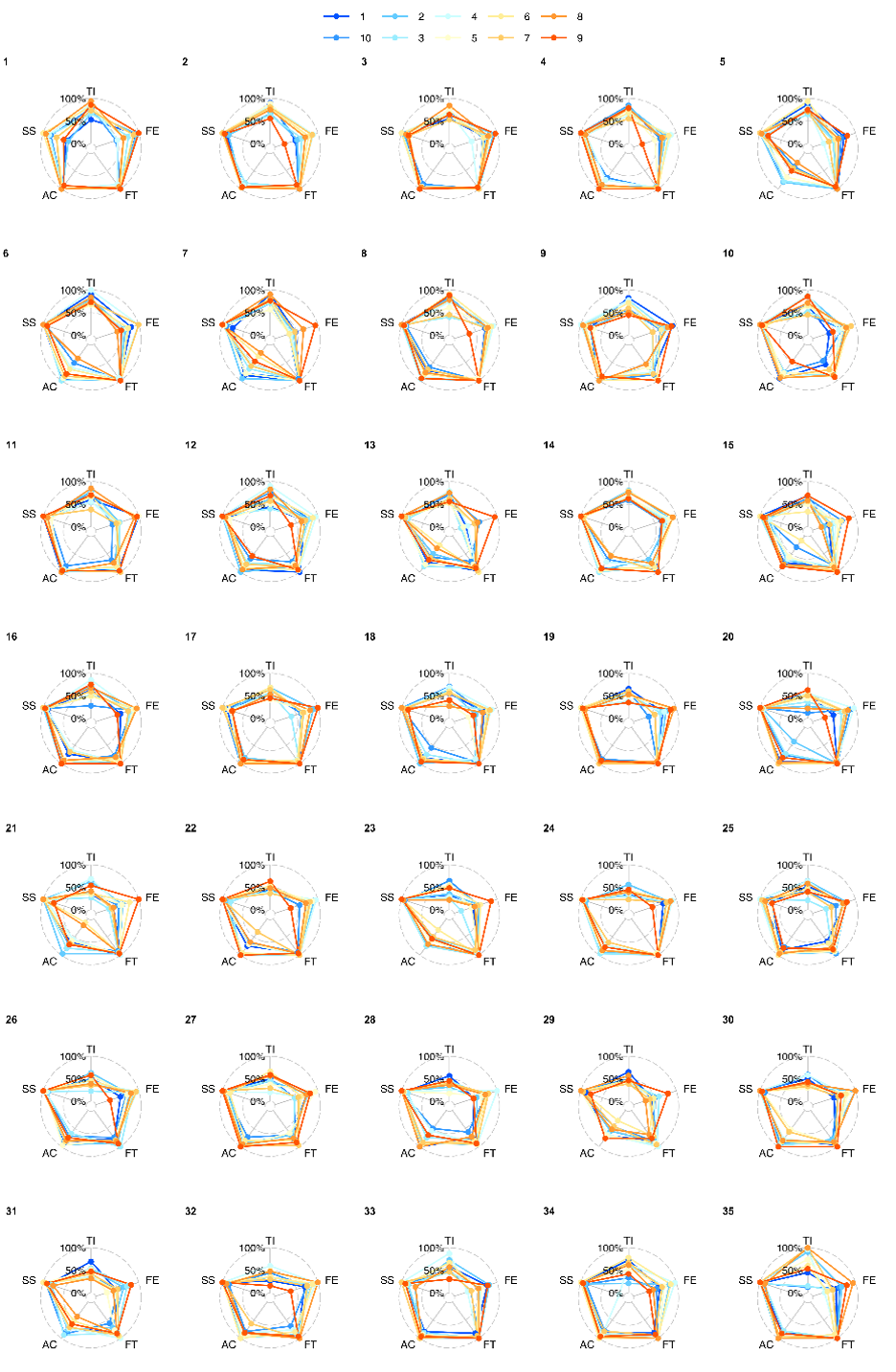


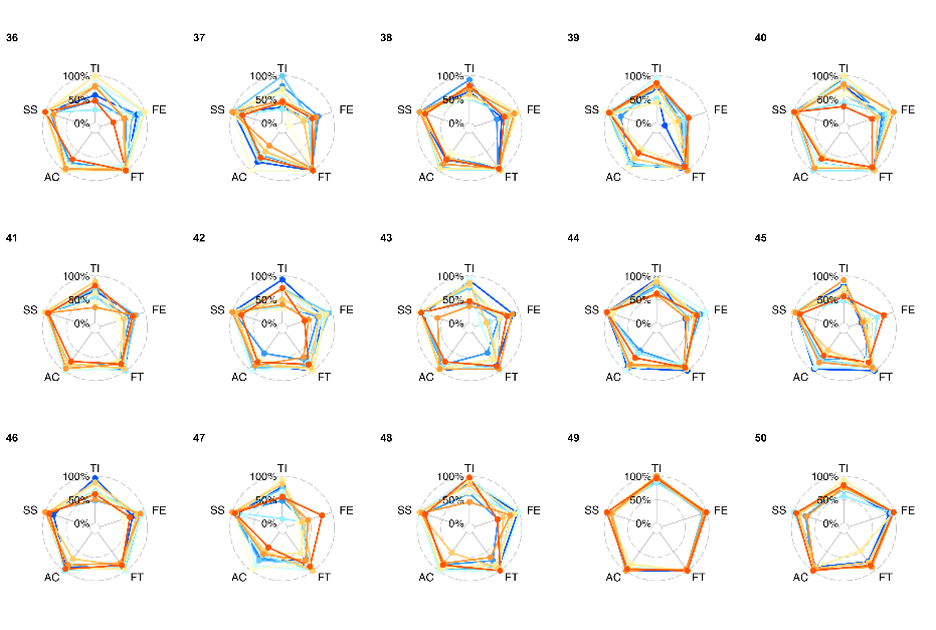


**3.3 Study 1 - Inter-Rater Reliability**

We explored inter-rater reliability across videos and measures for Study 1. None of the analyses were pre-registered.

First, we investigated inter-rater reliability by inspecting intraclass correlation coefficients (ICC) for each of the five measured variables across all videos. For this, we fitted five separate multilevel intercept-only models for each variable and added participant and video id as random intercepts. We also explored models with video ids nested in actors but observed that considering actors as random intercepts added only little above models without them. For tear intensity, we observed high consistency in how stimulus were perceived (ICC = 0.52) and less variance explained by differences in raters (ICC = 0.10). For vocalizations, we again observed high consistency for stimuli (ICC = 0.61) and little variance explained by differences in participants (ICC = 0.11). For sudden stopping these effects were even more pronounced with a high consistency for stimuli (ICC = 0.84) and little variation attributed to participants (ICC = 0.01). Similar results were obtained for face touching (stimuli: ICC = 0.78; participants: ICC = 0.04). We observed the lowest consistency for stimuli for facial expression ratings (ICC = 0.36), while similar variance was explained by raters (ICC = 0.12). These findings provide a first picture of inter-rater reliability for each variable across videos.

Second, we focused on individual videos and consistency and agreements in ratings of the five measures (tear intensity, facial expression, face touch, sudden stopping, and vocalizations). Successful videos should show high consistency of ratings across participants and measures. For example, videos attempting to manipulate high tear intensity but an absence of the remaining factors should receive consistently high ratings on tear intensity but low ratings on the remaining measures. We calculated intraclass correlation coefficient for each video across raters and variables (using a one-way model due to the fact that raters are randomly chosen for each video). Importantly, ICC or other inter-rater reliability measures like Cohen’s kappa are known to penalize high agreement and low variation in ratings (Feinstein & Cicchetti, 1990). A situation in which all raters agree on the same measure and resulting in zero variance would paradoxically result in a low ICC. To provide additional information for these potential occurrences we calculated two additional measures of inter-rater reliability or agreement. First, we included Gwet’s Agreement Coefficient (AC2 for ordinal weighting) known to account for situations of high agreement (Gwet, 2008). Second, we also averaged standard deviations across the five variables for each video by taking the root mean square. A low value indicates low variation of ratings across the five variables. An overview of all inter-rater reliability measures is provided in Supplementary Table 7 and Supplementary Figure 7. Based on conventions we explored the performance of individual videos and flagged videos with poor inter-rater reliabilities with an ICC lower than 0.50 OR an AC2 lower than 0.50 OR an average SD above 1. This flagged 22 out of 500 videos with poor reliability (Supplementary Table 7) and only 4.4% of videos. Inconsistencies were mostly present with videos including some form of tears and were more prevalent for Actor ID 9. Nevertheless, by far the majority of videos exhibited good to high inter-rater reliability.

**Supplementary Table 7**

*Reliabilities Including Intraclass Correlation Coefficient (ICC), Gwet’s Agreement Coefficient (AC2), and Root Mean Squared Standard Deviation (SD) for Each Individual Video Across the Five Measurements in Study 1. Red Rows Highlight Videos with Poor Reliability*

| Actor ID | Video ID | ICC | | | Gwet’s AC_2_ | | | RMS SD | n_raters |
| --- | --- | --- | --- | --- | --- | --- | --- | --- | --- |
|  |  | Est. | 95% CI | | Est. | 95% CI | |  |  |
| 1 | 1 | 0.30 | 0.10 | 0.80 | 0.77 | 0.32 | 1.00 | 1.30 | 17 |
| 1 | 2 | 0.81 | 0.59 | 0.97 | 0.79 | 0.33 | 1.00 | 1.15 | 21 |
| 1 | 3 | 0.47 | 0.21 | 0.89 | 0.76 | 0.37 | 1.00 | 1.03 | 18 |
| 1 | 4 | 0.79 | 0.56 | 0.97 | 0.90 | 0.72 | 1.00 | 0.80 | 16 |
| 1 | 5 | 0.86 | 0.66 | 0.98 | 0.77 | 0.36 | 1.00 | 0.98 | 15 |
| 1 | 6 | 0.86 | 0.68 | 0.98 | 0.80 | 0.54 | 1.00 | 1.04 | 23 |
| 1 | 7 | 0.66 | 0.39 | 0.94 | 0.74 | 0.36 | 1.00 | 1.28 | 21 |
| 1 | 8 | 0.82 | 0.60 | 0.97 | 0.85 | 0.46 | 1.00 | 1.00 | 18 |
| 1 | 9 | 0.62 | 0.35 | 0.93 | 0.57 | -0.12 | 1.00 | 1.47 | 23 |
| 1 | 10 | 0.75 | 0.49 | 0.96 | 0.63 | 0.28 | 0.98 | 1.19 | 18 |
| 1 | 11 | 0.70 | 0.43 | 0.95 | 0.79 | 0.54 | 1.00 | 0.98 | 15 |
| 1 | 12 | 0.71 | 0.44 | 0.95 | 0.67 | 0.32 | 1.00 | 1.21 | 16 |
| 1 | 13 | 0.66 | 0.40 | 0.94 | 0.57 | 0.46 | 0.69 | 1.31 | 29 |
| 1 | 14 | 0.78 | 0.54 | 0.97 | 0.72 | 0.43 | 1.00 | 1.05 | 21 |
| 1 | 15 | 0.68 | 0.42 | 0.95 | 0.63 | 0.16 | 1.00 | 1.28 | 32 |
| 1 | 16 | 0.54 | 0.27 | 0.91 | 0.47 | 0.13 | 0.80 | 1.51 | 19 |
| 1 | 17 | 0.63 | 0.35 | 0.94 | 0.54 | 0.03 | 1.00 | 1.42 | 19 |
| 1 | 18 | 0.82 | 0.60 | 0.97 | 0.72 | 0.31 | 1.00 | 1.05 | 20 |
| 1 | 19 | 0.52 | 0.26 | 0.90 | 0.71 | 0.31 | 1.00 | 1.22 | 23 |
| 1 | 20 | 0.62 | 0.35 | 0.93 | 0.80 | 0.39 | 1.00 | 1.10 | 29 |
| 1 | 21 | 0.79 | 0.55 | 0.97 | 0.70 | 0.26 | 1.00 | 1.15 | 17 |
| 1 | 22 | 0.63 | 0.35 | 0.94 | 0.50 | 0.11 | 0.90 | 1.46 | 16 |
| 1 | 23 | 0.59 | 0.31 | 0.92 | 0.66 | 0.19 | 1.00 | 1.27 | 18 |
| 1 | 24 | 0.73 | 0.47 | 0.96 | 0.74 | 0.19 | 1.00 | 1.21 | 23 |
| 1 | 25 | 0.25 | 0.08 | 0.76 | 0.29 | -0.13 | 0.71 | 1.70 | 21 |
| 1 | 26 | 0.56 | 0.29 | 0.91 | 0.56 | 0.38 | 0.74 | 1.30 | 26 |
| 1 | 27 | 0.48 | 0.23 | 0.89 | 0.56 | 0.35 | 0.77 | 1.36 | 28 |
| 1 | 28 | 0.59 | 0.32 | 0.92 | 0.63 | 0.15 | 1.00 | 1.29 | 30 |
| 1 | 29 | 0.31 | 0.12 | 0.80 | 0.51 | 0.17 | 0.86 | 1.45 | 26 |
| 1 | 30 | 0.46 | 0.20 | 0.88 | 0.61 | 0.29 | 0.94 | 1.29 | 20 |
| 1 | 31 | 0.45 | 0.20 | 0.88 | 0.40 | 0.05 | 0.74 | 1.56 | 23 |
| 1 | 32 | 0.61 | 0.34 | 0.93 | 0.61 | 0.18 | 1.00 | 1.33 | 26 |
| 1 | 33 | 0.55 | 0.28 | 0.91 | 0.42 | -0.06 | 0.89 | 1.58 | 22 |
| 1 | 34 | 0.71 | 0.45 | 0.95 | 0.57 | 0.18 | 0.96 | 1.34 | 26 |
| 1 | 35 | 0.60 | 0.32 | 0.93 | 0.68 | 0.12 | 1.00 | 1.28 | 20 |
| 1 | 36 | 0.73 | 0.47 | 0.96 | 0.78 | 0.34 | 1.00 | 1.11 | 17 |
| 1 | 37 | 0.66 | 0.39 | 0.94 | 0.52 | -0.01 | 1.00 | 1.43 | 27 |
| 1 | 38 | 0.64 | 0.36 | 0.94 | 0.53 | 0.05 | 1.00 | 1.44 | 18 |
| 1 | 39 | 0.77 | 0.54 | 0.97 | 0.70 | 0.24 | 1.00 | 1.15 | 27 |
| 1 | 40 | 0.70 | 0.44 | 0.95 | 0.62 | 0.17 | 1.00 | 1.41 | 24 |
| 1 | 41 | 0.71 | 0.45 | 0.95 | 0.63 | 0.36 | 0.90 | 1.16 | 22 |
| 1 | 42 | 0.77 | 0.52 | 0.97 | 0.75 | 0.49 | 1.00 | 1.00 | 13 |
| 1 | 43 | 0.71 | 0.45 | 0.95 | 0.66 | 0.30 | 1.00 | 1.20 | 22 |
| 1 | 44 | 0.80 | 0.57 | 0.97 | 0.75 | 0.54 | 0.97 | 0.95 | 15 |
| 1 | 45 | 0.22 | 0.07 | 0.72 | 0.71 | 0.52 | 0.90 | 1.22 | 25 |
| 1 | 46 | 0.06 | -0.01 | 0.46 | 0.61 | 0.27 | 0.95 | 1.41 | 22 |
| 1 | 47 | 0.51 | 0.24 | 0.90 | 0.55 | 0.42 | 0.68 | 1.38 | 18 |
| 1 | 48 | 0.78 | 0.54 | 0.97 | 0.74 | 0.58 | 0.92 | 1.05 | 30 |
| 1 | 49 | 0.04 | -0.02 | 0.45 | 0.94 | 0.83 | 1.00 | 0.46 | 17 |
| 1 | 50 | 0.53 | 0.27 | 0.91 | 0.80 | 0.55 | 1.00 | 1.19 | 21 |
| 2 | 1 | 0.21 | 0.06 | 0.72 | 0.74 | 0.36 | 1.00 | 1.34 | 24 |
| 2 | 2 | 0.77 | 0.53 | 0.97 | 0.72 | 0.21 | 1.00 | 1.23 | 21 |
| 2 | 3 | 0.27 | 0.09 | 0.77 | 0.80 | 0.54 | 1.00 | 0.99 | 24 |
| 2 | 4 | 0.81 | 0.58 | 0.97 | 0.89 | 0.69 | 1.00 | 0.83 | 16 |
| 2 | 5 | 0.82 | 0.60 | 0.97 | 0.74 | 0.29 | 1.00 | 1.08 | 16 |
| 2 | 6 | 0.84 | 0.64 | 0.98 | 0.75 | 0.41 | 1.00 | 1.10 | 29 |
| 2 | 7 | 0.71 | 0.44 | 0.95 | 0.70 | 0.27 | 1.00 | 1.33 | 18 |
| 2 | 8 | 0.81 | 0.60 | 0.97 | 0.81 | 0.39 | 1.00 | 1.19 | 21 |
| 2 | 9 | 0.54 | 0.27 | 0.91 | 0.46 | -0.26 | 1.00 | 1.61 | 19 |
| 2 | 10 | 0.75 | 0.50 | 0.96 | 0.64 | 0.24 | 1.00 | 1.43 | 28 |
| 2 | 11 | 0.65 | 0.38 | 0.94 | 0.70 | 0.24 | 1.00 | 1.25 | 27 |
| 2 | 12 | 0.75 | 0.49 | 0.96 | 0.71 | 0.25 | 1.00 | 1.27 | 18 |
| 2 | 13 | 0.64 | 0.36 | 0.94 | 0.50 | 0.05 | 0.95 | 1.40 | 20 |
| 2 | 14 | 0.74 | 0.48 | 0.96 | 0.64 | 0.29 | 1.00 | 1.22 | 20 |
| 2 | 15 | 0.56 | 0.29 | 0.92 | 0.44 | 0.24 | 0.64 | 1.53 | 22 |
| 2 | 16 | 0.80 | 0.58 | 0.97 | 0.70 | 0.44 | 0.96 | 1.16 | 17 |
| 2 | 17 | 0.61 | 0.33 | 0.93 | 0.57 | -0.03 | 1.00 | 1.41 | 19 |
| 2 | 18 | 0.73 | 0.46 | 0.96 | 0.62 | 0.10 | 1.00 | 1.35 | 16 |
| 2 | 19 | 0.44 | 0.19 | 0.88 | 0.86 | 0.53 | 1.00 | 1.09 | 17 |
| 2 | 20 | 0.73 | 0.46 | 0.96 | 0.67 | 0.14 | 1.00 | 1.25 | 15 |
| 2 | 21 | 0.80 | 0.57 | 0.97 | 0.69 | 0.31 | 1.00 | 1.10 | 24 |
| 2 | 22 | 0.71 | 0.46 | 0.95 | 0.60 | 0.29 | 0.91 | 1.40 | 24 |
| 2 | 23 | 0.64 | 0.36 | 0.94 | 0.69 | 0.16 | 1.00 | 1.22 | 17 |
| 2 | 24 | 0.72 | 0.47 | 0.96 | 0.59 | 0.22 | 0.97 | 1.33 | 25 |
| 2 | 25 | 0.75 | 0.50 | 0.96 | 0.63 | 0.32 | 0.94 | 1.19 | 25 |
| 2 | 26 | 0.62 | 0.34 | 0.93 | 0.59 | 0.42 | 0.76 | 1.25 | 19 |
| 2 | 27 | 0.36 | 0.14 | 0.83 | 0.50 | 0.19 | 0.82 | 1.50 | 22 |
| 2 | 28 | 0.70 | 0.43 | 0.95 | 0.66 | 0.33 | 1.00 | 1.17 | 19 |
| 2 | 29 | 0.31 | 0.12 | 0.79 | 0.56 | 0.44 | 0.69 | 1.36 | 35 |
| 2 | 30 | 0.54 | 0.27 | 0.91 | 0.68 | 0.13 | 1.00 | 1.33 | 20 |
| 2 | 31 | 0.37 | 0.14 | 0.84 | 0.44 | 0.19 | 0.69 | 1.29 | 17 |
| 2 | 32 | 0.68 | 0.41 | 0.95 | 0.63 | 0.52 | 0.75 | 1.25 | 20 |
| 2 | 33 | 0.63 | 0.36 | 0.94 | 0.61 | -0.19 | 1.00 | 1.41 | 22 |
| 2 | 34 | 0.74 | 0.49 | 0.96 | 0.69 | 0.23 | 1.00 | 1.45 | 19 |
| 2 | 35 | 0.78 | 0.54 | 0.97 | 0.78 | 0.30 | 1.00 | 1.09 | 22 |
| 2 | 36 | 0.77 | 0.52 | 0.97 | 0.71 | 0.24 | 1.00 | 1.19 | 14 |
| 2 | 37 | 0.88 | 0.70 | 0.98 | 0.83 | 0.58 | 1.00 | 0.82 | 12 |
| 2 | 38 | 0.75 | 0.51 | 0.96 | 0.69 | 0.49 | 0.90 | 1.15 | 27 |
| 2 | 39 | 0.76 | 0.52 | 0.96 | 0.73 | 0.22 | 1.00 | 1.15 | 28 |
| 2 | 40 | 0.79 | 0.55 | 0.97 | 0.72 | 0.10 | 1.00 | 1.23 | 25 |
| 2 | 41 | 0.69 | 0.43 | 0.95 | 0.61 | 0.32 | 0.90 | 1.23 | 30 |
| 2 | 42 | 0.66 | 0.37 | 0.94 | 0.62 | 0.37 | 0.87 | 1.24 | 15 |
| 2 | 43 | 0.68 | 0.40 | 0.95 | 0.66 | 0.40 | 0.93 | 1.13 | 16 |
| 2 | 44 | 0.65 | 0.38 | 0.94 | 0.56 | 0.15 | 0.98 | 1.32 | 23 |
| 2 | 45 | 0.22 | 0.07 | 0.73 | 0.54 | 0.30 | 0.79 | 1.42 | 24 |
| 2 | 46 | 0.33 | 0.12 | 0.81 | 0.67 | 0.26 | 1.00 | 1.27 | 23 |
| 2 | 47 | 0.61 | 0.34 | 0.93 | 0.53 | 0.25 | 0.81 | 1.35 | 22 |
| 2 | 48 | 0.80 | 0.58 | 0.97 | 0.75 | 0.51 | 0.98 | 1.08 | 22 |
| 2 | 49 | 0.20 | 0.06 | 0.71 | 0.92 | 0.79 | 1.00 | 0.98 | 25 |
| 2 | 50 | 0.66 | 0.38 | 0.94 | 0.76 | 0.46 | 1.00 | 1.03 | 17 |
| 3 | 1 | 0.62 | 0.34 | 0.93 | 0.67 | 0.11 | 1.00 | 1.34 | 21 |
| 3 | 2 | 0.85 | 0.67 | 0.98 | 0.78 | 0.50 | 1.00 | 0.99 | 24 |
| 3 | 3 | 0.52 | 0.26 | 0.90 | 0.85 | 0.53 | 1.00 | 0.86 | 20 |
| 3 | 4 | 0.67 | 0.40 | 0.95 | 0.83 | 0.46 | 1.00 | 1.08 | 20 |
| 3 | 5 | 0.73 | 0.48 | 0.96 | 0.62 | 0.16 | 1.00 | 1.27 | 23 |
| 3 | 6 | 0.83 | 0.63 | 0.98 | 0.74 | 0.53 | 0.95 | 1.10 | 28 |
| 3 | 7 | 0.80 | 0.57 | 0.97 | 0.86 | 0.52 | 1.00 | 0.92 | 28 |
| 3 | 8 | 0.86 | 0.68 | 0.98 | 0.83 | 0.48 | 1.00 | 0.93 | 20 |
| 3 | 9 | 0.76 | 0.51 | 0.96 | 0.68 | 0.15 | 1.00 | 1.26 | 22 |
| 3 | 10 | 0.74 | 0.50 | 0.96 | 0.64 | 0.34 | 0.93 | 1.18 | 31 |
| 3 | 11 | 0.73 | 0.47 | 0.96 | 0.74 | 0.37 | 1.00 | 1.15 | 20 |
| 3 | 12 | 0.68 | 0.41 | 0.95 | 0.66 | 0.32 | 1.00 | 1.18 | 20 |
| 3 | 13 | 0.53 | 0.26 | 0.91 | 0.51 | 0.22 | 0.79 | 1.46 | 19 |
| 3 | 14 | 0.66 | 0.39 | 0.94 | 0.61 | 0.20 | 1.00 | 1.31 | 26 |
| 3 | 15 | 0.72 | 0.46 | 0.96 | 0.66 | 0.26 | 1.00 | 1.17 | 22 |
| 3 | 16 | 0.74 | 0.49 | 0.96 | 0.61 | 0.41 | 0.82 | 1.29 | 21 |
| 3 | 17 | 0.55 | 0.28 | 0.91 | 0.50 | -0.24 | 1.00 | 1.64 | 21 |
| 3 | 18 | 0.83 | 0.62 | 0.98 | 0.74 | 0.33 | 1.00 | 1.12 | 16 |
| 3 | 19 | 0.51 | 0.25 | 0.90 | 0.76 | 0.25 | 1.00 | 1.26 | 22 |
| 3 | 20 | 0.55 | 0.28 | 0.91 | 0.70 | 0.12 | 1.00 | 1.34 | 22 |
| 3 | 21 | 0.73 | 0.48 | 0.96 | 0.63 | 0.34 | 0.91 | 1.32 | 26 |
| 3 | 22 | 0.69 | 0.41 | 0.95 | 0.55 | -0.02 | 1.00 | 1.50 | 16 |
| 3 | 23 | 0.65 | 0.36 | 0.94 | 0.71 | 0.21 | 1.00 | 1.31 | 12 |
| 3 | 24 | 0.77 | 0.54 | 0.97 | 0.70 | 0.07 | 1.00 | 1.24 | 23 |
| 3 | 25 | 0.75 | 0.50 | 0.96 | 0.61 | 0.22 | 1.00 | 1.32 | 19 |
| 3 | 26 | 0.64 | 0.37 | 0.94 | 0.59 | 0.05 | 1.00 | 1.31 | 22 |
| 3 | 27 | 0.56 | 0.29 | 0.91 | 0.56 | 0.18 | 0.95 | 1.38 | 26 |
| 3 | 28 | 0.50 | 0.25 | 0.90 | 0.49 | 0.28 | 0.69 | 1.44 | 28 |
| 3 | 29 | 0.68 | 0.41 | 0.95 | 0.62 | 0.19 | 1.00 | 1.21 | 20 |
| 3 | 30 | 0.42 | 0.18 | 0.86 | 0.59 | 0.07 | 1.00 | 1.42 | 22 |
| 3 | 31 | 0.39 | 0.16 | 0.85 | 0.50 | 0.19 | 0.82 | 1.45 | 22 |
| 3 | 32 | 0.60 | 0.34 | 0.93 | 0.52 | 0.25 | 0.80 | 1.44 | 28 |
| 3 | 33 | 0.47 | 0.20 | 0.89 | 0.41 | -0.42 | 1.00 | 1.86 | 13 |
| 3 | 34 | 0.77 | 0.54 | 0.97 | 0.66 | 0.35 | 0.97 | 1.14 | 24 |
| 3 | 35 | 0.53 | 0.26 | 0.91 | 0.71 | 0.28 | 1.00 | 1.27 | 20 |
| 3 | 36 | 0.66 | 0.38 | 0.94 | 0.66 | 0.10 | 1.00 | 1.34 | 18 |
| 3 | 37 | 0.49 | 0.23 | 0.89 | 0.38 | -0.09 | 0.84 | 1.67 | 24 |
| 3 | 38 | 0.69 | 0.43 | 0.95 | 0.59 | 0.12 | 1.00 | 1.42 | 31 |
| 3 | 39 | 0.59 | 0.32 | 0.92 | 0.63 | 0.16 | 1.00 | 1.39 | 27 |
| 3 | 40 | 0.78 | 0.53 | 0.97 | 0.73 | 0.21 | 1.00 | 1.16 | 14 |
| 3 | 41 | 0.64 | 0.36 | 0.94 | 0.57 | 0.08 | 1.00 | 1.30 | 18 |
| 3 | 42 | 0.65 | 0.37 | 0.94 | 0.61 | 0.09 | 1.00 | 1.25 | 20 |
| 3 | 43 | 0.64 | 0.34 | 0.94 | 0.68 | 0.49 | 0.87 | 1.16 | 12 |
| 3 | 44 | 0.73 | 0.47 | 0.96 | 0.66 | 0.37 | 0.96 | 1.15 | 22 |
| 3 | 45 | 0.22 | 0.06 | 0.73 | 0.50 | 0.27 | 0.73 | 1.48 | 19 |
| 3 | 46 | 0.11 | 0.01 | 0.56 | 0.64 | 0.41 | 0.87 | 1.38 | 25 |
| 3 | 47 | 0.64 | 0.37 | 0.94 | 0.54 | 0.10 | 0.99 | 1.34 | 22 |
| 3 | 48 | 0.67 | 0.40 | 0.95 | 0.63 | 0.32 | 0.94 | 1.25 | 24 |
| 3 | 49 | 0.12 | 0.00 | 0.63 | 0.90 | 0.70 | 1.00 | 0.57 | 14 |
| 3 | 50 | 0.58 | 0.30 | 0.92 | 0.76 | 0.40 | 1.00 | 1.27 | 19 |
| 4 | 1 | 0.66 | 0.38 | 0.94 | 0.66 | 0.12 | 1.00 | 1.52 | 16 |
| 4 | 2 | 0.87 | 0.68 | 0.98 | 0.81 | 0.61 | 1.00 | 1.09 | 19 |
| 4 | 3 | 0.68 | 0.42 | 0.95 | 0.85 | 0.64 | 1.00 | 1.00 | 27 |
| 4 | 4 | 0.74 | 0.49 | 0.96 | 0.76 | 0.45 | 1.00 | 1.24 | 24 |
| 4 | 5 | 0.84 | 0.64 | 0.98 | 0.75 | 0.40 | 1.00 | 1.05 | 23 |
| 4 | 6 | 0.86 | 0.68 | 0.98 | 0.81 | 0.52 | 1.00 | 1.06 | 14 |
| 4 | 7 | 0.69 | 0.42 | 0.95 | 0.79 | 0.33 | 1.00 | 1.09 | 16 |
| 4 | 8 | 0.86 | 0.68 | 0.98 | 0.84 | 0.60 | 1.00 | 0.92 | 23 |
| 4 | 9 | 0.75 | 0.51 | 0.96 | 0.65 | 0.22 | 1.00 | 1.29 | 22 |
| 4 | 10 | 0.80 | 0.57 | 0.97 | 0.68 | 0.15 | 1.00 | 1.20 | 24 |
| 4 | 11 | 0.66 | 0.40 | 0.94 | 0.64 | 0.23 | 1.00 | 1.23 | 27 |
| 4 | 12 | 0.83 | 0.63 | 0.98 | 0.77 | 0.50 | 1.00 | 1.16 | 21 |
| 4 | 13 | 0.63 | 0.36 | 0.93 | 0.51 | 0.21 | 0.80 | 1.50 | 29 |
| 4 | 14 | 0.72 | 0.45 | 0.96 | 0.64 | 0.28 | 1.00 | 1.22 | 18 |
| 4 | 15 | 0.53 | 0.27 | 0.90 | 0.52 | 0.41 | 0.64 | 1.38 | 30 |
| 4 | 16 | 0.77 | 0.54 | 0.97 | 0.66 | 0.19 | 1.00 | 1.27 | 27 |
| 4 | 17 | 0.64 | 0.37 | 0.94 | 0.57 | 0.07 | 1.00 | 1.42 | 19 |
| 4 | 18 | 0.70 | 0.43 | 0.95 | 0.57 | 0.26 | 0.88 | 1.40 | 16 |
| 4 | 19 | 0.52 | 0.25 | 0.90 | 0.76 | 0.22 | 1.00 | 1.24 | 18 |
| 4 | 20 | 0.84 | 0.64 | 0.98 | 0.80 | 0.55 | 1.00 | 0.98 | 27 |
| 4 | 21 | 0.73 | 0.46 | 0.96 | 0.64 | 0.33 | 0.96 | 1.18 | 16 |
| 4 | 22 | 0.81 | 0.59 | 0.97 | 0.73 | 0.44 | 1.00 | 1.06 | 23 |
| 4 | 23 | 0.45 | 0.20 | 0.88 | 0.52 | -0.07 | 1.00 | 1.57 | 21 |
| 4 | 24 | 0.78 | 0.54 | 0.97 | 0.72 | 0.32 | 1.00 | 1.14 | 22 |
| 4 | 25 | 0.77 | 0.52 | 0.97 | 0.66 | 0.31 | 1.00 | 1.10 | 19 |
| 4 | 26 | 0.60 | 0.33 | 0.93 | 0.57 | 0.14 | 1.00 | 1.42 | 24 |
| 4 | 27 | 0.69 | 0.42 | 0.95 | 0.59 | 0.49 | 0.70 | 1.24 | 25 |
| 4 | 28 | 0.70 | 0.43 | 0.95 | 0.63 | 0.38 | 0.89 | 1.26 | 16 |
| 4 | 29 | 0.52 | 0.26 | 0.90 | 0.64 | 0.25 | 1.00 | 1.24 | 21 |
| 4 | 30 | 0.54 | 0.28 | 0.91 | 0.57 | 0.02 | 1.00 | 1.37 | 24 |
| 4 | 31 | 0.54 | 0.24 | 0.91 | 0.45 | 0.01 | 0.90 | 1.53 | 11 |
| 4 | 32 | 0.67 | 0.41 | 0.94 | 0.61 | 0.38 | 0.85 | 1.22 | 30 |
| 4 | 33 | 0.81 | 0.59 | 0.97 | 0.71 | 0.44 | 0.97 | 1.12 | 16 |
| 4 | 34 | 0.71 | 0.45 | 0.95 | 0.63 | 0.21 | 1.00 | 1.38 | 28 |
| 4 | 35 | 0.85 | 0.65 | 0.98 | 0.80 | 0.44 | 1.00 | 0.97 | 25 |
| 4 | 36 | 0.85 | 0.65 | 0.98 | 0.77 | 0.64 | 0.90 | 1.09 | 22 |
| 4 | 37 | 0.71 | 0.45 | 0.95 | 0.61 | 0.28 | 0.94 | 1.23 | 26 |
| 4 | 38 | 0.81 | 0.59 | 0.97 | 0.74 | 0.44 | 1.00 | 1.07 | 24 |
| 4 | 39 | 0.80 | 0.58 | 0.97 | 0.73 | 0.28 | 1.00 | 1.14 | 29 |
| 4 | 40 | 0.84 | 0.64 | 0.98 | 0.75 | 0.38 | 1.00 | 1.13 | 21 |
| 4 | 41 | 0.75 | 0.50 | 0.96 | 0.69 | 0.42 | 0.97 | 1.07 | 14 |
| 4 | 42 | 0.75 | 0.49 | 0.96 | 0.67 | 0.33 | 1.00 | 1.19 | 21 |
| 4 | 43 | 0.73 | 0.45 | 0.96 | 0.69 | 0.45 | 0.92 | 1.09 | 14 |
| 4 | 44 | 0.71 | 0.41 | 0.96 | 0.57 | 0.11 | 1.00 | 1.49 | 9 |
| 4 | 45 | 0.32 | 0.12 | 0.81 | 0.69 | 0.54 | 0.85 | 1.00 | 25 |
| 4 | 46 | 0.34 | 0.12 | 0.82 | 0.70 | 0.28 | 1.00 | 1.22 | 19 |
| 4 | 47 | 0.33 | 0.11 | 0.82 | 0.45 | -0.14 | 1.00 | 1.54 | 15 |
| 4 | 48 | 0.89 | 0.73 | 0.99 | 0.80 | 0.71 | 0.89 | 0.77 | 18 |
| 4 | 49 | 0.00 | -0.03 | 0.27 | 0.88 | 0.73 | 1.00 | 0.62 | 20 |
| 4 | 50 | 0.75 | 0.50 | 0.96 | 0.89 | 0.64 | 1.00 | 0.94 | 18 |
| 5 | 1 | 0.51 | 0.25 | 0.90 | 0.75 | 0.23 | 1.00 | 1.32 | 22 |
| 5 | 2 | 0.89 | 0.73 | 0.99 | 0.86 | 0.58 | 1.00 | 0.95 | 18 |
| 5 | 3 | 0.38 | 0.16 | 0.84 | 0.84 | 0.50 | 1.00 | 1.17 | 28 |
| 5 | 4 | 0.60 | 0.32 | 0.93 | 0.85 | 0.61 | 1.00 | 1.10 | 20 |
| 5 | 5 | 0.85 | 0.65 | 0.98 | 0.78 | 0.36 | 1.00 | 0.99 | 28 |
| 5 | 6 | 0.83 | 0.62 | 0.98 | 0.75 | 0.35 | 1.00 | 1.10 | 15 |
| 5 | 7 | 0.72 | 0.47 | 0.96 | 0.78 | 0.33 | 1.00 | 1.16 | 21 |
| 5 | 8 | 0.81 | 0.58 | 0.97 | 0.82 | 0.58 | 1.00 | 0.95 | 18 |
| 5 | 9 | 0.49 | 0.23 | 0.89 | 0.56 | -0.03 | 1.00 | 1.42 | 27 |
| 5 | 10 | 0.57 | 0.30 | 0.92 | 0.42 | 0.15 | 0.68 | 1.58 | 25 |
| 5 | 11 | 0.65 | 0.37 | 0.94 | 0.82 | 0.54 | 1.00 | 1.00 | 22 |
| 5 | 12 | 0.54 | 0.26 | 0.91 | 0.50 | 0.22 | 0.79 | 1.46 | 18 |
| 5 | 13 | 0.66 | 0.39 | 0.94 | 0.62 | 0.16 | 1.00 | 1.28 | 28 |
| 5 | 14 | 0.63 | 0.36 | 0.94 | 0.64 | 0.14 | 1.00 | 1.31 | 25 |
| 5 | 15 | 0.62 | 0.35 | 0.93 | 0.57 | 0.28 | 0.86 | 1.33 | 24 |
| 5 | 16 | 0.74 | 0.49 | 0.96 | 0.63 | 0.31 | 0.95 | 1.24 | 27 |
| 5 | 17 | 0.56 | 0.28 | 0.92 | 0.53 | -0.09 | 1.00 | 1.57 | 17 |
| 5 | 18 | 0.75 | 0.48 | 0.96 | 0.69 | 0.23 | 1.00 | 1.24 | 12 |
| 5 | 19 | 0.44 | 0.19 | 0.87 | 0.71 | 0.36 | 1.00 | 1.31 | 18 |
| 5 | 20 | 0.71 | 0.45 | 0.95 | 0.84 | 0.52 | 1.00 | 1.04 | 23 |
| 5 | 21 | 0.71 | 0.45 | 0.95 | 0.59 | 0.06 | 1.00 | 1.29 | 23 |
| 5 | 22 | 0.71 | 0.45 | 0.95 | 0.61 | 0.08 | 1.00 | 1.30 | 23 |
| 5 | 23 | 0.66 | 0.39 | 0.94 | 0.72 | 0.15 | 1.00 | 1.16 | 20 |
| 5 | 24 | 0.72 | 0.46 | 0.96 | 0.63 | 0.10 | 1.00 | 1.36 | 18 |
| 5 | 25 | 0.47 | 0.21 | 0.89 | 0.35 | -0.21 | 0.92 | 1.62 | 20 |
| 5 | 26 | 0.72 | 0.46 | 0.96 | 0.60 | 0.31 | 0.88 | 1.24 | 22 |
| 5 | 27 | 0.42 | 0.17 | 0.87 | 0.57 | 0.07 | 1.00 | 1.23 | 16 |
| 5 | 28 | 0.62 | 0.34 | 0.93 | 0.62 | 0.37 | 0.86 | 1.31 | 17 |
| 5 | 29 | 0.46 | 0.21 | 0.88 | 0.52 | -0.11 | 1.00 | 1.43 | 20 |
| 5 | 30 | 0.35 | 0.12 | 0.83 | 0.65 | 0.37 | 0.93 | 1.24 | 17 |
| 5 | 31 | 0.55 | 0.28 | 0.91 | 0.63 | 0.38 | 0.88 | 1.28 | 27 |
| 5 | 32 | 0.62 | 0.35 | 0.93 | 0.57 | 0.16 | 0.99 | 1.33 | 28 |
| 5 | 33 | 0.71 | 0.44 | 0.95 | 0.59 | 0.08 | 1.00 | 1.33 | 19 |
| 5 | 34 | 0.75 | 0.51 | 0.96 | 0.63 | 0.12 | 1.00 | 1.26 | 25 |
| 5 | 35 | 0.77 | 0.54 | 0.97 | 0.80 | 0.51 | 1.00 | 1.02 | 31 |
| 5 | 36 | 0.76 | 0.51 | 0.96 | 0.76 | 0.35 | 1.00 | 1.10 | 23 |
| 5 | 37 | 0.78 | 0.55 | 0.97 | 0.72 | 0.22 | 1.00 | 1.16 | 20 |
| 5 | 38 | 0.59 | 0.30 | 0.93 | 0.46 | -0.11 | 1.00 | 1.62 | 13 |
| 5 | 39 | 0.69 | 0.43 | 0.95 | 0.64 | 0.05 | 1.00 | 1.32 | 25 |
| 5 | 40 | 0.83 | 0.62 | 0.98 | 0.76 | 0.37 | 1.00 | 1.04 | 20 |
| 5 | 41 | 0.67 | 0.40 | 0.94 | 0.56 | 0.19 | 0.92 | 1.34 | 23 |
| 5 | 42 | 0.54 | 0.28 | 0.91 | 0.54 | 0.08 | 1.00 | 1.41 | 23 |
| 5 | 43 | 0.54 | 0.27 | 0.91 | 0.59 | 0.12 | 1.00 | 1.36 | 20 |
| 5 | 44 | 0.63 | 0.35 | 0.93 | 0.59 | 0.44 | 0.75 | 1.25 | 19 |
| 5 | 45 | 0.36 | 0.14 | 0.84 | 0.55 | 0.29 | 0.80 | 1.39 | 18 |
| 5 | 46 | 0.21 | 0.06 | 0.72 | 0.54 | 0.19 | 0.90 | 1.49 | 22 |
| 5 | 47 | 0.55 | 0.28 | 0.91 | 0.46 | 0.23 | 0.69 | 1.48 | 25 |
| 5 | 48 | 0.61 | 0.33 | 0.93 | 0.53 | 0.14 | 0.92 | 1.44 | 17 |
| 5 | 49 | -0.02 | -0.04 | 0.16 | 0.91 | 0.86 | 0.96 | 0.53 | 23 |
| 5 | 50 | 0.56 | 0.30 | 0.92 | 0.78 | 0.48 | 1.00 | 1.23 | 26 |
| 6 | 1 | 0.87 | 0.69 | 0.98 | 0.85 | 0.51 | 1.00 | 0.96 | 23 |
| 6 | 2 | 0.90 | 0.75 | 0.99 | 0.85 | 0.67 | 1.00 | 0.95 | 27 |
| 6 | 3 | 0.42 | 0.18 | 0.87 | 0.82 | 0.43 | 1.00 | 1.00 | 23 |
| 6 | 4 | 0.82 | 0.61 | 0.97 | 0.88 | 0.67 | 1.00 | 0.89 | 28 |
| 6 | 5 | 0.86 | 0.67 | 0.98 | 0.80 | 0.37 | 1.00 | 0.99 | 17 |
| 6 | 6 | 0.80 | 0.58 | 0.97 | 0.69 | 0.28 | 1.00 | 1.19 | 29 |
| 6 | 7 | 0.65 | 0.35 | 0.94 | 0.78 | 0.32 | 1.00 | 1.18 | 12 |
| 6 | 8 | 0.77 | 0.53 | 0.97 | 0.78 | 0.34 | 1.00 | 1.20 | 22 |
| 6 | 9 | 0.73 | 0.48 | 0.96 | 0.61 | 0.15 | 1.00 | 1.35 | 28 |
| 6 | 10 | 0.66 | 0.39 | 0.94 | 0.52 | 0.26 | 0.77 | 1.58 | 18 |
| 6 | 11 | 0.69 | 0.42 | 0.95 | 0.72 | 0.33 | 1.00 | 1.16 | 21 |
| 6 | 12 | 0.80 | 0.58 | 0.97 | 0.74 | 0.45 | 1.00 | 1.14 | 26 |
| 6 | 13 | 0.70 | 0.44 | 0.95 | 0.58 | 0.35 | 0.82 | 1.27 | 23 |
| 6 | 14 | 0.58 | 0.30 | 0.92 | 0.56 | 0.34 | 0.78 | 1.50 | 14 |
| 6 | 15 | 0.49 | 0.22 | 0.90 | 0.60 | 0.19 | 1.00 | 1.31 | 15 |
| 6 | 16 | 0.62 | 0.34 | 0.94 | 0.51 | 0.16 | 0.85 | 1.47 | 14 |
| 6 | 17 | 0.68 | 0.42 | 0.95 | 0.61 | 0.26 | 0.97 | 1.39 | 22 |
| 6 | 18 | 0.78 | 0.55 | 0.97 | 0.68 | 0.30 | 1.00 | 1.22 | 22 |
| 6 | 19 | 0.65 | 0.38 | 0.94 | 0.79 | 0.39 | 1.00 | 1.07 | 20 |
| 6 | 20 | 0.82 | 0.61 | 0.98 | 0.83 | 0.43 | 1.00 | 0.96 | 17 |
| 6 | 21 | 0.67 | 0.39 | 0.94 | 0.61 | 0.35 | 0.88 | 1.22 | 19 |
| 6 | 22 | 0.76 | 0.51 | 0.96 | 0.67 | 0.33 | 1.00 | 1.19 | 17 |
| 6 | 23 | 0.61 | 0.34 | 0.93 | 0.71 | 0.19 | 1.00 | 1.21 | 23 |
| 6 | 24 | 0.69 | 0.43 | 0.95 | 0.59 | 0.04 | 1.00 | 1.37 | 30 |
| 6 | 25 | 0.57 | 0.30 | 0.92 | 0.52 | 0.22 | 0.83 | 1.34 | 21 |
| 6 | 26 | 0.60 | 0.33 | 0.93 | 0.52 | 0.10 | 0.94 | 1.37 | 28 |
| 6 | 27 | 0.50 | 0.23 | 0.90 | 0.51 | 0.22 | 0.81 | 1.42 | 18 |
| 6 | 28 | 0.63 | 0.35 | 0.94 | 0.55 | -0.03 | 1.00 | 1.42 | 17 |
| 6 | 29 | 0.42 | 0.18 | 0.87 | 0.45 | 0.29 | 0.62 | 1.49 | 21 |
| 6 | 30 | 0.36 | 0.14 | 0.83 | 0.46 | -0.02 | 0.94 | 1.62 | 22 |
| 6 | 31 | 0.68 | 0.41 | 0.95 | 0.63 | 0.29 | 0.97 | 1.19 | 19 |
| 6 | 32 | 0.71 | 0.45 | 0.96 | 0.66 | 0.25 | 1.00 | 1.21 | 17 |
| 6 | 33 | 0.76 | 0.50 | 0.96 | 0.66 | 0.26 | 1.00 | 1.22 | 18 |
| 6 | 34 | 0.87 | 0.69 | 0.98 | 0.79 | 0.40 | 1.00 | 1.05 | 23 |
| 6 | 35 | 0.67 | 0.39 | 0.94 | 0.77 | 0.41 | 1.00 | 1.22 | 20 |
| 6 | 36 | 0.97 | 0.91 | 1.00 | 0.93 | 0.79 | 1.00 | 0.51 | 12 |
| 6 | 37 | 0.71 | 0.45 | 0.95 | 0.67 | 0.51 | 0.82 | 1.19 | 18 |
| 6 | 38 | 0.76 | 0.51 | 0.96 | 0.71 | 0.51 | 0.90 | 1.19 | 21 |
| 6 | 39 | 0.62 | 0.33 | 0.93 | 0.70 | 0.38 | 1.00 | 1.17 | 16 |
| 6 | 40 | 0.89 | 0.73 | 0.99 | 0.81 | 0.63 | 0.99 | 0.92 | 24 |
| 6 | 41 | 0.63 | 0.36 | 0.93 | 0.55 | 0.13 | 0.96 | 1.32 | 28 |
| 6 | 42 | 0.58 | 0.31 | 0.92 | 0.63 | 0.18 | 1.00 | 1.33 | 21 |
| 6 | 43 | 0.68 | 0.42 | 0.95 | 0.67 | 0.37 | 0.97 | 1.15 | 34 |
| 6 | 44 | 0.70 | 0.43 | 0.95 | 0.56 | 0.36 | 0.75 | 1.37 | 26 |
| 6 | 45 | 0.47 | 0.22 | 0.88 | 0.66 | 0.41 | 0.92 | 1.20 | 31 |
| 6 | 46 | 0.23 | 0.06 | 0.74 | 0.65 | 0.12 | 1.00 | 1.38 | 17 |
| 6 | 47 | 0.62 | 0.34 | 0.93 | 0.55 | 0.27 | 0.84 | 1.32 | 24 |
| 6 | 48 | 0.72 | 0.45 | 0.96 | 0.68 | 0.53 | 0.83 | 1.23 | 19 |
| 6 | 49 | 0.08 | -0.03 | 0.58 | 0.86 | 0.59 | 1.00 | 0.66 | 12 |
| 6 | 50 | 0.52 | 0.25 | 0.90 | 0.87 | 0.61 | 1.00 | 1.02 | 23 |
| 7 | 1 | 0.57 | 0.31 | 0.92 | 0.79 | 0.28 | 1.00 | 1.32 | 26 |
| 7 | 2 | 0.94 | 0.85 | 0.99 | 0.91 | 0.76 | 1.00 | 0.69 | 31 |
| 7 | 3 | 0.38 | 0.15 | 0.85 | 0.69 | 0.20 | 1.00 | 1.01 | 17 |
| 7 | 4 | 0.65 | 0.38 | 0.94 | 0.82 | 0.51 | 1.00 | 1.17 | 20 |
| 7 | 5 | 0.83 | 0.63 | 0.98 | 0.75 | 0.39 | 1.00 | 1.05 | 21 |
| 7 | 6 | 0.89 | 0.74 | 0.99 | 0.81 | 0.52 | 1.00 | 0.95 | 16 |
| 7 | 7 | 0.75 | 0.49 | 0.96 | 0.82 | 0.46 | 1.00 | 1.05 | 15 |
| 7 | 8 | 0.72 | 0.46 | 0.96 | 0.77 | 0.43 | 1.00 | 1.05 | 20 |
| 7 | 9 | 0.73 | 0.48 | 0.96 | 0.60 | 0.34 | 0.86 | 1.25 | 22 |
| 7 | 10 | 0.76 | 0.52 | 0.96 | 0.65 | 0.28 | 1.00 | 1.16 | 20 |
| 7 | 11 | 0.83 | 0.62 | 0.98 | 0.81 | 0.51 | 1.00 | 0.94 | 21 |
| 7 | 12 | 0.66 | 0.37 | 0.94 | 0.59 | 0.18 | 1.00 | 1.47 | 14 |
| 7 | 13 | 0.59 | 0.32 | 0.93 | 0.48 | 0.33 | 0.64 | 1.44 | 23 |
| 7 | 14 | 0.74 | 0.48 | 0.96 | 0.64 | 0.21 | 1.00 | 1.23 | 22 |
| 7 | 15 | 0.71 | 0.45 | 0.95 | 0.62 | 0.34 | 0.90 | 1.25 | 25 |
| 7 | 16 | 0.72 | 0.46 | 0.96 | 0.59 | 0.21 | 0.98 | 1.31 | 25 |
| 7 | 17 | 0.78 | 0.55 | 0.97 | 0.71 | 0.36 | 1.00 | 1.14 | 27 |
| 7 | 18 | 0.80 | 0.58 | 0.97 | 0.69 | 0.28 | 1.00 | 1.18 | 18 |
| 7 | 19 | 0.72 | 0.46 | 0.96 | 0.82 | 0.42 | 1.00 | 1.03 | 20 |
| 7 | 20 | 0.67 | 0.40 | 0.94 | 0.71 | 0.19 | 1.00 | 1.25 | 24 |
| 7 | 21 | 0.76 | 0.52 | 0.96 | 0.70 | 0.45 | 0.94 | 1.07 | 22 |
| 7 | 22 | 0.83 | 0.63 | 0.98 | 0.76 | 0.45 | 1.00 | 0.97 | 22 |
| 7 | 23 | 0.55 | 0.28 | 0.91 | 0.61 | 0.12 | 1.00 | 1.37 | 22 |
| 7 | 24 | 0.69 | 0.43 | 0.95 | 0.65 | 0.13 | 1.00 | 1.28 | 31 |
| 7 | 25 | 0.62 | 0.34 | 0.93 | 0.51 | 0.13 | 0.88 | 1.40 | 23 |
| 7 | 26 | 0.57 | 0.30 | 0.92 | 0.55 | 0.02 | 1.00 | 1.41 | 17 |
| 7 | 27 | 0.75 | 0.50 | 0.96 | 0.65 | 0.16 | 1.00 | 1.21 | 24 |
| 7 | 28 | 0.62 | 0.35 | 0.93 | 0.55 | 0.23 | 0.88 | 1.32 | 25 |
| 7 | 29 | 0.38 | 0.15 | 0.85 | 0.52 | 0.04 | 1.00 | 1.46 | 18 |
| 7 | 30 | 0.50 | 0.24 | 0.90 | 0.49 | 0.07 | 0.91 | 1.46 | 27 |
| 7 | 31 | 0.54 | 0.28 | 0.91 | 0.50 | 0.32 | 0.67 | 1.41 | 33 |
| 7 | 32 | 0.61 | 0.33 | 0.93 | 0.55 | 0.33 | 0.76 | 1.38 | 20 |
| 7 | 33 | 0.80 | 0.56 | 0.97 | 0.69 | 0.19 | 1.00 | 1.20 | 15 |
| 7 | 34 | 0.73 | 0.48 | 0.96 | 0.62 | 0.43 | 0.81 | 1.22 | 21 |
| 7 | 35 | 0.85 | 0.65 | 0.98 | 0.82 | 0.44 | 1.00 | 0.95 | 17 |
| 7 | 36 | 0.74 | 0.48 | 0.96 | 0.76 | 0.32 | 1.00 | 1.14 | 19 |
| 7 | 37 | 0.67 | 0.40 | 0.95 | 0.57 | 0.09 | 1.00 | 1.34 | 22 |
| 7 | 38 | 0.79 | 0.55 | 0.97 | 0.70 | 0.18 | 1.00 | 1.13 | 18 |
| 7 | 39 | 0.60 | 0.31 | 0.93 | 0.48 | -0.32 | 1.00 | 1.55 | 13 |
| 7 | 40 | 0.77 | 0.53 | 0.97 | 0.75 | 0.31 | 1.00 | 1.05 | 17 |
| 7 | 41 | 0.68 | 0.40 | 0.95 | 0.64 | 0.17 | 1.00 | 1.26 | 17 |
| 7 | 42 | 0.55 | 0.28 | 0.91 | 0.47 | 0.18 | 0.76 | 1.51 | 24 |
| 7 | 43 | 0.78 | 0.54 | 0.97 | 0.65 | 0.26 | 1.00 | 1.24 | 26 |
| 7 | 44 | 0.69 | 0.42 | 0.95 | 0.57 | 0.13 | 1.00 | 1.35 | 15 |
| 7 | 45 | 0.46 | 0.21 | 0.88 | 0.59 | 0.09 | 1.00 | 1.36 | 26 |
| 7 | 46 | 0.41 | 0.18 | 0.86 | 0.71 | 0.53 | 0.89 | 1.17 | 29 |
| 7 | 47 | 0.75 | 0.49 | 0.96 | 0.67 | 0.37 | 0.96 | 1.14 | 18 |
| 7 | 48 | 0.62 | 0.34 | 0.93 | 0.60 | 0.37 | 0.83 | 1.28 | 17 |
| 7 | 49 | -0.01 | -0.05 | 0.25 | 0.91 | 0.82 | 1.00 | 0.74 | 17 |
| 7 | 50 | 0.61 | 0.34 | 0.93 | 0.77 | 0.46 | 1.00 | 1.25 | 18 |
| 8 | 1 | 0.75 | 0.49 | 0.96 | 0.77 | 0.33 | 1.00 | 1.35 | 17 |
| 8 | 2 | 0.85 | 0.65 | 0.98 | 0.80 | 0.49 | 1.00 | 1.05 | 24 |
| 8 | 3 | 0.41 | 0.17 | 0.86 | 0.86 | 0.65 | 1.00 | 1.07 | 19 |
| 8 | 4 | 0.68 | 0.41 | 0.95 | 0.83 | 0.49 | 1.00 | 1.16 | 18 |
| 8 | 5 | 0.78 | 0.54 | 0.97 | 0.71 | 0.21 | 1.00 | 1.19 | 25 |
| 8 | 6 | 0.85 | 0.66 | 0.98 | 0.79 | 0.38 | 1.00 | 0.99 | 18 |
| 8 | 7 | 0.53 | 0.26 | 0.91 | 0.82 | 0.40 | 1.00 | 1.12 | 21 |
| 8 | 8 | 0.84 | 0.65 | 0.98 | 0.86 | 0.52 | 1.00 | 0.92 | 23 |
| 8 | 9 | 0.67 | 0.40 | 0.95 | 0.62 | 0.21 | 1.00 | 1.25 | 20 |
| 8 | 10 | 0.84 | 0.64 | 0.98 | 0.74 | 0.45 | 1.00 | 0.99 | 14 |
| 8 | 11 | 0.62 | 0.35 | 0.93 | 0.81 | 0.55 | 1.00 | 0.99 | 20 |
| 8 | 12 | 0.70 | 0.44 | 0.95 | 0.68 | 0.19 | 1.00 | 1.25 | 29 |
| 8 | 13 | 0.63 | 0.35 | 0.94 | 0.50 | 0.10 | 0.90 | 1.47 | 20 |
| 8 | 14 | 0.78 | 0.54 | 0.97 | 0.69 | 0.34 | 1.00 | 1.12 | 25 |
| 8 | 15 | 0.60 | 0.33 | 0.93 | 0.57 | 0.44 | 0.70 | 1.38 | 24 |
| 8 | 16 | 0.82 | 0.61 | 0.97 | 0.73 | 0.45 | 1.00 | 1.06 | 22 |
| 8 | 17 | 0.63 | 0.35 | 0.94 | 0.67 | 0.06 | 1.00 | 1.32 | 17 |
| 8 | 18 | 0.74 | 0.48 | 0.96 | 0.64 | 0.13 | 1.00 | 1.43 | 15 |
| 8 | 19 | 0.31 | 0.10 | 0.80 | 0.64 | 0.28 | 0.99 | 1.20 | 19 |
| 8 | 20 | 0.77 | 0.53 | 0.97 | 0.87 | 0.61 | 1.00 | 1.06 | 23 |
| 8 | 21 | 0.71 | 0.44 | 0.95 | 0.60 | 0.21 | 0.99 | 1.30 | 17 |
| 8 | 22 | 0.68 | 0.42 | 0.95 | 0.56 | 0.15 | 0.97 | 1.36 | 25 |
| 8 | 23 | 0.69 | 0.43 | 0.95 | 0.65 | 0.13 | 1.00 | 1.24 | 23 |
| 8 | 24 | 0.78 | 0.54 | 0.97 | 0.72 | 0.24 | 1.00 | 1.15 | 23 |
| 8 | 25 | 0.51 | 0.25 | 0.90 | 0.42 | 0.15 | 0.69 | 1.50 | 24 |
| 8 | 26 | 0.67 | 0.40 | 0.94 | 0.55 | 0.13 | 0.97 | 1.33 | 28 |
| 8 | 27 | 0.41 | 0.18 | 0.86 | 0.58 | 0.22 | 0.94 | 1.38 | 30 |
| 8 | 28 | 0.58 | 0.28 | 0.92 | 0.66 | 0.36 | 0.96 | 1.22 | 11 |
| 8 | 29 | 0.64 | 0.36 | 0.94 | 0.62 | 0.23 | 1.00 | 1.22 | 21 |
| 8 | 30 | 0.47 | 0.22 | 0.89 | 0.66 | 0.23 | 1.00 | 1.15 | 23 |
| 8 | 31 | 0.30 | 0.11 | 0.80 | 0.30 | 0.13 | 0.47 | 1.71 | 22 |
| 8 | 32 | 0.78 | 0.54 | 0.97 | 0.69 | 0.44 | 0.95 | 1.12 | 21 |
| 8 | 33 | 0.52 | 0.26 | 0.90 | 0.50 | -0.30 | 1.00 | 1.57 | 28 |
| 8 | 34 | 0.74 | 0.49 | 0.96 | 0.62 | 0.03 | 1.00 | 1.32 | 26 |
| 8 | 35 | 0.85 | 0.65 | 0.98 | 0.88 | 0.65 | 1.00 | 0.92 | 18 |
| 8 | 36 | 0.72 | 0.46 | 0.96 | 0.71 | 0.27 | 1.00 | 1.17 | 22 |
| 8 | 37 | 0.62 | 0.34 | 0.93 | 0.51 | 0.02 | 1.00 | 1.45 | 17 |
| 8 | 38 | 0.82 | 0.60 | 0.97 | 0.76 | 0.50 | 1.00 | 1.02 | 18 |
| 8 | 39 | 0.71 | 0.44 | 0.95 | 0.70 | 0.18 | 1.00 | 1.16 | 15 |
| 8 | 40 | 0.84 | 0.64 | 0.98 | 0.77 | 0.61 | 0.93 | 1.02 | 17 |
| 8 | 41 | 0.69 | 0.42 | 0.95 | 0.68 | 0.59 | 0.77 | 1.09 | 18 |
| 8 | 42 | 0.58 | 0.31 | 0.92 | 0.50 | 0.09 | 0.92 | 1.41 | 23 |
| 8 | 43 | 0.42 | 0.18 | 0.86 | 0.47 | 0.08 | 0.86 | 1.48 | 25 |
| 8 | 44 | 0.63 | 0.36 | 0.93 | 0.61 | 0.39 | 0.82 | 1.22 | 25 |
| 8 | 45 | 0.19 | 0.05 | 0.70 | 0.64 | 0.44 | 0.84 | 1.36 | 23 |
| 8 | 46 | 0.48 | 0.22 | 0.89 | 0.61 | 0.13 | 1.00 | 1.32 | 20 |
| 8 | 47 | 0.49 | 0.24 | 0.89 | 0.52 | 0.20 | 0.84 | 1.37 | 30 |
| 8 | 48 | 0.49 | 0.23 | 0.89 | 0.44 | 0.11 | 0.78 | 1.50 | 20 |
| 8 | 49 | 0.10 | 0.01 | 0.55 | 0.92 | 0.79 | 1.00 | 0.73 | 23 |
| 8 | 50 | 0.66 | 0.38 | 0.94 | 0.77 | 0.41 | 1.00 | 1.23 | 21 |
| 9 | 1 | 0.47 | 0.21 | 0.88 | 0.82 | 0.39 | 1.00 | 1.21 | 21 |
| 9 | 2 | 0.70 | 0.43 | 0.95 | 0.66 | 0.41 | 0.92 | 1.26 | 18 |
| 9 | 3 | 0.05 | -0.01 | 0.42 | 0.81 | 0.61 | 1.00 | 1.15 | 25 |
| 9 | 4 | 0.51 | 0.24 | 0.90 | 0.88 | 0.59 | 1.00 | 0.82 | 19 |
| 9 | 5 | 0.64 | 0.36 | 0.94 | 0.65 | 0.08 | 1.00 | 1.34 | 16 |
| 9 | 6 | 0.75 | 0.50 | 0.96 | 0.63 | 0.15 | 1.00 | 1.27 | 22 |
| 9 | 7 | 0.53 | 0.26 | 0.91 | 0.87 | 0.55 | 1.00 | 1.00 | 17 |
| 9 | 8 | 0.74 | 0.48 | 0.96 | 0.81 | 0.39 | 1.00 | 1.13 | 17 |
| 9 | 9 | 0.59 | 0.31 | 0.93 | 0.46 | 0.10 | 0.82 | 1.48 | 18 |
| 9 | 10 | 0.67 | 0.40 | 0.94 | 0.53 | 0.19 | 0.88 | 1.39 | 21 |
| 9 | 11 | 0.78 | 0.55 | 0.97 | 0.81 | 0.59 | 1.00 | 0.97 | 27 |
| 9 | 12 | 0.71 | 0.44 | 0.95 | 0.68 | 0.25 | 1.00 | 1.18 | 16 |
| 9 | 13 | 0.71 | 0.44 | 0.95 | 0.59 | 0.30 | 0.88 | 1.24 | 18 |
| 9 | 14 | 0.71 | 0.45 | 0.95 | 0.65 | 0.39 | 0.90 | 1.19 | 21 |
| 9 | 15 | 0.67 | 0.38 | 0.95 | 0.63 | 0.31 | 0.96 | 1.40 | 13 |
| 9 | 16 | 0.82 | 0.60 | 0.97 | 0.73 | 0.37 | 1.00 | 1.13 | 24 |
| 9 | 17 | 0.45 | 0.20 | 0.88 | 0.55 | -0.06 | 1.00 | 1.48 | 18 |
| 9 | 18 | 0.73 | 0.48 | 0.96 | 0.69 | 0.17 | 1.00 | 1.22 | 20 |
| 9 | 19 | 0.30 | 0.11 | 0.80 | 0.70 | 0.21 | 1.00 | 1.21 | 23 |
| 9 | 20 | 0.55 | 0.28 | 0.91 | 0.74 | 0.25 | 1.00 | 1.18 | 24 |
| 9 | 21 | 0.57 | 0.30 | 0.92 | 0.58 | 0.11 | 1.00 | 1.33 | 24 |
| 9 | 22 | 0.75 | 0.50 | 0.96 | 0.66 | 0.27 | 1.00 | 1.18 | 19 |
| 9 | 23 | 0.57 | 0.30 | 0.92 | 0.72 | 0.27 | 1.00 | 1.16 | 29 |
| 9 | 24 | 0.63 | 0.36 | 0.93 | 0.71 | 0.14 | 1.00 | 1.23 | 27 |
| 9 | 25 | 0.38 | 0.15 | 0.84 | 0.38 | -0.06 | 0.81 | 1.65 | 25 |
| 9 | 26 | 0.60 | 0.31 | 0.93 | 0.56 | 0.21 | 0.92 | 1.29 | 12 |
| 9 | 27 | 0.61 | 0.33 | 0.93 | 0.65 | 0.26 | 1.00 | 1.23 | 17 |
| 9 | 28 | 0.64 | 0.37 | 0.94 | 0.64 | 0.29 | 0.98 | 1.19 | 22 |
| 9 | 29 | 0.18 | 0.05 | 0.68 | 0.45 | 0.05 | 0.84 | 1.52 | 26 |
| 9 | 30 | 0.34 | 0.12 | 0.82 | 0.64 | 0.29 | 0.98 | 1.28 | 21 |
| 9 | 31 | 0.50 | 0.24 | 0.89 | 0.58 | 0.43 | 0.73 | 1.28 | 23 |
| 9 | 32 | 0.65 | 0.37 | 0.94 | 0.60 | 0.41 | 0.80 | 1.26 | 19 |
| 9 | 33 | 0.71 | 0.45 | 0.95 | 0.66 | 0.22 | 1.00 | 1.19 | 23 |
| 9 | 34 | 0.61 | 0.33 | 0.93 | 0.51 | 0.16 | 0.86 | 1.42 | 19 |
| 9 | 35 | 0.56 | 0.28 | 0.92 | 0.69 | 0.09 | 1.00 | 1.24 | 15 |
| 9 | 36 | 0.51 | 0.24 | 0.90 | 0.67 | 0.04 | 1.00 | 1.33 | 17 |
| 9 | 37 | 0.56 | 0.28 | 0.92 | 0.56 | 0.07 | 1.00 | 1.37 | 18 |
| 9 | 38 | 0.65 | 0.38 | 0.94 | 0.57 | 0.27 | 0.87 | 1.34 | 24 |
| 9 | 39 | 0.60 | 0.32 | 0.93 | 0.54 | 0.00 | 1.00 | 1.46 | 20 |
| 9 | 40 | 0.60 | 0.33 | 0.93 | 0.63 | 0.06 | 1.00 | 1.35 | 23 |
| 9 | 41 | 0.46 | 0.22 | 0.88 | 0.48 | 0.34 | 0.61 | 1.44 | 29 |
| 9 | 42 | 0.36 | 0.15 | 0.83 | 0.49 | 0.18 | 0.80 | 1.45 | 31 |
| 9 | 43 | 0.58 | 0.31 | 0.92 | 0.62 | 0.42 | 0.82 | 1.23 | 26 |
| 9 | 44 | 0.68 | 0.41 | 0.95 | 0.61 | 0.37 | 0.86 | 1.21 | 24 |
| 9 | 45 | 0.29 | 0.10 | 0.79 | 0.48 | 0.35 | 0.62 | 1.46 | 23 |
| 9 | 46 | 0.12 | 0.02 | 0.60 | 0.46 | 0.25 | 0.67 | 1.56 | 21 |
| 9 | 47 | 0.52 | 0.25 | 0.90 | 0.52 | 0.13 | 0.91 | 1.38 | 18 |
| 9 | 48 | 0.69 | 0.43 | 0.95 | 0.65 | 0.47 | 0.82 | 1.20 | 33 |
| 9 | 49 | 0.07 | 0.00 | 0.50 | 0.89 | 0.73 | 1.00 | 0.59 | 22 |
| 9 | 50 | 0.77 | 0.52 | 0.97 | 0.86 | 0.60 | 1.00 | 0.98 | 21 |
| 10 | 1 | 0.67 | 0.40 | 0.95 | 0.69 | 0.05 | 1.00 | 1.43 | 23 |
| 10 | 2 | 0.83 | 0.62 | 0.98 | 0.78 | 0.40 | 1.00 | 1.06 | 18 |
| 10 | 3 | 0.50 | 0.23 | 0.90 | 0.76 | 0.38 | 1.00 | 1.13 | 15 |
| 10 | 4 | 0.75 | 0.50 | 0.96 | 0.86 | 0.51 | 1.00 | 0.96 | 26 |
| 10 | 5 | 0.71 | 0.45 | 0.95 | 0.68 | 0.07 | 1.00 | 1.23 | 18 |
| 10 | 6 | 0.77 | 0.53 | 0.97 | 0.67 | 0.21 | 1.00 | 1.25 | 27 |
| 10 | 7 | 0.70 | 0.43 | 0.95 | 0.78 | 0.51 | 1.00 | 1.11 | 21 |
| 10 | 8 | 0.75 | 0.50 | 0.96 | 0.79 | 0.28 | 1.00 | 1.18 | 19 |
| 10 | 9 | 0.69 | 0.41 | 0.95 | 0.58 | 0.30 | 0.86 | 1.28 | 19 |
| 10 | 10 | 0.66 | 0.37 | 0.94 | 0.54 | 0.19 | 0.89 | 1.33 | 13 |
| 10 | 11 | 0.48 | 0.22 | 0.89 | 0.58 | 0.17 | 0.98 | 1.38 | 18 |
| 10 | 12 | 0.71 | 0.46 | 0.95 | 0.72 | 0.31 | 1.00 | 1.11 | 25 |
| 10 | 13 | 0.59 | 0.31 | 0.93 | 0.46 | 0.12 | 0.81 | 1.49 | 17 |
| 10 | 14 | 0.65 | 0.38 | 0.94 | 0.57 | 0.21 | 0.94 | 1.30 | 29 |
| 10 | 15 | 0.47 | 0.21 | 0.88 | 0.55 | 0.39 | 0.72 | 1.39 | 19 |
| 10 | 16 | 0.54 | 0.27 | 0.91 | 0.45 | 0.23 | 0.67 | 1.48 | 18 |
| 10 | 17 | 0.68 | 0.41 | 0.95 | 0.61 | 0.16 | 1.00 | 1.31 | 28 |
| 10 | 18 | 0.72 | 0.47 | 0.96 | 0.59 | 0.14 | 1.00 | 1.29 | 30 |
| 10 | 19 | 0.59 | 0.32 | 0.92 | 0.73 | 0.36 | 1.00 | 1.20 | 25 |
| 10 | 20 | 0.85 | 0.65 | 0.98 | 0.87 | 0.60 | 1.00 | 0.89 | 17 |
| 10 | 21 | 0.64 | 0.38 | 0.94 | 0.56 | 0.42 | 0.70 | 1.32 | 28 |
| 10 | 22 | 0.57 | 0.30 | 0.92 | 0.42 | -0.04 | 0.88 | 1.59 | 26 |
| 10 | 23 | 0.61 | 0.32 | 0.93 | 0.66 | 0.28 | 1.00 | 1.22 | 14 |
| 10 | 24 | 0.69 | 0.43 | 0.95 | 0.68 | 0.12 | 1.00 | 1.27 | 26 |
| 10 | 25 | 0.68 | 0.42 | 0.95 | 0.75 | 0.56 | 0.95 | 0.99 | 22 |
| 10 | 26 | 0.48 | 0.23 | 0.89 | 0.55 | 0.28 | 0.82 | 1.36 | 24 |
| 10 | 27 | 0.54 | 0.27 | 0.91 | 0.68 | 0.47 | 0.88 | 1.14 | 19 |
| 10 | 28 | 0.56 | 0.28 | 0.92 | 0.54 | 0.27 | 0.81 | 1.33 | 20 |
| 10 | 29 | 0.41 | 0.17 | 0.86 | 0.61 | 0.38 | 0.85 | 1.27 | 20 |
| 10 | 30 | 0.24 | 0.08 | 0.74 | 0.60 | 0.41 | 0.78 | 1.32 | 27 |
| 10 | 31 | 0.34 | 0.12 | 0.83 | 0.52 | 0.29 | 0.75 | 1.42 | 17 |
| 10 | 32 | 0.39 | 0.17 | 0.85 | 0.48 | 0.34 | 0.62 | 1.49 | 27 |
| 10 | 33 | 0.74 | 0.49 | 0.96 | 0.64 | 0.24 | 1.00 | 1.18 | 18 |
| 10 | 34 | 0.65 | 0.38 | 0.94 | 0.53 | 0.05 | 1.00 | 1.39 | 24 |
| 10 | 35 | 0.46 | 0.21 | 0.88 | 0.78 | 0.41 | 1.00 | 1.19 | 24 |
| 10 | 36 | 0.60 | 0.32 | 0.93 | 0.66 | 0.15 | 1.00 | 1.30 | 19 |
| 10 | 37 | 0.54 | 0.27 | 0.91 | 0.43 | 0.15 | 0.70 | 1.52 | 23 |
| 10 | 38 | 0.77 | 0.51 | 0.97 | 0.64 | 0.37 | 0.90 | 1.07 | 12 |
| 10 | 39 | 0.60 | 0.31 | 0.93 | 0.58 | 0.29 | 0.87 | 1.39 | 16 |
| 10 | 40 | 0.83 | 0.62 | 0.98 | 0.76 | 0.31 | 1.00 | 1.10 | 15 |
| 10 | 41 | 0.57 | 0.30 | 0.92 | 0.59 | 0.34 | 0.84 | 1.26 | 18 |
| 10 | 42 | 0.35 | 0.13 | 0.83 | 0.35 | -0.10 | 0.81 | 1.66 | 22 |
| 10 | 43 | 0.52 | 0.23 | 0.90 | 0.53 | 0.14 | 0.93 | 1.36 | 13 |
| 10 | 44 | 0.69 | 0.42 | 0.95 | 0.62 | 0.40 | 0.85 | 1.21 | 29 |
| 10 | 45 | 0.16 | 0.04 | 0.65 | 0.57 | 0.53 | 0.61 | 1.37 | 30 |
| 10 | 46 | 0.28 | 0.09 | 0.78 | 0.63 | 0.54 | 0.73 | 1.07 | 22 |
| 10 | 47 | 0.48 | 0.22 | 0.89 | 0.56 | 0.23 | 0.90 | 1.32 | 21 |
| 10 | 48 | 0.37 | 0.14 | 0.84 | 0.47 | 0.31 | 0.63 | 1.47 | 19 |
| 10 | 49 | 0.09 | 0.00 | 0.55 | 0.92 | 0.79 | 1.00 | 0.91 | 19 |
| 10 | 50 | 0.54 | 0.27 | 0.91 | 0.75 | 0.38 | 1.00 | 1.30 | 23 |

**Supplementary Figure 7**

*Overview of Reliabilities/Agreement for ICC and Gwet’s AC2 for Each Actor and Video. Point Ranges Specify 95% Confidence Intervals. Dashed Horizontal Line Specifies Threshold for a Reliability of 0.50.*

Actor ID = 1

**
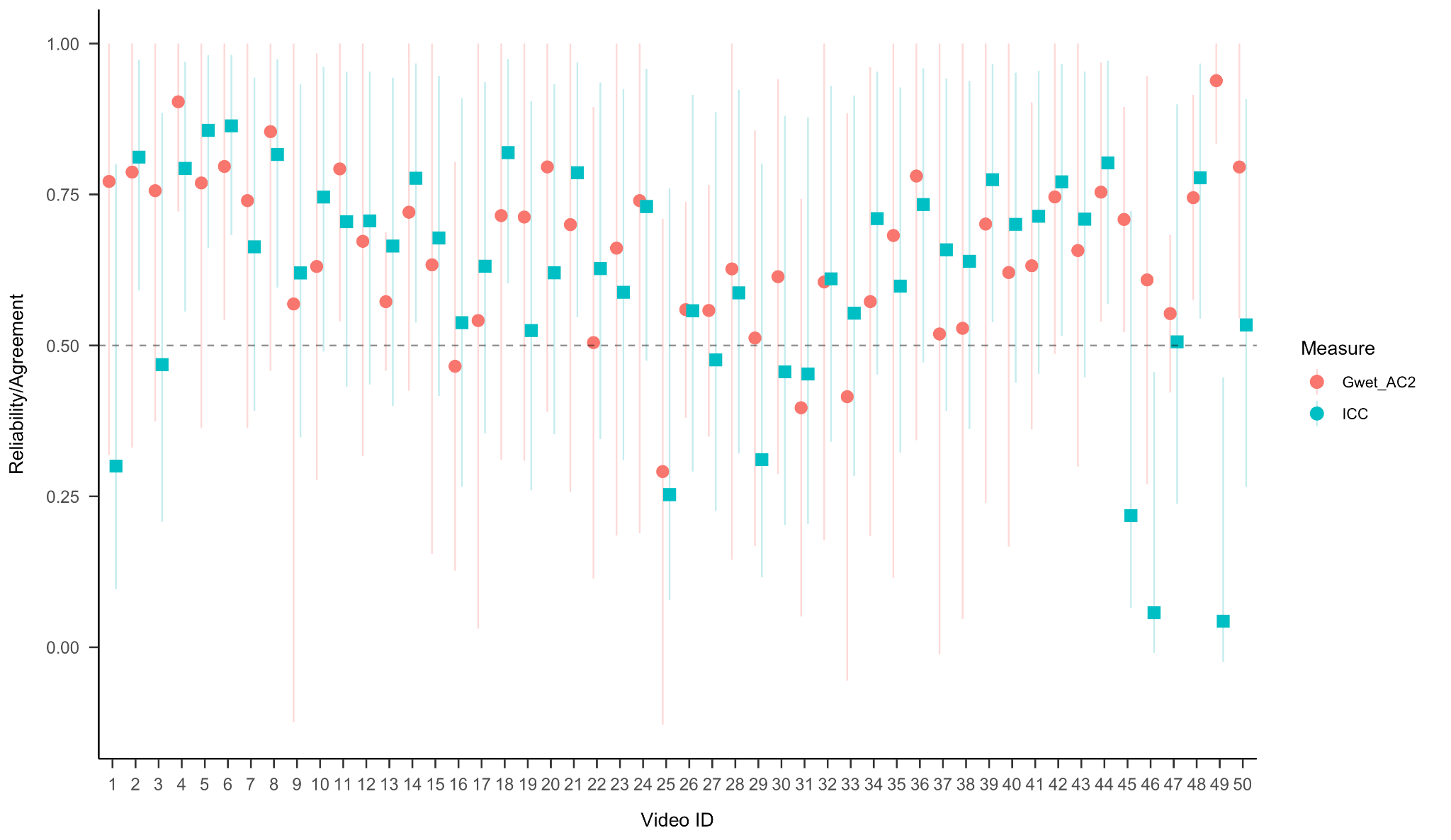
**

Actor ID = 2

**
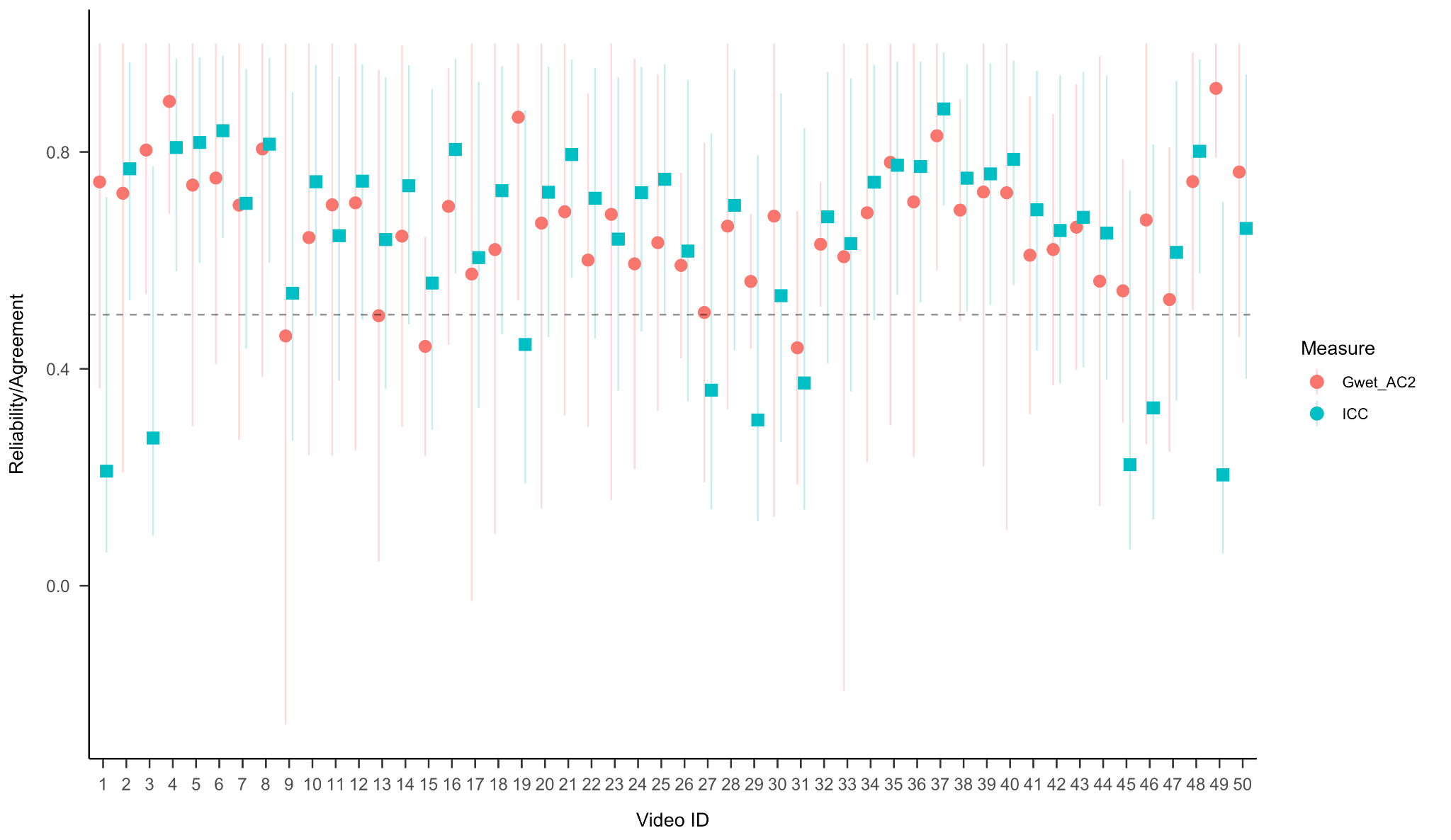
**

Actor ID = 3


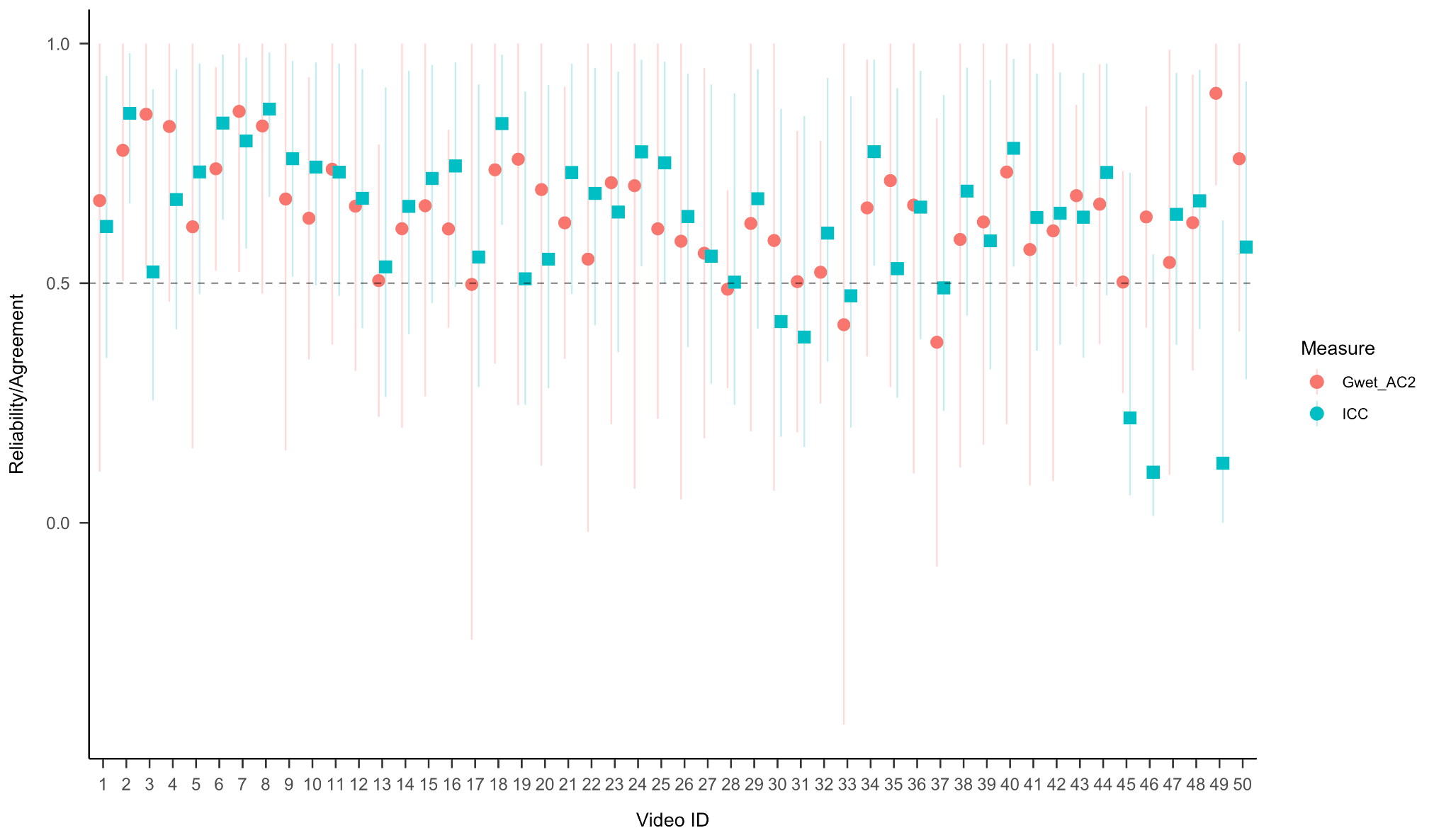


Actor ID = 4


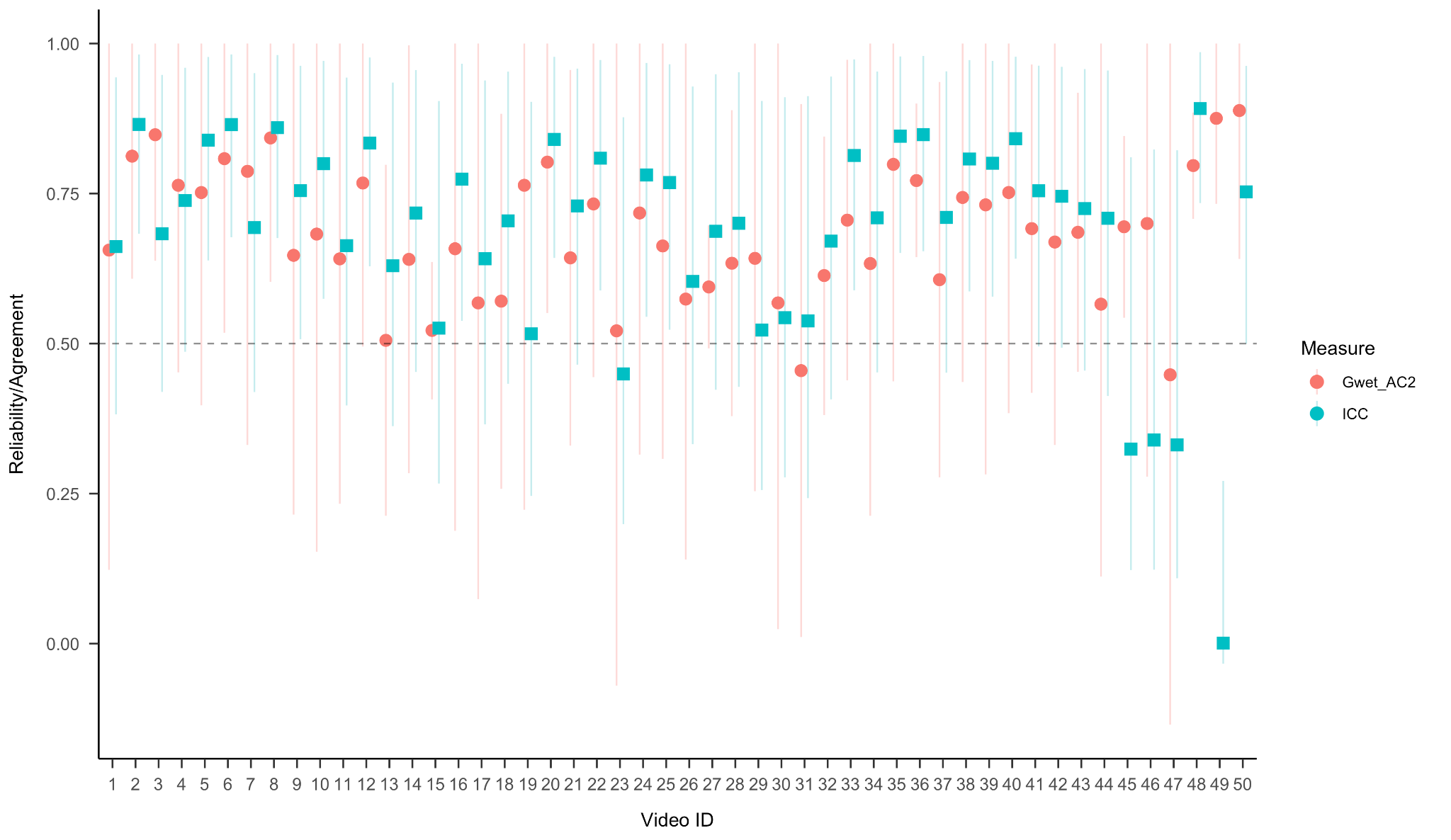


Actor ID = 5


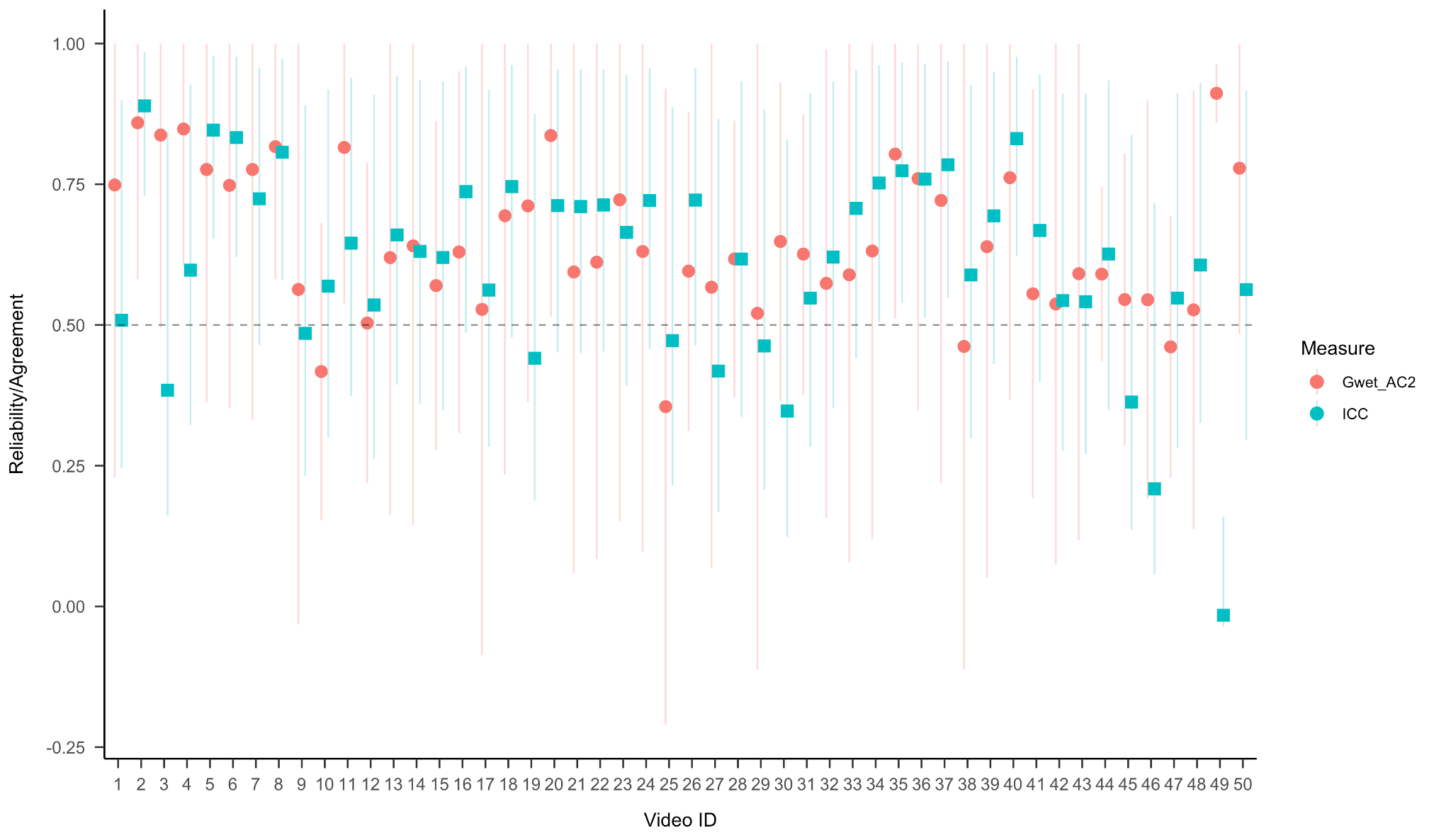


Actor ID = 6


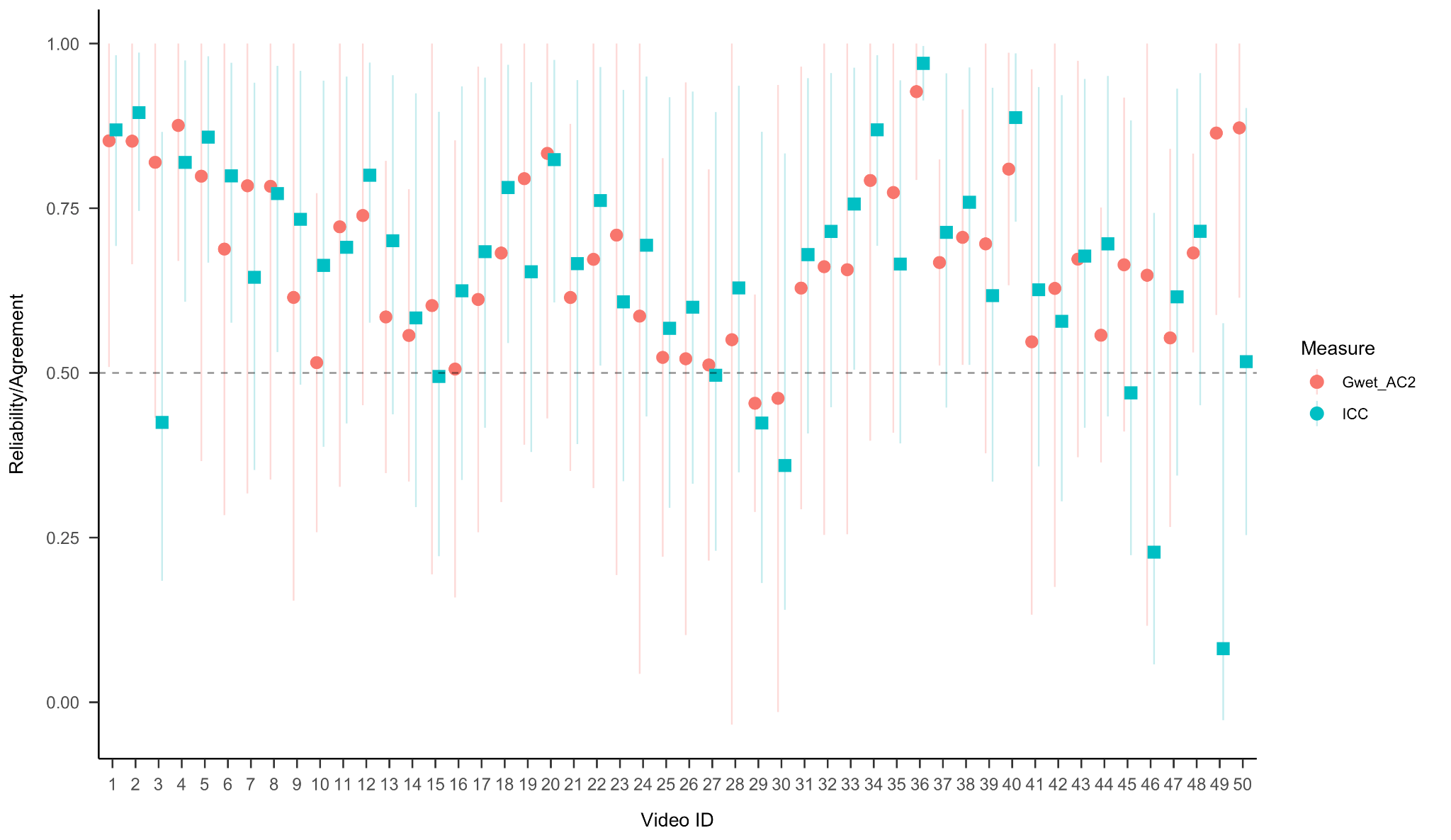


Actor ID = 7


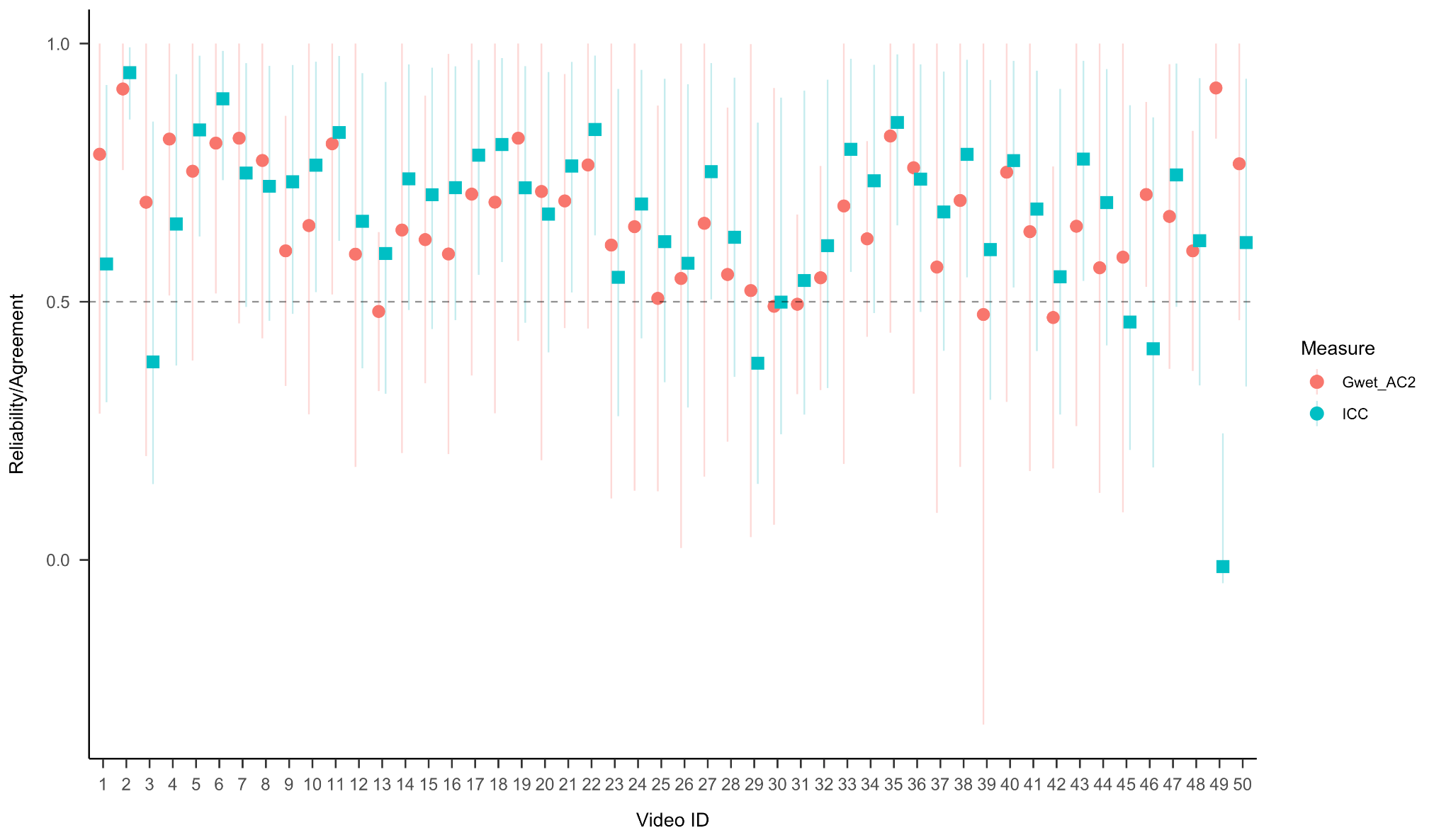


Actor ID = 8


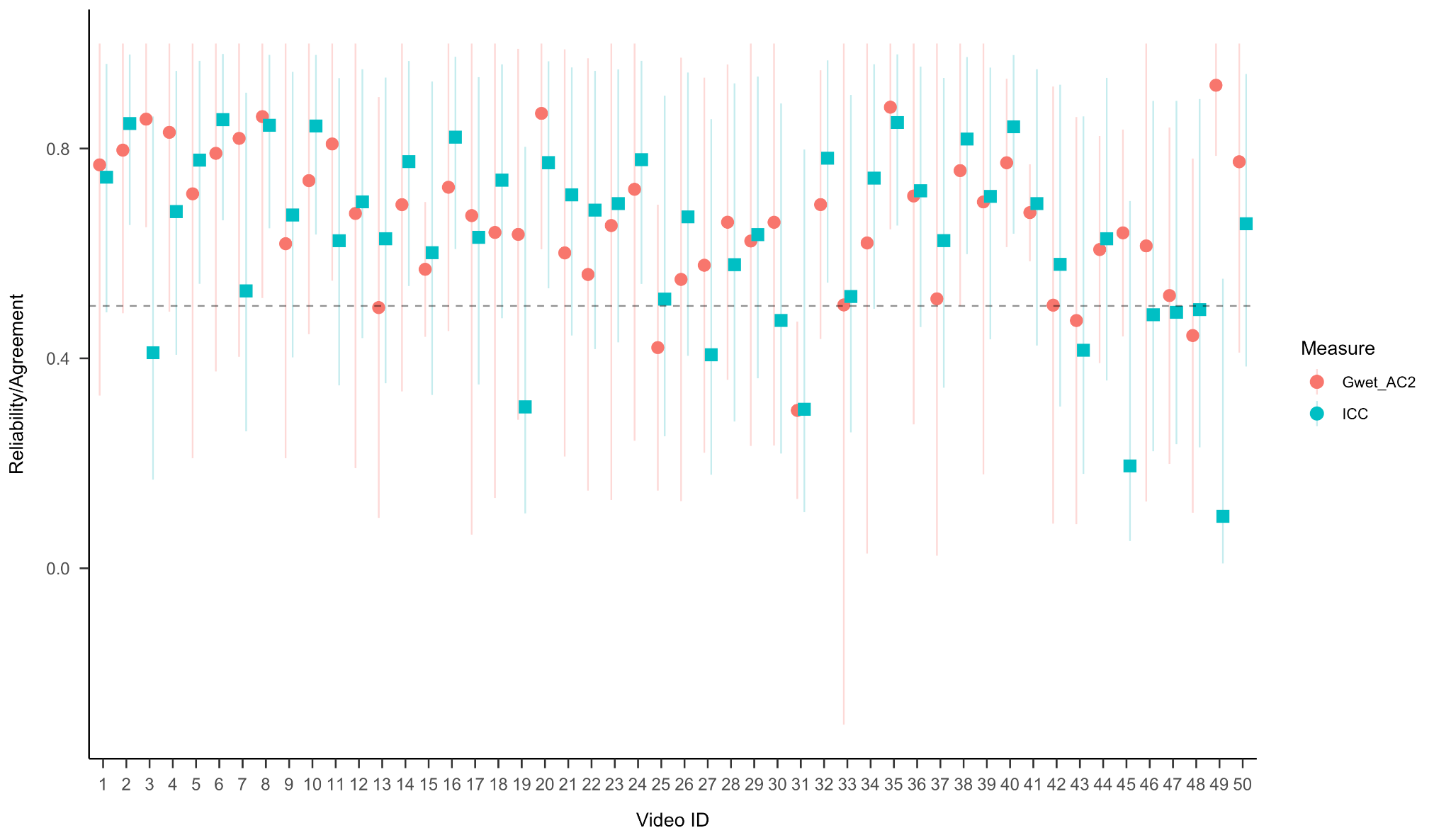


Actor ID = 9


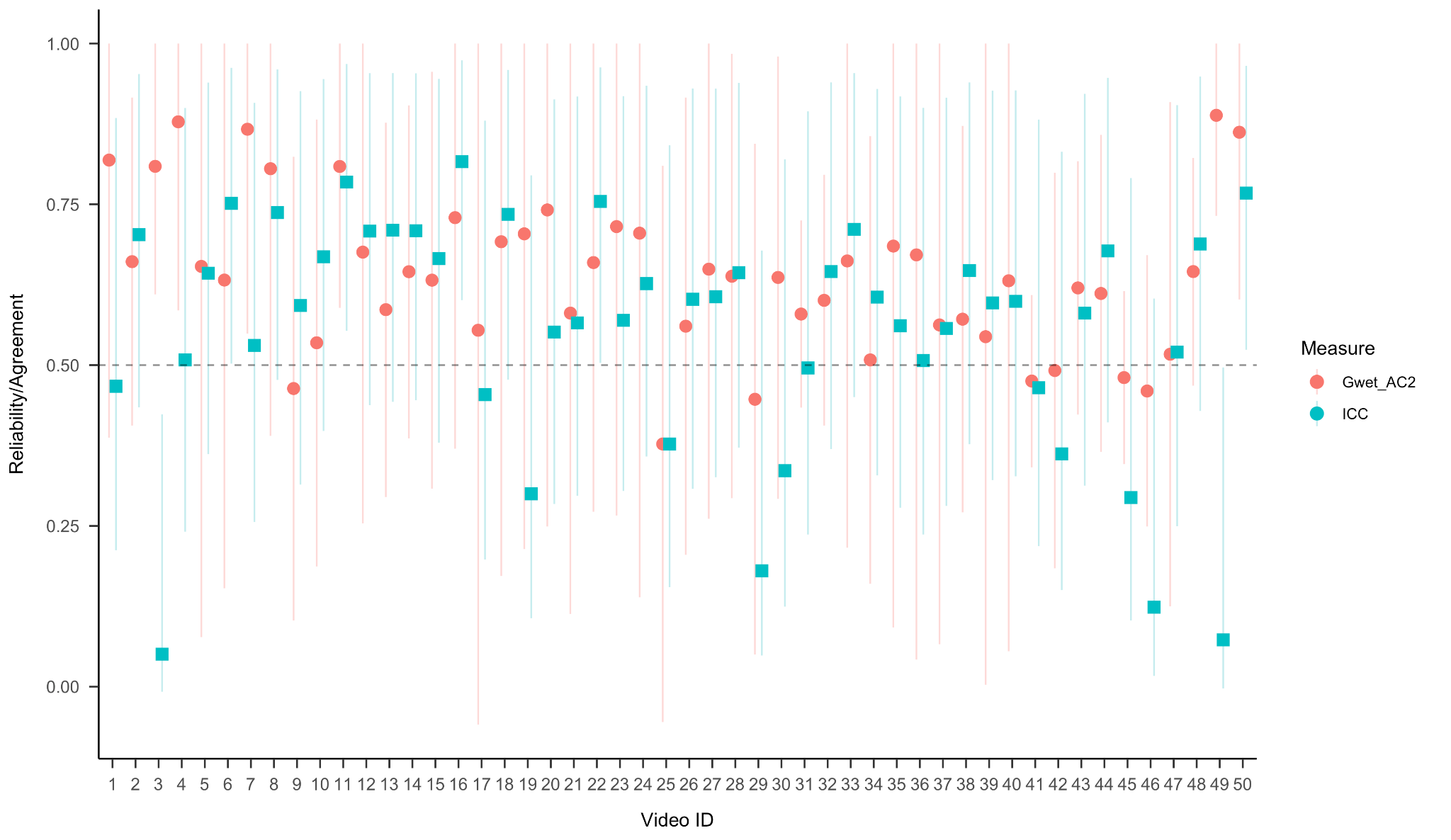


Actor ID = 10


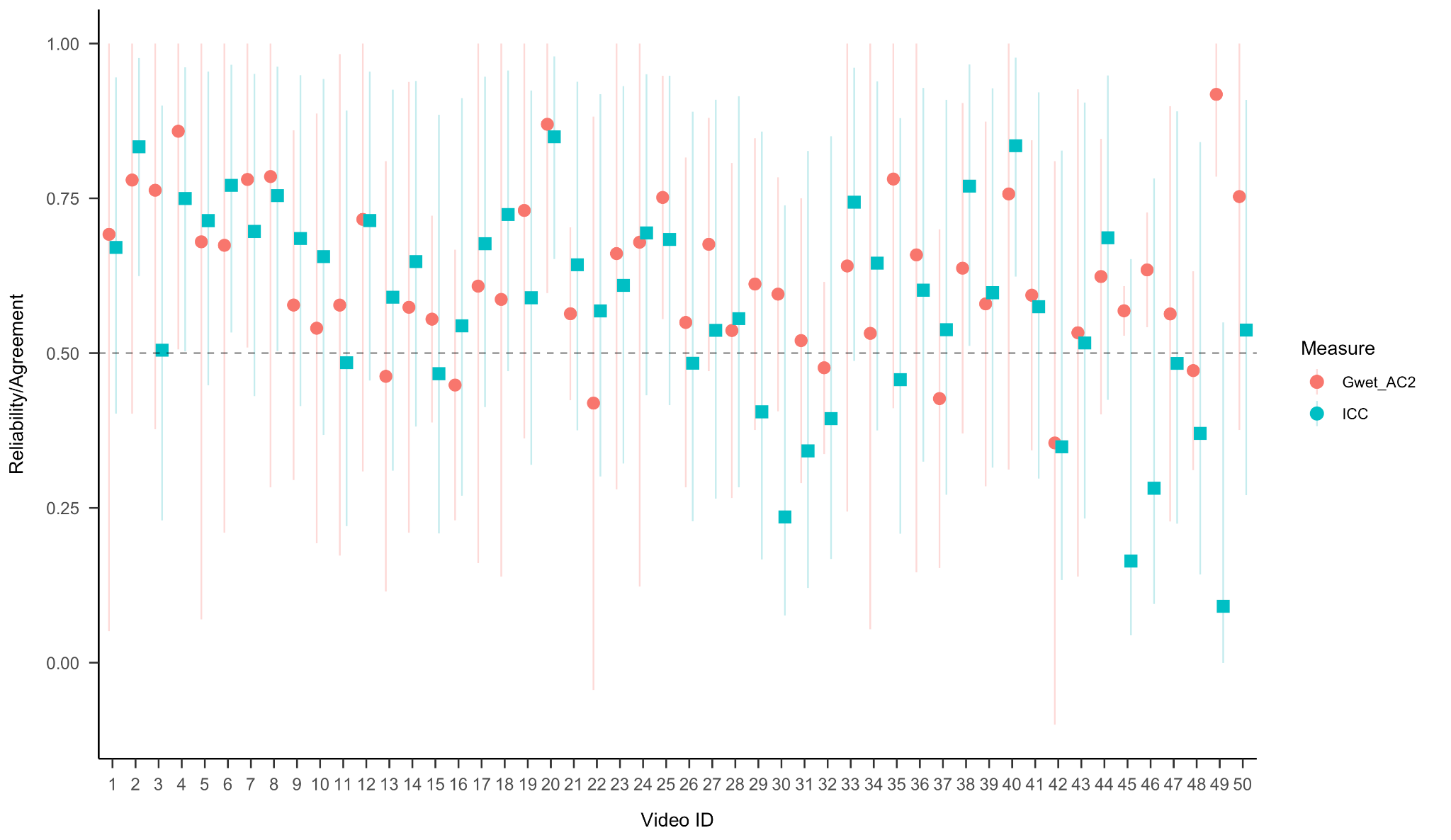


**Supplementary Figure 8**

*Examples of Inter-Rater Reliability for Six Videos (Actor_ID:Video_ID) Showing High Reliability (top row), High Agreement but Low ICC (middle row), and Poor Reliability (bottom row).*


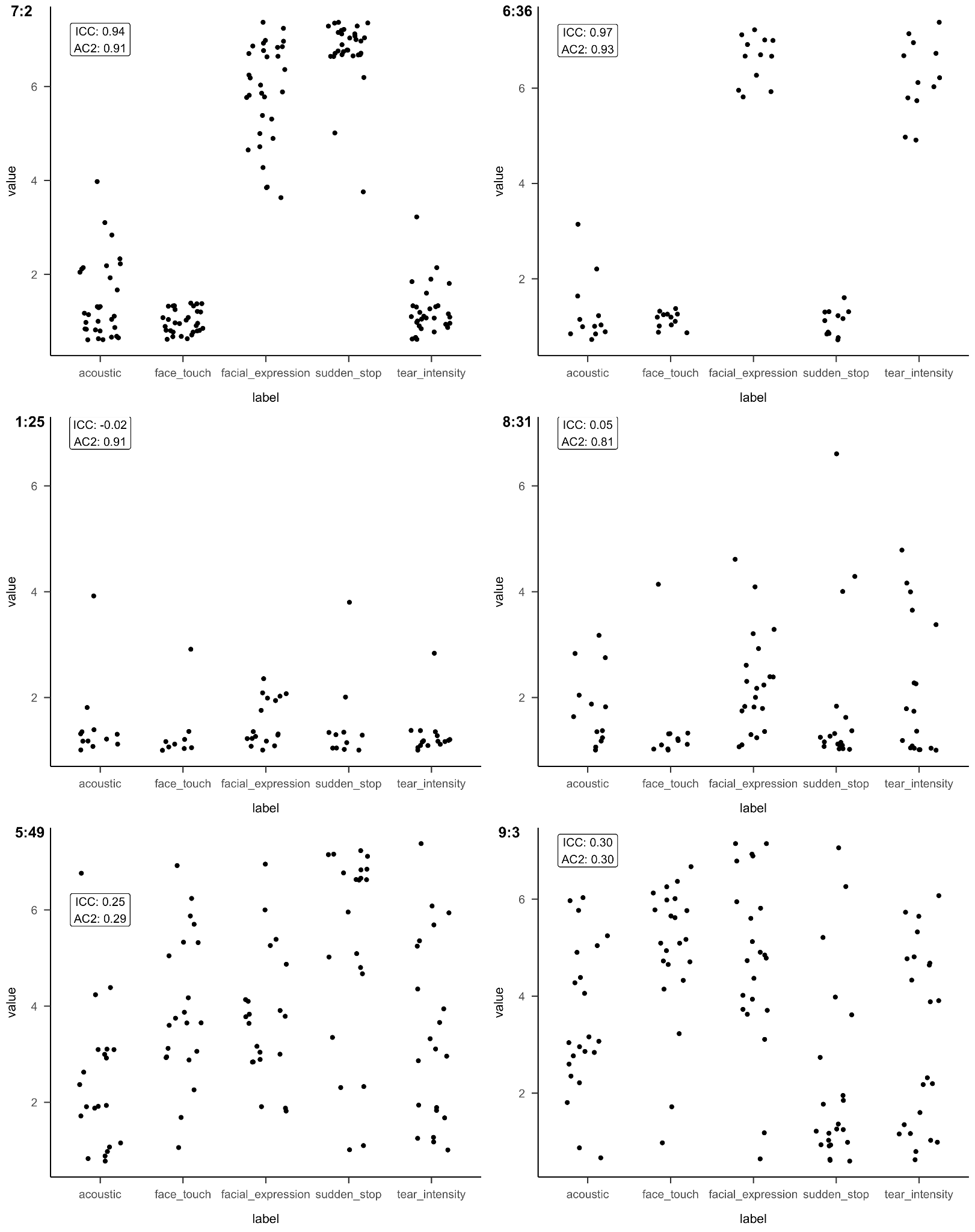


**3.4 Study 1 - Hierarchical Clustering**

Additionally, we performed hierarchical clustering of the average ratings per video (across actors). In order to determine the number of clusters, we employed the *NBClust* command running 23 indices to determine the number of clusters. The majority of tests proposed 3 or 7 clusters as the best number of clusters. An overview of the dendrogram is presented in Supplementary Figure 9. The three clusters differentiated between videos including 1) face touching, 2) baseline/natural expression, 3) no face touching. Further, the face touching cluster differentiated between 1) face touching and gradual stopping and 2) face touching and sudden stopping. The no face touching cluster differentiated between 4) no face touching, loud vocalizations, and gradual stopping, 5) no face touching, silent vocalizations, and gradual stopping, 6) no tears, no face touching, loud vocalizations, and 7) no face touching and sudden stopping. Interestingly, none of the clusters differentiated between the intensity of tears.

**Supplementary Figure 9**

*Dendrogram of Perception Ratings for the 50 Videos (across Actors). Higher Order Clusters (3) and Lower Order Clusters (7) are Marked with Colored Rectangles*


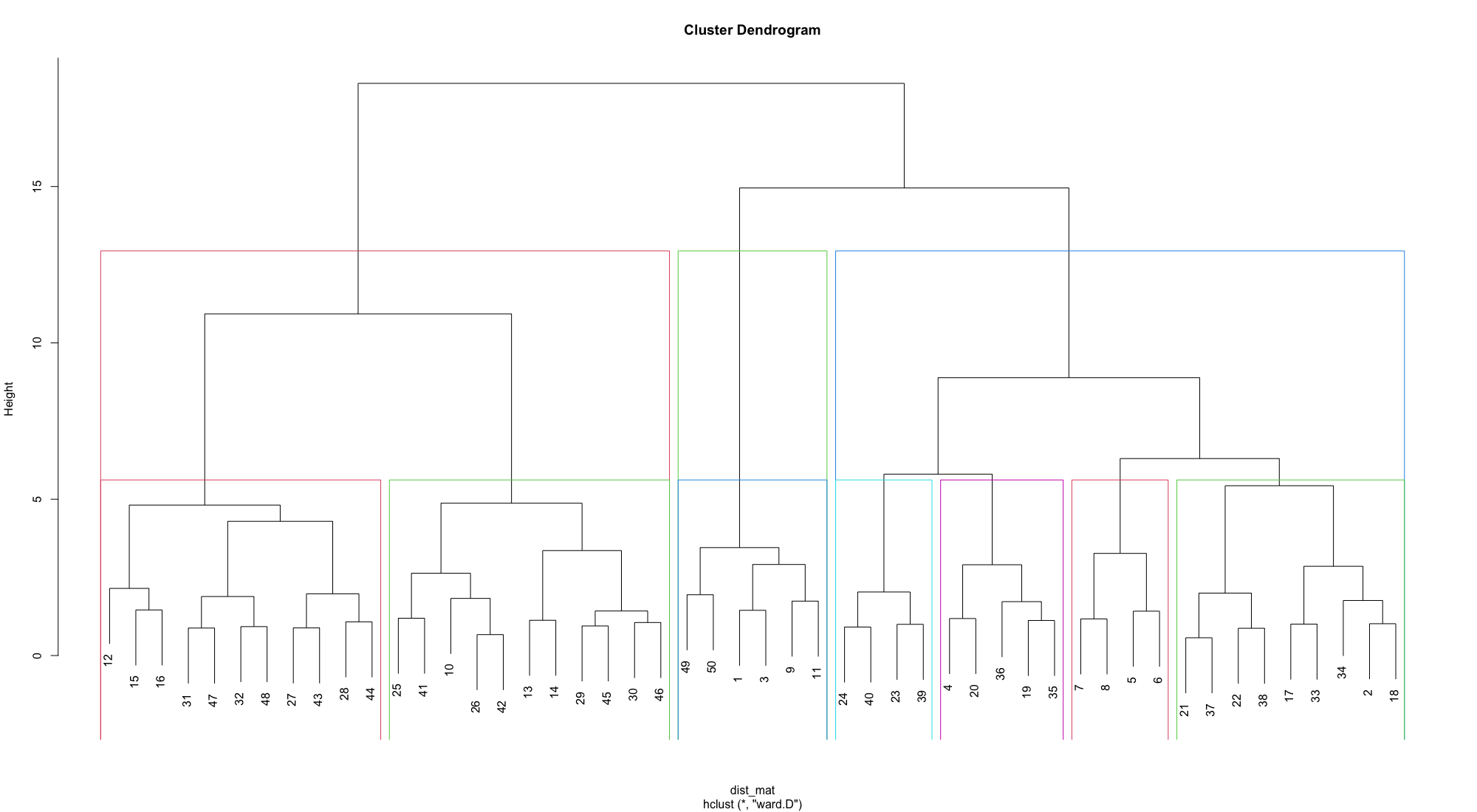


**Section 4 – Video Validation (Study 2)**

**4.1 Sample Overview Study 2**

**Supplementary Table 8**

*Overview of Sample Characteristics across the Four Countries in Study 2*

| Country | ZA | CA | PL | SCAND* |
| --- | --- | --- | --- | --- |
| Sampled | 417 | 410 | 409 | 456 |
| *Exclusions* | | | | |
| Complete Missing (including comprehension failure) | 23 | 8 | 11 | 16 |
| Video Comprehension Check Excluded | 10 | 2 | 0 | 2 |
| Attention Check Excluded | 0 | 0 | 1 | 0 |
| Time Excluded | 0 (27) | 1 (65) | 1 (97) | 2 (82) |
| *Final Sample* | | | | |
| *N* | 394 | 401 | 396 | 438 |
| *N*_male_ | 193 | 193 | 187 | 203 |
| *N*_female_ | 199 | 198 | 196 | 226 |
| *N*_nonbinary_ | 1 | 6 | 11 | 9 |
| *N*_other_ | - | 2 | 2 | - |
| Age | 29.2 (7.98) | 34.6 (11.4) | 27 (7.52) | 31.7 (8.97) |
|  | 18-67 | 18-73 | 18-61 | 18-70 |
| *N*_observations_ | 1549 | 1543 | 1491 | 1668 |
| *Dark Triad* | | | | |
| Psychopathy | 0.77 | 0.77 | 0.79 | 0.65 |
| Narcissism | 0.84 | 0.84 | 0.86 | 0.81 |
| Machiavellianism | 0.86 | 0.84 | 0.89 | 0.82 |

*Note.* ZA = South Africa, CA = Canada, PL = Poland, SCAND = Scandinavian. *Scandinavian: *n* = 247 Swedish, *n* = 31 Norwegian, *n* = 31 Danish.

**4.2 Study 2 – Multilevel Models**

**Supplementary Table 9**

*Overview of Multilevel Model with Perceived Sadness Expression as the Outcome Variable and Tear Intensity (No Tears vs. Subtle Tears vs. Intense Tears) as the Predictor. Random Intercepts Based on Participants Nested in Sample (Country) and Videos Nested in Actors. Left Side Shows the originally Registered Model and the Right Side Shows the Model with Robust Estimation Using the robustlmm Package*

|  | **Perceived Sadness Expression**  **[Registered Model]** | | | | | **Perceived Sadness Expression**  **[Robust Model]** | | | | |
| --- | --- | --- | --- | --- | --- | --- | --- | --- | --- | --- |
| *Predictors* | *Estimates* | *std. Beta* | *CI* | *standardized CI* | *p* | *Estimates* | *std. Beta* | *CI* | *standardized CI* | *p* |
| (Intercept) | 3.62 | -0.05 | 3.19 – 4.05 | -0.27 – 0.17 | **<0.001** | 3.59 | -0.06 | 3.02 – 4.16 | -0.35 – 0.22 | **<0.001** |
| Tear Intensity [no tears vs. tears] | 1.00 | 0.51 | 0.77 – 1.24 | 0.39 – 0.63 | **<0.001** | 1.03 | 0.52 | 0.77 – 1.30 | 0.39 – 0.65 | **<0.001** |
| Tear Intensity [subtle vs. intense] | 0.34 | 0.17 | 0.15 – 0.53 | 0.08 – 0.27 | **<0.001** | 0.33 | 0.16 | 0.11 – 0.54 | 0.06 – 0.27 | **0.003** |
| **Random Effects** | | | | | | | | | | |
| σ^2^ | 2.30 | | | | | 2.59 | | | | |
| τ_00_ | 0.83 _ID:Sample_ | | | | | 0.84 _ID:Sample_ | | | | |
|  | 0.52 _Video_ID:Actor_ID_ | | | | | 0.63 _Video_ID:Actor_ID_ | | | | |
|  | 0.12 _Actor_ID_ | | | | | 0.16 _Actor_ID_ | | | | |
|  | 0.14 _Sample_ | | | | | 0.24 _Sample_ | | | | |
| ICC | 0.41 | | | | | 0.42 | | | | |
| N | 50 _Video_ID_ | | | | | 50 _Video_ID_ | | | | |
|  | 10 _Actor_ID_ | | | | | 10 _Actor_ID_ | | | | |
|  | 454 _ID_ | | | | | 454 _ID_ | | | | |
|  | 4 _Sample_ | | | | | 4 _Sample_ | | | | |
| Observations | 6260 | | | | | 6260 | | | | |
| Marginal R^2^ / Conditional R^2^ | 0.031 / 0.430 | | | | | 0.028 / 0.436 | | | | |

**Supplementary Table 10**

*Overview of Multilevel Model with Perceived Helplessness as the Outcome Variable and Tear Intensity (No Tears vs. Subtle Tears vs. Intense Tears) as the Predictor. Random Intercepts Based on Participants Nested In Sample (Country) and Videos Nested in Actors. Left Side Shows the originally Registered Model and the Right Side Shows the Model with Robust Estimation Using the robustlmm Package*

|  | **Perceived Helplessness**  **[Registered Model]** | | | | | **Perceived Helplessness**  **[Robust Model]** | | | | |
| --- | --- | --- | --- | --- | --- | --- | --- | --- | --- | --- |
| *Predictors* | *Estimates* | *std. Beta* | *CI* | *standardized CI* | *p* | *Estimates* | *std. Beta* | *CI* | *standardized CI* | *p* |
| (Intercept) | 3.25 | -0.03 | 2.89 – 3.62 | -0.23 – 0.16 | **<0.001** | 3.19 | -0.07 | 2.72 – 3.65 | -0.32 – 0.18 | **<0.001** |
| Tear Intensity [1] | 0.76 | 0.41 | 0.53 – 0.99 | 0.28 – 0.53 | **<0.001** | 0.78 | 0.41 | 0.53 – 1.03 | 0.28 – 0.55 | **<0.001** |
| Tear Intensity [2] | 0.29 | 0.15 | 0.11 – 0.48 | 0.06 – 0.25 | **0.002** | 0.28 | 0.15 | 0.08 – 0.48 | 0.04 – 0.26 | **0.006** |
| **Random Effects** | | | | | | | | | | |
| σ^2^ | 2.15 | | | | | 2.33 | | | | |
| τ_00_ | 0.73 _ID:Sample_ | | | | | 0.67 _ID:Sample_ | | | | |
|  | 0.49 _Video_ID:Actor_ID_ | | | | | 0.57 _Video_ID:Actor_ID_ | | | | |
|  | 0.07 _Actor_ID_ | | | | | 0.10 _Actor_ID_ | | | | |
|  | 0.10 _Sample_ | | | | | 0.17 _Sample_ | | | | |
| ICC | 0.39 | | | | | 0.39 | | | | |
| N | 50 _Video_ID_ | | | | | 50 _Video_ID_ | | | | |
|  | 10 _Actor_ID_ | | | | | 10 _Actor_ID_ | | | | |
|  | 454 _ID_ | | | | | 454 _ID_ | | | | |
|  | 4 _Sample_ | | | | | 4 _Sample_ | | | | |
| Observations | 6259 | | | | | 6259 | | | | |
| Marginal R^2^ / Conditional R^2^ | 0.021 / 0.406 | | | | | 0.019 / 0.405 | | | | |

**Supplementary Table 11**

*Overview Of Multilevel Model with Perceived Overall Expression Intensity as the Outcome Variable and Facial Expression Intensity (Natural vs. Exaggerated) as the Predictor. Random Intercepts Based on Participants Nested in Sample (Country) And Videos Nested In Actors. Left Side Shows the originally Registered Model and the Right Side Shows the Model with Robust Estimation Using the robustlmm Package*

|  | **Perceived Overall Expression Intensity**  **[Registered Model]** | | | | | **Perceived Overall Expression Intensity**  **[Robust Model]** | | | | |
| --- | --- | --- | --- | --- | --- | --- | --- | --- | --- | --- |
| *Predictors* | *Estimates* | *std. Beta* | *CI* | *standardized CI* | *p* | *Estimates* | *std. Beta* | *CI* | *standardized CI* | *p* |
| (Intercept) | 3.66 | -0.06 | 3.24 – 4.09 | -0.29 – 0.17 | **<0.001** | 3.65 | -0.06 | 3.10 – 4.19 | -0.36 – 0.23 | **<0.001** |
| Facial Expression Intensity (natural vs. exaggerated) | 0.21 | 0.11 | 0.07 – 0.35 | 0.04 – 0.19 | **0.003** | 0.23 | 0.12 | 0.08 – 0.37 | 0.04 – 0.20 | **0.003** |
| **Random Effects** | | | | | | | | | | |
| σ^2^ | 2.03 | | | | | 2.15 | | | | |
| τ_00_ | 0.77 _ID:Sample_ | | | | | 0.84 _ID:Sample_ | | | | |
|  | 0.43 _Video_ID:Actor_ID_ | | | | | 0.46 _Video_ID:Actor_ID_ | | | | |
|  | 0.12 _Actor_ID_ | | | | | 0.14 _Actor_ID_ | | | | |
|  | 0.13 _Sample_ | | | | | 0.22 _Sample_ | | | | |
| ICC | 0.42 | | | | | 0.44 | | | | |
| N | 50 _Video_ID_ | | | | | 50 _Video_ID_ | | | | |
|  | 10 _Actor_ID_ | | | | | 10 _Actor_ID_ | | | | |
|  | 454 _ID_ | | | | | 454 _ID_ | | | | |
|  | 4 _Sample_ | | | | | 4 _Sample_ | | | | |
| Observations | 6260 | | | | | 6260 | | | | |
| Marginal R^2^ / Conditional R^2^ | 0.003 / 0.419 | | | | | 0.003 / 0.439 | | | | |

**Section 5 – Picture Validation Round 1 (Study 3a)**

***Method***

**Participants.** We registered to obtain at least 20 ratings per picture. Since the total pool consisted of 70 pictures and each participant rated 5 pictures, around 280 participants would be needed. To account for possible exclusions and random distribution we registered recruiting 300 participants.

A total of 301 UK-based participants were sampled via Prolific.com for a 6-minute study with £0.75 as compensation. None of the participants was excluded based on the registered exclusion criteria. The final sample size included 301 participants (148 males, 149 females, 3 non-binary, 1 other) ranging from 18 to 77 years-of-age (*M* = 41.1, *SD* = 13.6). The majority (*n* = 269) indicated a UK nationality. On average 21.5 (*SD* = 5.23) ratings were obtained per picture (9 to 34 ratings per picture; 3 pictures with less than 15 ratings). In total, the dataset included 1505 ratings.

**Design and Procedure.** We employed a 10 (actor) x 3 (tears: no tears vs. subtle tears vs. intense tears) x 2 (expression: natural vs. exaggerated) mixed design. This resulted in 60 possible combinations, and we also included one control picture per actor, thereby having 70 possible combinations and pictures in total.

**Picture Stimuli.** Pictures were taken from the video stimuli, which were described earlier. The pictures included 10 professional Polish actors (5 women, 5 men) who signed consent that their videos could be used and distributed for scientific research purposes. Actors were recorded from shoulders upwards, looking directly at the camera. All the actors wore white T-shirts and were filmed against a light grey backdrop. T-shirts were replaced if they got wet from crying. Actors originally posed five different types of behavior (see video validation). Based on the dynamic nature of some of them, we only focused on the intensity of tears (no tears, subtle tears, intense tears) and the type of facial expression (natural, exaggerated). Tears were simulated by using water droplets in different intensities applied to the eye. The amount of tears needed (40 videos with some form of tears per actor, including possible repetitions) made it impossible to elicit tears via emotional sources (e.g., watching a sad movie) or employing menthol tear sticks.

After providing informed consent, participants were presented with instructions. Participants were shown 5 pictures, randomly chosen 5 of the 10 actors, and for each actor, it was randomly determined which combination of factors would be presented. After the last picture, participants were presented with an attention check (“Please select ‘3’ on the scale”). Finally, participants completed information regarding their gender, age, and nationality.

**Measures.**  For each video participants completed the same 13 items in fixed order.

***Validation Items.*** First, participants completed validation measures asking about the intensity of tears (“This person… is not shedding tears at all (1) / shedding a lot of tears (7)), facial expression (This person… has a very subtle facial expression (1) / has an exaggerated facial expression (7)).

***Expression Related.*** Then participants completed four items regarding the specific expression, focusing on the authenticity (“How authentic do you think the expression of this person is?”), intensity (“How intense do you think the expression of this person is?”), appropriateness (“How appropriate do you think the expression of this person is?”), sadness (“How much sadness does this person express?”) of the expression.

***Inferences.*** In addition, participants rated how warm (“How warm does this person appear to you?”), helpless (“How helpless does this person appear to you?”), manipulative (“How manipulative does this person appear to you?”), and honest (“How honest does this person appear to you?”) they perceived the person.

***Felt Emotions.*** Participants also rated how compassionate (“When seeing this person, I feel compassionate.”) and distressed (“When seeing this person, I feel upset”) they felt when seeing the picture.

**Social Support Intentions.** Finally, participants rated how much support they would offer to the person (“I would offer support to this person”). All items were rated on a 7-point scale from *not at all* (1) to *very much* (7).

***Results***

**Norming Data.** Norming data for all pictures can be accessed at <https://osf.io/udt7h>.

**Picture Validation.** In order to check for the validation of the pictures, we first conducted five multilevel models. The first three models included tear intensity as the predictor (contrast coded: -⅔ no tears, ⅓ subtle tears, ⅓ intense tears; 0 no tears; -.5 subtle tears, .5 intense tears) and perceived tear intensity, perceived expressed sadness, and perceived helplessness as outcomes. The other two models included facial expression as the predictor (natural vs. exaggerated) and perceived facial expression and perceived expression intensity as outcomes. For all models, we added participants as random effects and pictures nested in actors. An overview of ratings is presented in Supplementary Figure 10 and models are presented in Supplementary Table 12-16. For tear intensity, we observed that pictures that should show *subtle tears* (*M* = 4.78, *SE* = 0.15) and *intense tears* (*M* = 5.80, *SE* = 0.15) were rated as higher in tear intensity than pictures that should show no tears (*M* = 2.08, *d* = 1.48 [1.38, 1.58], *t*(55.21) = 28.99, *p* < 0.001). Similarly, pictures that should show *intense tears* were rated as higher in tear intensity than subtle tear pictures (*d* = 0.47 [0.34, 0.61], *t*(56.19) = 7.03, *p* < 0.001). Pictures including subtle (*M* = 4.87, *SE* = 0.18) or intense tears (*M* = 5.26, *SE* = 0.18) were rated as expressing more sadness than pictures with no tears (*M* = 3.85, *SE* = 0.17, *d* = 0.73 [0.57, 0.89], *t*(57.47) = 8.87, *p* < 0.001). At the same time, intense tears were perceived as expressing slightly more sadness than subtle tear pictures (*d* = 0.23 [0.02, 0.45], *t*(58.29) = 2.16, *p* = 0.035). We also observed that actors with subtle (*M* = 4.38, *SE* = 0.16) or intense tears (*M* = 4.68, *SE* = 0.16) were rated as more helpless than actors with no tears (*M* = 3.40, *SE* = 0.14, *d* = 0.68 [0.53, 0.82], *t*(57.89) = 8.88, *p* < 0.001). At the same time, actors with intense tears were not rated as significantly more helpless than actors with subtle tears (*d* = 0.18 [-0.01, 0.38], *t*(58.84) = 1.83, *p* = 0.067).

We also observed that videos including actors showing an exaggerated facial expression were rated higher in facial expression intensity (*M* = 5.70, *SE* = 0.18) than participants instructed to show a natural facial expression (*M* = 4.05, *SE* = 0.16; *d* = 0.88 [0.63, 1.12], *t*(58.33) = 7.01, *p* < 0.001). Similarly, the expression of actors showing an exaggerated facial expression was perceived as more intense (*M* = 4.98, *SE* = 0.17) compared to actors showing a natural expression (*M* = 4.19, *SE* = 0.16, *d* = 0.50 [0.29, 0.71], *t*(58.59) = 4.74, *p* < 0.001).

**Supplementary Table 12**

*Overview of Multilevel Model with Perceived Tear Intensity as the Outcome Variable and Tear Intensity (No Tears vs. Subtle Tears vs. Intense Tears) as the Predictor. Random Intercepts Based on Participants and Pictures Nested in Actors. Left Side Shows the originally Registered Model and the Right Side Shows the Model with Robust Estimation Using the robustlmm Package*

|  | **Perceived Tear Intensity**  **[Registered Model]** | | | | | **Perceived Tear Intensity**  **[Robust Model]** | | | | |
| --- | --- | --- | --- | --- | --- | --- | --- | --- | --- | --- |
| *Predictors* | *Estimates* | *std. Beta* | *CI* | *standardized CI* | *p* | *Estimates* | *std. Beta* | *CI* | *standardized CI* | *p* |
| (Intercept) | 4.22 | 0.15 | 3.98 – 4.46 | 0.04 – 0.26 | **<0.001** | 4.22 | 0.15 | 3.97 – 4.47 | 0.03 – 0.26 | **<0.001** |
| Tear Intensity [no tears vs. tears] | 3.21 | 1.48 | 2.99 – 3.43 | 1.38 – 1.58 | **<0.001** | 3.46 | 1.60 | 3.29 – 3.63 | 1.52 – 1.68 | **<0.001** |
| Tear Intensity [subtle vs. intense] | 1.03 | 0.47 | 0.74 – 1.31 | 0.34 – 0.61 | **<0.001** | 1.09 | 0.50 | 0.86 – 1.31 | 0.40 – 0.60 | **<0.001** |
| **Random Effects** | | | | | | | | | | |
| σ^2^ | 1.46 | | | | | 1.11 | | | | |
| τ_00_ | 0.53 _ID_ | | | | | 0.35 _ID_ | | | | |
|  | 0.13 _PIC_ID:Actor_ID_ | | | | | 0.06 _PIC_ID:Actor_ID_ | | | | |
|  | 0.10 _Actor_ID_ | | | | | 0.13 _Actor_ID_ | | | | |
| ICC | 0.34 | | | | | 0.33 | | | | |
| N | 301 _ID_ | | | | | 301 _ID_ | | | | |
|  | 70 _PIC_ID_ | | | | | 70 _PIC_ID_ | | | | |
|  | 10 _Actor_ID_ | | | | | 10 _Actor_ID_ | | | | |
| Observations | 1505 | | | | | 1505 | | | | |
| Marginal R^2^ / Conditional R^2^ | 0.550 / 0.704 | | | | | 0.655 / 0.768 | | | | |

**Supplementary Table 13**

*Overview of Multilevel Model with Perceived Sadness Expression as the Outcome Variable and Tear Intensity (No Tears vs. Subtle Tears vs. Intense Tears) as the Predictor. Random Intercepts Based on Participants and Pictures Nested in Actors. Left Side Shows the originally Registered Model and the Right Side Shows the Model with Robust Estimation Using the robustlmm Package.*

|  | **Perceived Sadness Expression**  **[Registered Model]** | | | | | **Perceived Sadness Expression**  **[Robust Model]** | | | | |
| --- | --- | --- | --- | --- | --- | --- | --- | --- | --- | --- |
| *Predictors* | *Estimates* | *std. Beta* | *CI* | *standardized CI* | *p* | *Estimates* | *std. Beta* | *CI* | *standardized CI* | *p* |
| (Intercept) | 4.66 | 0.07 | 4.37 – 4.95 | -0.11 – 0.24 | **<0.001** | 4.73 | 0.11 | 4.36 – 5.10 | -0.11 – 0.33 | **<0.001** |
| Tear Intensity [no tears vs. tears] | 1.21 | 0.73 | 0.94 – 1.48 | 0.57 – 0.89 | **<0.001** | 1.18 | 0.71 | 0.95 – 1.41 | 0.57 – 0.85 | **<0.001** |
| Tear Intensity [subtle vs. intense] | 0.39 | 0.23 | 0.04 – 0.74 | 0.02 – 0.45 | **0.031** | 0.39 | 0.23 | 0.08 – 0.69 | 0.05 – 0.42 | **0.012** |
| **Random Effects** | | | | | | | | | | |
| σ^2^ | 1.67 | | | | | 1.76 | | | | |
| τ_00_ | 0.41 _ID_ | | | | | 0.36 _ID_ | | | | |
|  | 0.23 _PIC_ID:Actor_ID_ | | | | | 0.13 _PIC_ID:Actor_ID_ | | | | |
|  | 0.17 _Actor_ID_ | | | | | 0.29 _Actor_ID_ | | | | |
| ICC | 0.33 | | | | | 0.31 | | | | |
| N | 301 _ID_ | | | | | 301 _ID_ | | | | |
|  | 70 _PIC_ID_ | | | | | 70 _PIC_ID_ | | | | |
|  | 10 _Actor_ID_ | | | | | 10 _Actor_ID_ | | | | |
| Observations | 1505 | | | | | 1505 | | | | |
| Marginal R^2^ / Conditional R^2^ | 0.134 / 0.416 | | | | | 0.126 / 0.395 | | | | |

**Supplementary Table 14**

*Overview of Multilevel Model with Perceived Helplessness as the Outcome Variable and Tear Intensity (No Tears vs. Subtle Tears vs. Intense Tears) as the Predictor. Random Intercepts Based on Participants and Pictures Nested in Actors. Left Side Shows the originally Registered Model and the Right Side Shows the Model with Robust Estimation Using the robustlmm Package*

|  | **Perceived Helplessness**  **[Registered Model]** | | | | | **Perceived Helplessness**  **[Robust Model]** | | | | |
| --- | --- | --- | --- | --- | --- | --- | --- | --- | --- | --- |
| *Predictors* | *Estimates* | *std. Beta* | *CI* | *standardized CI* | *p* | *Estimates* | *std. Beta* | *CI* | *standardized CI* | *p* |
| (Intercept) | 4.15 | 0.07 | 3.91 – 4.40 | -0.08 – 0.22 | **<0.001** | 4.20 | 0.10 | 3.91 – 4.49 | -0.08 – 0.27 | **<0.001** |
| Tear Intensity [no tears vs. tears] | 1.13 | 0.68 | 0.88 – 1.38 | 0.53 – 0.82 | **<0.001** | 1.14 | 0.68 | 0.89 – 1.39 | 0.53 – 0.83 | **<0.001** |
| Tear Intensity [subtle vs. intense] | 0.31 | 0.18 | -0.02 – 0.63 | -0.01 – 0.38 | 0.067 | 0.31 | 0.19 | -0.02 – 0.64 | -0.01 – 0.39 | 0.065 |
| **Random Effects** | | | | | | | | | | |
| σ^2^ | 1.76 | | | | | 1.82 | | | | |
| τ_00_ | 0.52 _ID_ | | | | | 0.52 _ID_ | | | | |
|  | 0.18 _PIC_ID:Actor_ID_ | | | | | 0.17 _PIC_ID:Actor_ID_ | | | | |
|  | 0.10 _Actor_ID_ | | | | | 0.15 _Actor_ID_ | | | | |
| ICC | 0.31 | | | | | 0.32 | | | | |
| N | 301 _ID_ | | | | | 301 _ID_ | | | | |
|  | 70 _PIC_ID_ | | | | | 70 _PIC_ID_ | | | | |
|  | 10 _Actor_ID_ | | | | | 10 _Actor_ID_ | | | | |
| Observations | 1505 | | | | | 1505 | | | | |
| Marginal R^2^ / Conditional R^2^ | 0.114 / 0.390 | | | | | 0.112 / 0.394 | | | | |

**Supplementary Table 15**

*Overview of Multilevel Model with Perceived Facial Expression Intensity as the Outcome Variable and Facial Expression Intensity (Natural vs. Exaggerated) as the Predictor. Random Intercepts Based on Participants and Pictures Nested in Actors. Left side shows the originally registered model and the right side shows the model with robust estimation using the robustlmm package.*

|  | **Perceived Facial Expression Intensity**  **[Registered Model]** | | | | | **Perceived Facial Expression Intensity**  **[Robust Model]** | | | | |
| --- | --- | --- | --- | --- | --- | --- | --- | --- | --- | --- |
| *Predictors* | *Estimates* | *std. Beta* | *CI* | *standardized CI* | *p* | *Estimates* | *std. Beta* | *CI* | *standardized CI* | *p* |
| (Intercept) | 4.05 | -0.38 | 3.74 – 4.37 | -0.55 – -0.22 | **<0.001** | 4.28 | -0.26 | 3.99 – 4.58 | -0.42 – -0.10 | **<0.001** |
| Facial Expression Intensity (natural vs. exaggerated) | 1.65 | 0.88 | 1.19 – 2.12 | 0.63 – 1.12 | **<0.001** | 1.55 | 0.82 | 1.11 – 2.00 | 0.59 – 1.06 | **<0.001** |
| **Random Effects** | | | | | | | | | | |
| σ^2^ | 1.84 | | | | | 1.79 | | | | |
| τ_00_ | 0.34 _ID_ | | | | | 0.13 _ID_ | | | | |
|  | 0.85 _PIC_ID:Actor_ID_ | | | | | 0.75 _PIC_ID:Actor_ID_ | | | | |
|  | 0.01 _Actor_ID_ | | | | | 0.00 _Actor_ID_ | | | | |
| ICC | 0.40 | | | | |  | | | | |
| N | 301 _ID_ | | | | | 301 _ID_ | | | | |
|  | 70 _PIC_ID_ | | | | | 70 _PIC_ID_ | | | | |
|  | 10 _Actor_ID_ | | | | | 10 _Actor_ID_ | | | | |
| Observations | 1505 | | | | | 1505 | | | | |
| Marginal R^2^ / Conditional R^2^ | 0.181 / 0.505 | | | | | 0.248 / NA | | | | |

**Supplementary Table 16**

*Overview of Multilevel Model with Perceived Overall Expression Intensity as the Outcome Variable and Facial Expression Intensity (Natural vs. Exaggerated) as the Predictor. Random Intercepts Based on Participants and Pictures Nested in Actors. Left Side Shows the originally Registered Model and the Right Side Shows the Model with Robust Estimation Using the robustlmm Package.*

|  | **Perceived Overall Expression Intensity**  **[Registered Model]** | | | | | **Perceived Overall Expression Intensity**  **[Robust Model]** | | | | |
| --- | --- | --- | --- | --- | --- | --- | --- | --- | --- | --- |
| *Predictors* | *Estimates* | *std. Beta* | *CI* | *standardized CI* | *p* | *Estimates* | *std. Beta* | *CI* | *standardized CI* | *p* |
| (Intercept) | 4.19 | -0.22 | 3.88 – 4.49 | -0.42 – -0.03 | **<0.001** | 4.34 | -0.13 | 4.02 – 4.65 | -0.33 – 0.07 | **<0.001** |
| Facial Expression Intensity (natural vs. exaggerated) | 0.79 | 0.50 | 0.47 – 1.12 | 0.29 – 0.71 | **<0.001** | 0.76 | 0.48 | 0.46 – 1.05 | 0.29 – 0.66 | **<0.001** |
| **Random Effects** | | | | | | | | | | |
| σ^2^ | 1.57 | | | | | 1.64 | | | | |
| τ_00_ | 0.38 _ID_ | | | | | 0.22 _ID_ | | | | |
|  | 0.39 _PIC_ID:Actor_ID_ | | | | | 0.29 _PIC_ID:Actor_ID_ | | | | |
|  | 0.11 _Actor_ID_ | | | | | 0.15 _Actor_ID_ | | | | |
| ICC | 0.36 | | | | | 0.29 | | | | |
| N | 301 _ID_ | | | | | 301 _ID_ | | | | |
|  | 70 _PIC_ID_ | | | | | 70 _PIC_ID_ | | | | |
|  | 10 _Actor_ID_ | | | | | 10 _Actor_ID_ | | | | |
| Observations | 1505 | | | | | 1505 | | | | |
| Marginal R^2^ / Conditional R^2^ | 0.059 / 0.400 | | | | | 0.058 / 0.328 | | | | |

**Supplementary Figure 10**

*Overview of Perception Ratings across All Participants (N = 301, Observations N = 1505) for Five Different Variables: Perceived Tear Intensity, Perceived Expressed Sadness, Perceived Helplessness, Perceived Facial Expression Intensity, Perceived Overall Expression Intensity. White Dots Indicate Mean Scores, and Boxes Indicate Box-Plots. Mean Scores Represent Descriptive Means*


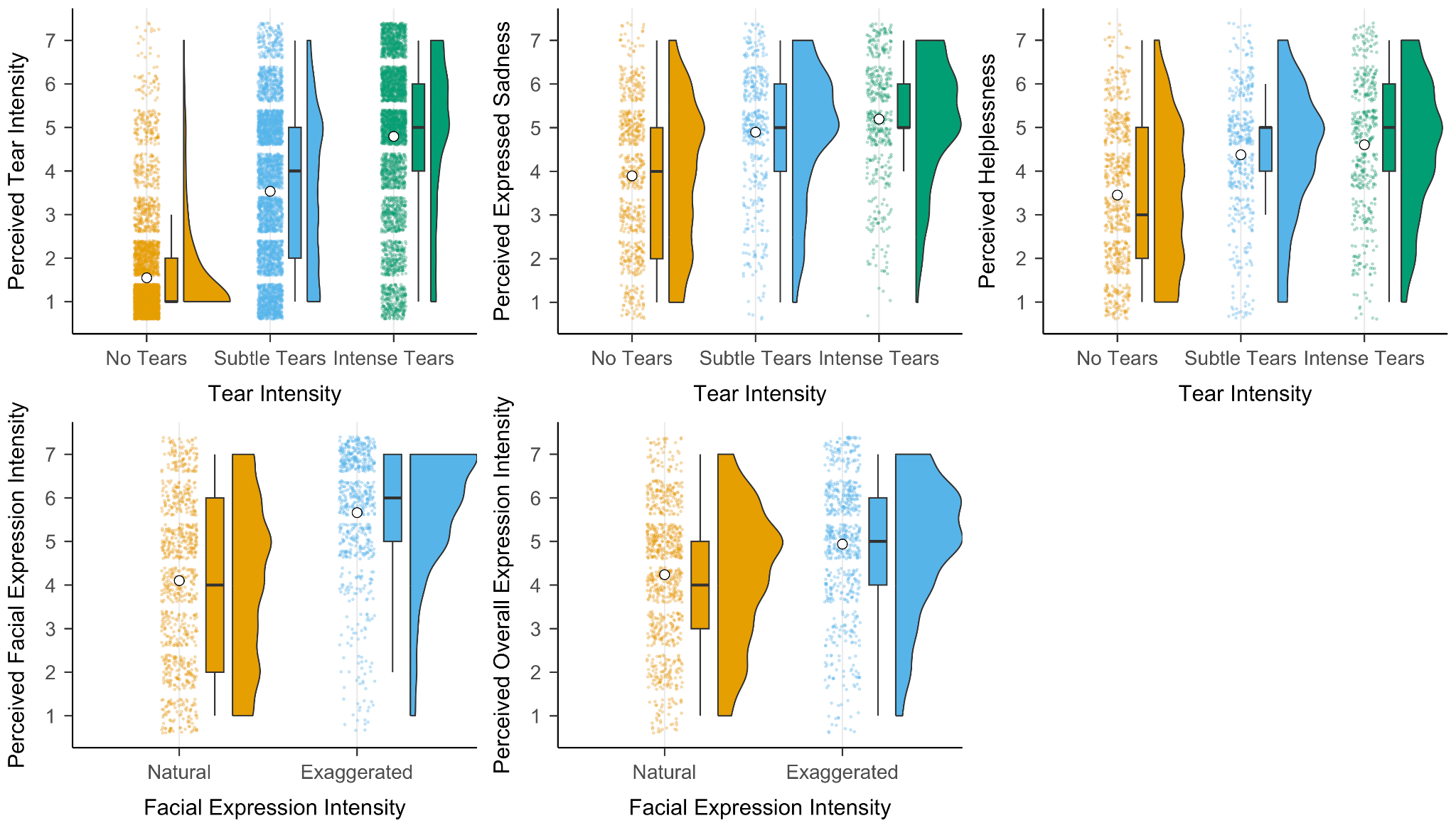


We explored the interaction between tear intensity and facial expression for each of the ratings. We observed a statistically significant interaction between facial expression intensity and tear intensity for the perceived facial expression intensity rating (η2(2) = 8.00, *p* = 0.018). Tears, whether subtle or intense, increased perceived intensity ratings only for natural facial expressions but not for exaggerated facial expressions (Supplementary Figure 11). An overview of ratings for perceived tear intensity and perceived facial expression intensity per picture is provided in Supplementary Figure 12.

**Supplementary Figure 11**

*Overview of Interaction between Tear Intensity Level and Facial Expression Intensity for Ratings of Perceived Facial Expression Intensity Using Estimated Means. Error Bars Represent 95% Confidence Intervals*


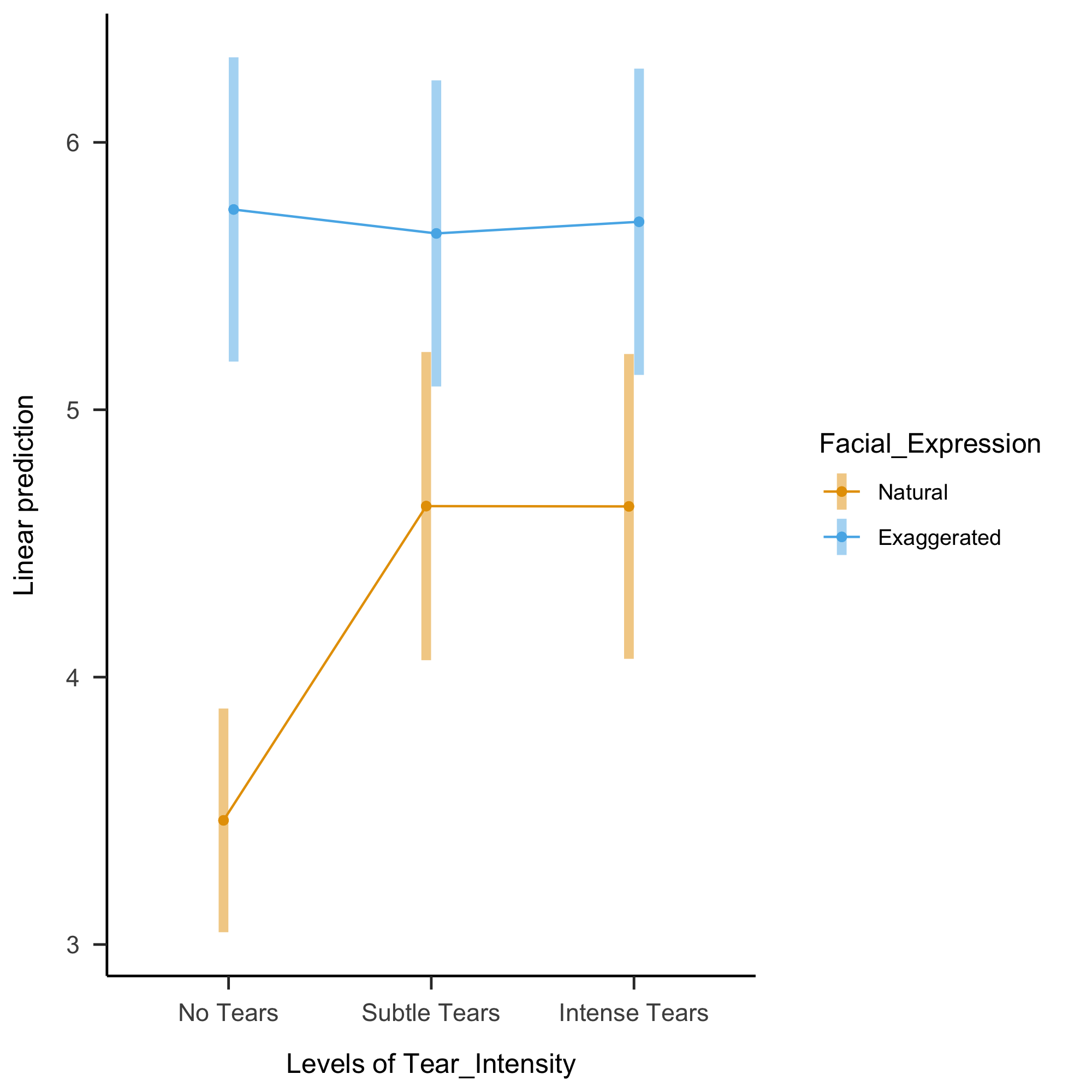


**Supplementary Figure 12**

*Overview of Perception Ratings of Tear Intensity (A), Facial Expression Intensity (B). Dots Represent Means and Lines 95% CIs.*


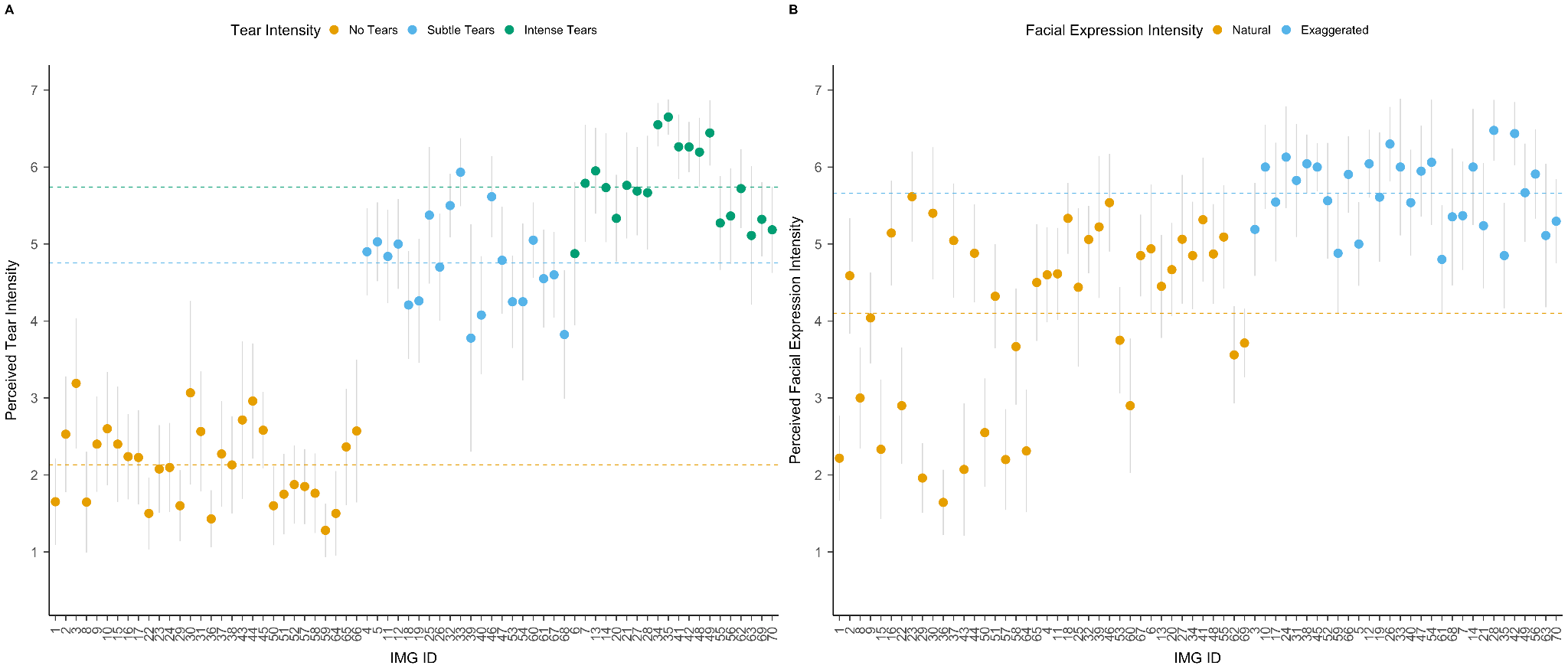


Further, we computed a dichotomous variable for each perception rating indicating the absence (below mean) or presence of the variable (above mean). For tear intensity, we coded subtle tears as matching for a rating between 2 and 4 and intense tears for a rating between 4 and 7 (this was not registered). Again, we observed low matching for facial expression intensity and tear intensity. First, facial expression intensity matched on average in 66.51% of ratings (*SD* = 47.21). Tear intensity on average for 55.61% of ratings (*SD* = 49.70). A detailed overview of matching per video and actor is provided in Supplementary Figure 13. As can be seen, validation was most difficult for subtle tear pictures that showed the lowest match percentage. For this reason, we re-evaluated stimuli with a low matching percentage and attempted to select better-fitting pictures to test them in an additional validation round (Study 3).

**Supplementary Figure 13**

*Radar Plots of Perception Match for Each of The 7 Pictures (Picture IDs from 1 To 7) for the 10 Different Actors. Radar Plots Define Match Percentage between Intended Manipulation and Perception Rating for Tear Intensity (TI) and Facial Expression Intensity (FE). Picture IDs: 1 = Baseline; 2 = No Tears – Natural; 3 = No Tears – Exaggerated; 4 = Subtle Tears – Natural; 5 = Subtle Tears – Exaggerated; 6 = Intense Tears – Natural; 7 = Intense Tears – Exaggerated*


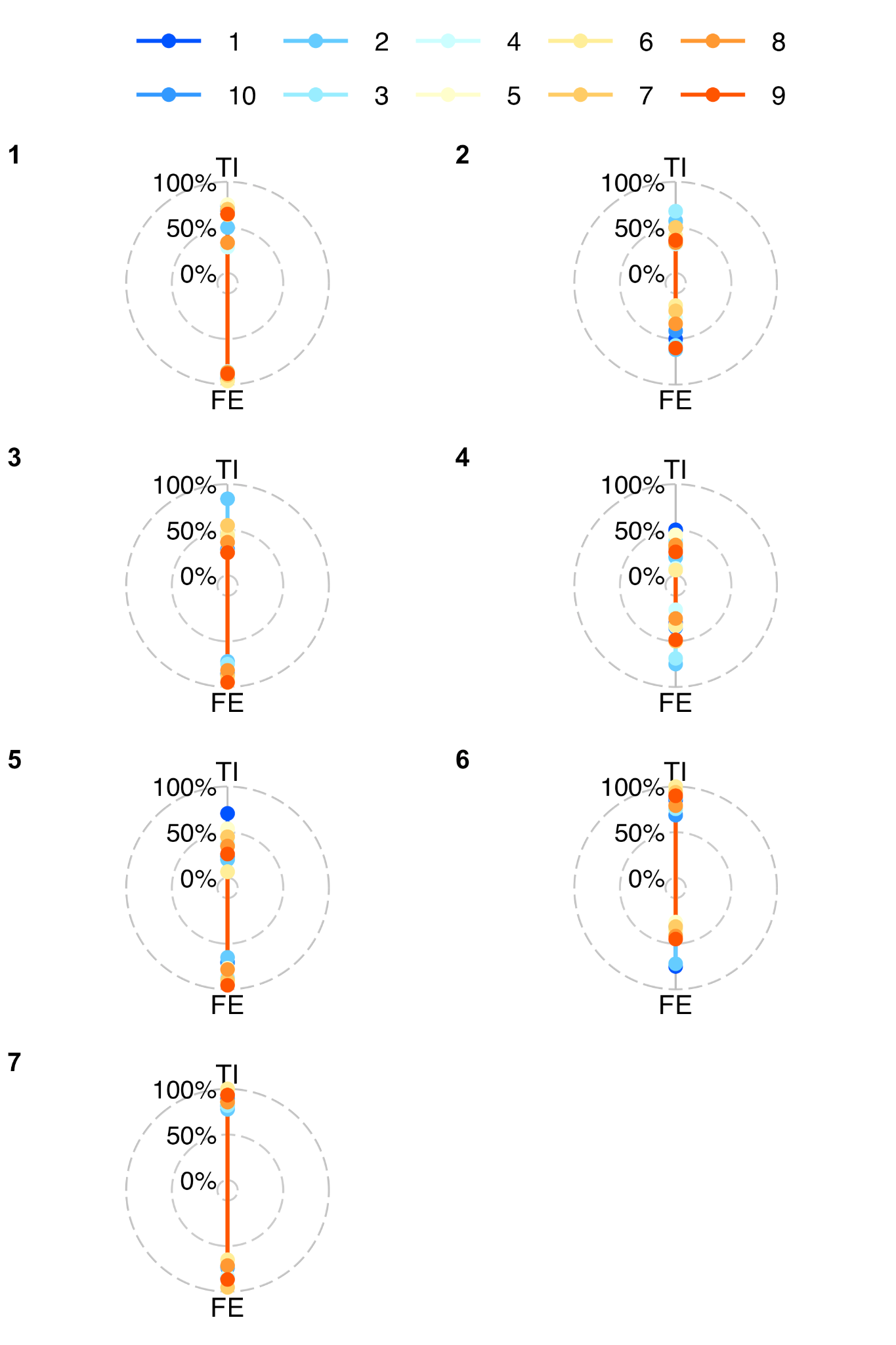


**Section 6 - Picture Validation Round 2 (Study 3b)**

**Supplementary Figure 14**

*Radar Plots of Perception Match for Each of the 7 Pictures (Picture IDs From 1 To 7) for the 10 Different Actors. Radar Plots Define Match Percentage between Intended Manipulation and Perception Rating for Tear Intensity (TI) and Facial Expression Intensity (FE). Picture IDs: 1 = Baseline; 2 = No Tears – Natural; 3 = No Tears – Exaggerated; 4 = Subtle Tears – Natural; 5 = Subtle Tears – Exaggerated; 6 = Intense Tears – Natural; 7 = Intense Tears – Exaggerated*

**
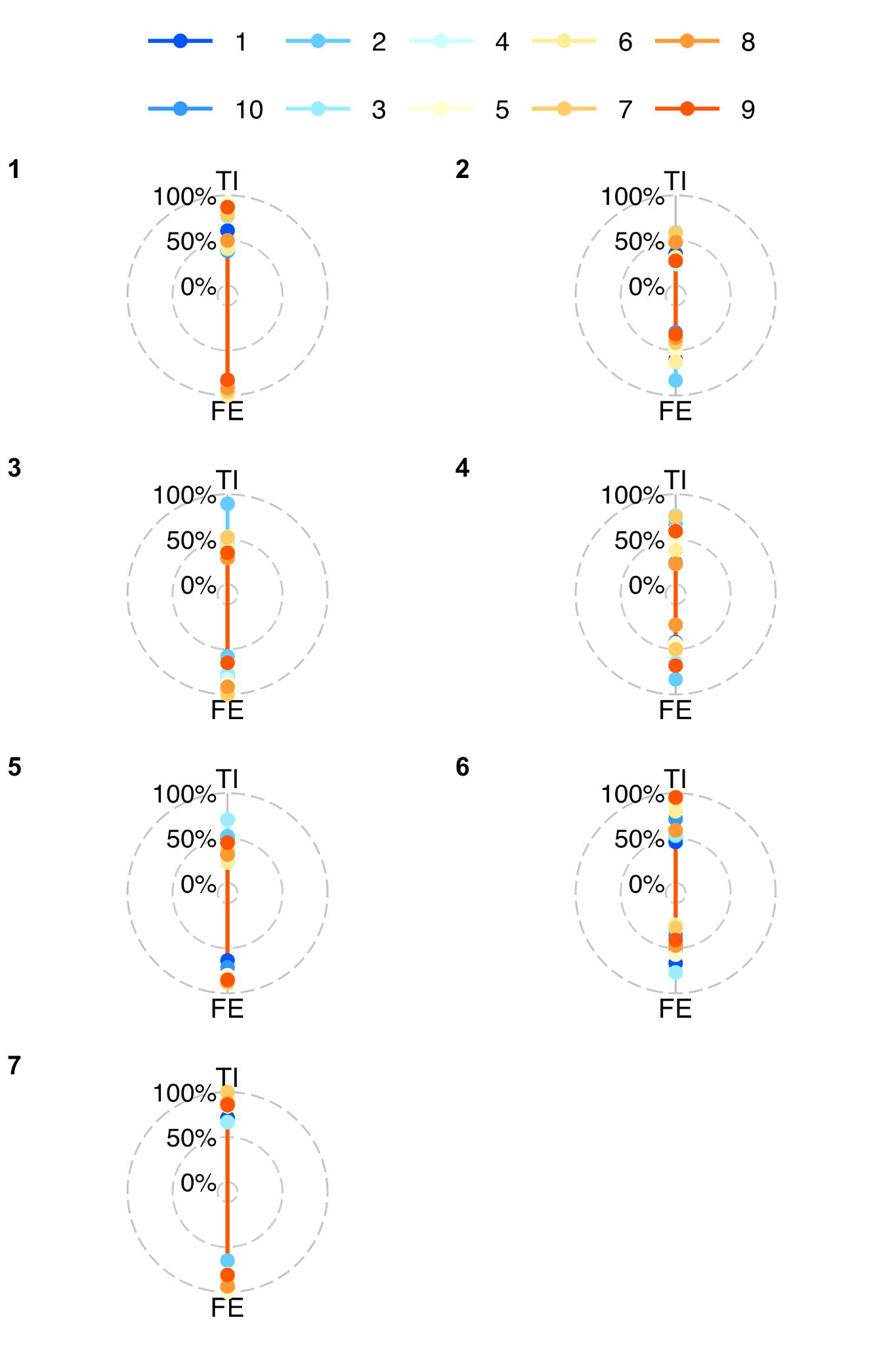
**

**Supplementary Table 17**

*Overview of Multilevel Model with Perceived Tear Intensity as the Outcome Variable and Tear Intensity (No Tears vs. Subtle Tears vs. Intense Tears) as the Predictor. Random Intercepts Based on Participants and Pictures Nested in Actors. Left Side Shows the originally Registered Model and the Right Side Shows the Model with Robust Estimation Using the robustlmm Package*

|  | **Perceived Tear Intensity**  **[Registered Model]** | | | | | **Perceived Tear Intensity**  **[Robust Model]** | | | | |
| --- | --- | --- | --- | --- | --- | --- | --- | --- | --- | --- |
| *Predictors* | *Estimates* | *std. Beta* | *CI* | *standardized CI* | *p* | *Estimates* | *std. Beta* | *CI* | *standardized CI* | *p* |
| (Intercept) | 3.99 | 0.10 | 3.78 – 4.19 | 0.00 – 0.20 | **<0.001** | 3.99 | 0.11 | 3.80 – 4.18 | 0.01 – 0.20 | **<0.001** |
| Tear Intensity [no tears vs. tears] | 2.75 | 1.34 | 2.47 – 3.03 | 1.21 – 1.48 | **<0.001** | 3.01 | 1.47 | 2.75 – 3.28 | 1.34 – 1.60 | **<0.001** |
| Tear Intensity [subtle vs. intense] | 1.27 | 0.62 | 0.91 – 1.63 | 0.44 – 0.80 | **<0.001** | 1.30 | 0.63 | 0.95 – 1.64 | 0.46 – 0.80 | **<0.001** |
| **Random Effects** | | | | | | | | | | |
| σ^2^ | 1.62 | | | | | 1.33 | | | | |
| τ_00_ | 0.39 _ID_ | | | | | 0.28 _ID_ | | | | |
|  | 0.25 _PIC_ID:Actor_ID_ | | | | | 0.23 _PIC_ID:Actor_ID_ | | | | |
|  | 0.05 _Actor_ID_ | | | | | 0.03 _Actor_ID_ | | | | |
| ICC | 0.30 | | | | | 0.29 | | | | |
| N | 300 _ID_ | | | | | 300 _ID_ | | | | |
|  | 70 _PIC_ID_ | | | | | 70 _PIC_ID_ | | | | |
|  | 10 _Actor_ID_ | | | | | 10 _Actor_ID_ | | | | |
| Observations | 1500 | | | | | 1500 | | | | |
| Marginal R^2^ / Conditional R^2^ | 0.472 / 0.630 | | | | | 0.565 / 0.692 | | | | |

**Supplementary Table 18**

*Overview of Multilevel Model with Perceived Sadness Expression as the Outcome Variable and Tear Intensity (No Tears vs. Subtle Tears vs. Intense Tears) as the Predictor. Random Intercepts Based on Participants and Pictures Nested in Actors. Left Side Shows the originally Registered Model and the Right Side Shows the Model with Robust Estimation Using the robustlmm Package*

|  | **Perceived Sadness Expression**  **[Registered Model]** | | | | | **Perceived Sadness Expression**  **[Robust Model]** | | | | |
| --- | --- | --- | --- | --- | --- | --- | --- | --- | --- | --- |
| *Predictors* | *Estimates* | *std. Beta* | *CI* | *standardized CI* | *p* | *Estimates* | *std. Beta* | *CI* | *standardized CI* | *p* |
| (Intercept) | 4.49 | 0.04 | 4.21 – 4.77 | -0.13 – 0.21 | **<0.001** | 4.54 | 0.07 | 4.21 – 4.88 | -0.13 – 0.27 | **<0.001** |
| Tear Intensity [no tears vs. tears] | 0.97 | 0.58 | 0.70 – 1.25 | 0.42 – 0.75 | **<0.001** | 0.99 | 0.59 | 0.72 – 1.26 | 0.43 – 0.76 | **<0.001** |
| Tear Intensity [subtle vs. intense] | 0.50 | 0.30 | 0.14 – 0.85 | 0.08 – 0.51 | **0.007** | 0.52 | 0.31 | 0.17 – 0.88 | 0.10 – 0.53 | **0.004** |
| **Random Effects** | | | | | | | | | | |
| σ^2^ | 1.79 | | | | | 1.92 | | | | |
| τ_00_ | 0.39 _ID_ | | | | | 0.29 _ID_ | | | | |
|  | 0.24 _PIC_ID:Actor_ID_ | | | | | 0.22 _PIC_ID:Actor_ID_ | | | | |
|  | 0.15 _Actor_ID_ | | | | | 0.22 _Actor_ID_ | | | | |
| ICC | 0.30 | | | | | 0.27 | | | | |
| N | 300 _ID_ | | | | | 300 _ID_ | | | | |
|  | 70 _PIC_ID_ | | | | | 70 _PIC_ID_ | | | | |
|  | 10 _Actor_ID_ | | | | | 10 _Actor_ID_ | | | | |
| Observations | 1500 | | | | | 1500 | | | | |
| Marginal R^2^ / Conditional R^2^ | 0.094 / 0.367 | | | | | 0.095 / 0.342 | | | | |

**Supplementary Table 19**

*Overview of Multilevel Model with Perceived Helplessness as the Outcome Variable and Tear Intensity (No Tears vs. Subtle Tears vs. Intense Tears) as the Predictor. Random Intercepts Based on Participants and Pictures Nested in Actors. Left Side Shows the originally Registered Model and the Right Side Shows the Model with Robust Estimation Using the robustlmm Package*

|  | **Perceived Helplessness**  **[Registered Model]** | | | | | **Perceived Helplessness**  **[Robust Model]** | | | | |
| --- | --- | --- | --- | --- | --- | --- | --- | --- | --- | --- |
| *Predictors* | *Estimates* | *std. Beta* | *CI* | *standardized CI* | *p* | *Estimates* | *std. Beta* | *CI* | *standardized CI* | *p* |
| (Intercept) | 4.08 | 0.03 | 3.85 – 4.30 | -0.10 – 0.17 | **<0.001** | 4.10 | 0.05 | 3.85 – 4.35 | -0.11 – 0.21 | **<0.001** |
| Tear Intensity [no tears vs. tears] | 0.87 | 0.54 | 0.63 – 1.10 | 0.39 – 0.68 | **<0.001** | 0.88 | 0.55 | 0.64 – 1.12 | 0.40 – 0.70 | **<0.001** |
| Tear Intensity [subtle vs. intense] | 0.40 | 0.25 | 0.10 – 0.70 | 0.06 – 0.44 | **0.009** | 0.43 | 0.27 | 0.12 – 0.74 | 0.08 – 0.46 | **0.006** |
| **Random Effects** | | | | | | | | | | |
| σ^2^ | 1.81 | | | | | 2.07 | | | | |
| τ_00_ | 0.37 _ID_ | | | | | 0.22 _ID_ | | | | |
|  | 0.14 _PIC_ID:Actor_ID_ | | | | | 0.14 _PIC_ID:Actor_ID_ | | | | |
|  | 0.08 _Actor_ID_ | | | | | 0.11 _Actor_ID_ | | | | |
| ICC | 0.25 | | | | | 0.19 | | | | |
| N | 300 _ID_ | | | | | 300 _ID_ | | | | |
|  | 70 _PIC_ID_ | | | | | 70 _PIC_ID_ | | | | |
|  | 10 _Actor_ID_ | | | | | 10 _Actor_ID_ | | | | |
| Observations | 1500 | | | | | 1500 | | | | |
| Marginal R^2^ / Conditional R^2^ | 0.079 / 0.307 | | | | | 0.078 / 0.250 | | | | |

**Supplementary Table 20**

*Overview of Multilevel Model with Perceived Facial Expression Intensity as the Outcome Variable and Facial Expression Intensity (Natural vs. Exaggerated) as the Predictor. Random Intercepts Based on Participants and Pictures Nested in Actors. Left Side Shows the originally Registered Model and the Right Side Shows the Model with Robust Estimation Using the robustlmm Package*

|  | **Perceived Facial Expression Intensity**  **[Registered Model]** | | | | | **Perceived Facial Expression Intensity**  **[Robust Model]** | | | | |
| --- | --- | --- | --- | --- | --- | --- | --- | --- | --- | --- |
| *Predictors* | *Estimates* | *std. Beta* | *CI* | *standardized CI* | *p* | *Estimates* | *std. Beta* | *CI* | *standardized CI* | *p* |
| (Intercept) | 4.05 | -0.47 | 3.80 – 4.29 | -0.62 – -0.32 | **<0.001** | 4.20 | -0.38 | 3.97 – 4.43 | -0.52 – -0.23 | **<0.001** |
| Facial Expression Intensity (natural vs. exaggerated) | 1.76 | 1.09 | 1.40 – 2.12 | 0.86 – 1.31 | **<0.001** | 1.62 | 1.00 | 1.26 – 1.97 | 0.78 – 1.21 | **<0.001** |
| **Random Effects** | | | | | | | | | | |
| σ^2^ | 1.31 | | | | | 1.56 | | | | |
| τ_00_ | 0.17 _ID_ | | | | | 0.00 _ID_ | | | | |
|  | 0.52 _PIC_ID:Actor_ID_ | | | | | 0.45 _PIC_ID:Actor_ID_ | | | | |
|  | 0.01 _Actor_ID_ | | | | | 0.00 _Actor_ID_ | | | | |
| ICC | 0.35 | | | | |  | | | | |
| N | 300 _ID_ | | | | | 300 _ID_ | | | | |
|  | 70 _PIC_ID_ | | | | | 70 _PIC_ID_ | | | | |
|  | 10 _Actor_ID_ | | | | | 10 _Actor_ID_ | | | | |
| Observations | 1500 | | | | | 1500 | | | | |
| Marginal R^2^ / Conditional R^2^ | 0.275 / 0.526 | | | | | 0.292 / NA | | | | |

**Supplementary Table 21**

*Overview of Multilevel Model with Perceived Overall Expression Intensity as the Outcome Variable and Facial Expression Intensity (Natural vs. Exaggerated) as the Predictor. Random Intercepts Based on Participants and Pictures Nested in Actors. Left Side Shows the originally Registered Model and the Right Side Shows the Model with Robust Estimation Using the robustlmm Package.*

|  | **Perceived Overall Expression Intensity**  **[Registered Model]** | | | | | **Perceived Overall Expression Intensity**  **[Robust Model]** | | | | |
| --- | --- | --- | --- | --- | --- | --- | --- | --- | --- | --- |
| *Predictors* | *Estimates* | *std. Beta* | *CI* | *standardized CI* | *p* | *Estimates* | *std. Beta* | *CI* | *standardized CI* | *p* |
| (Intercept) | 4.05 | -0.26 | 3.80 – 4.30 | -0.41 – -0.10 | **<0.001** | 4.06 | -0.25 | 3.83 – 4.29 | -0.40 – -0.10 | **<0.001** |
| Facial Expression Intensity (natural vs. exaggerated) | 0.89 | 0.56 | 0.57 – 1.20 | 0.36 – 0.76 | **<0.001** | 0.96 | 0.61 | 0.62 – 1.30 | 0.39 – 0.82 | **<0.001** |
| **Random Effects** | | | | | | | | | | |
| σ^2^ | 1.64 | | | | | 1.68 | | | | |
| τ_00_ | 0.36 _ID_ | | | | | 0.27 _ID_ | | | | |
|  | 0.36 _PIC_ID:Actor_ID_ | | | | | 0.41 _PIC_ID:Actor_ID_ | | | | |
|  | 0.03 _Actor_ID_ | | | | | 0.00 _Actor_ID_ | | | | |
| ICC | 0.32 | | | | |  | | | | |
| N | 300 _ID_ | | | | | 300 _ID_ | | | | |
|  | 70 _PIC_ID_ | | | | | 70 _PIC_ID_ | | | | |
|  | 10 _Actor_ID_ | | | | | 10 _Actor_ID_ | | | | |
| Observations | 1500 | | | | | 1500 | | | | |
| Marginal R^2^ / Conditional R^2^ | 0.074 / 0.366 | | | | | 0.118 / NA | | | | |

**Supplementary Figure 15**

*Overview of Ratings for Perceived Tear Intensity (A) and Perceived Facial Expression Intensity (B) for Validation Round 1 and Round 2. Updated Pictures Are Depicted with Thick Vertical Lines. Horizontal Lines Depict Group Means, Dashed Lines Means for Round 1 (Study 3a), and Solid Lines Means for Round 2 (Study 3b)*


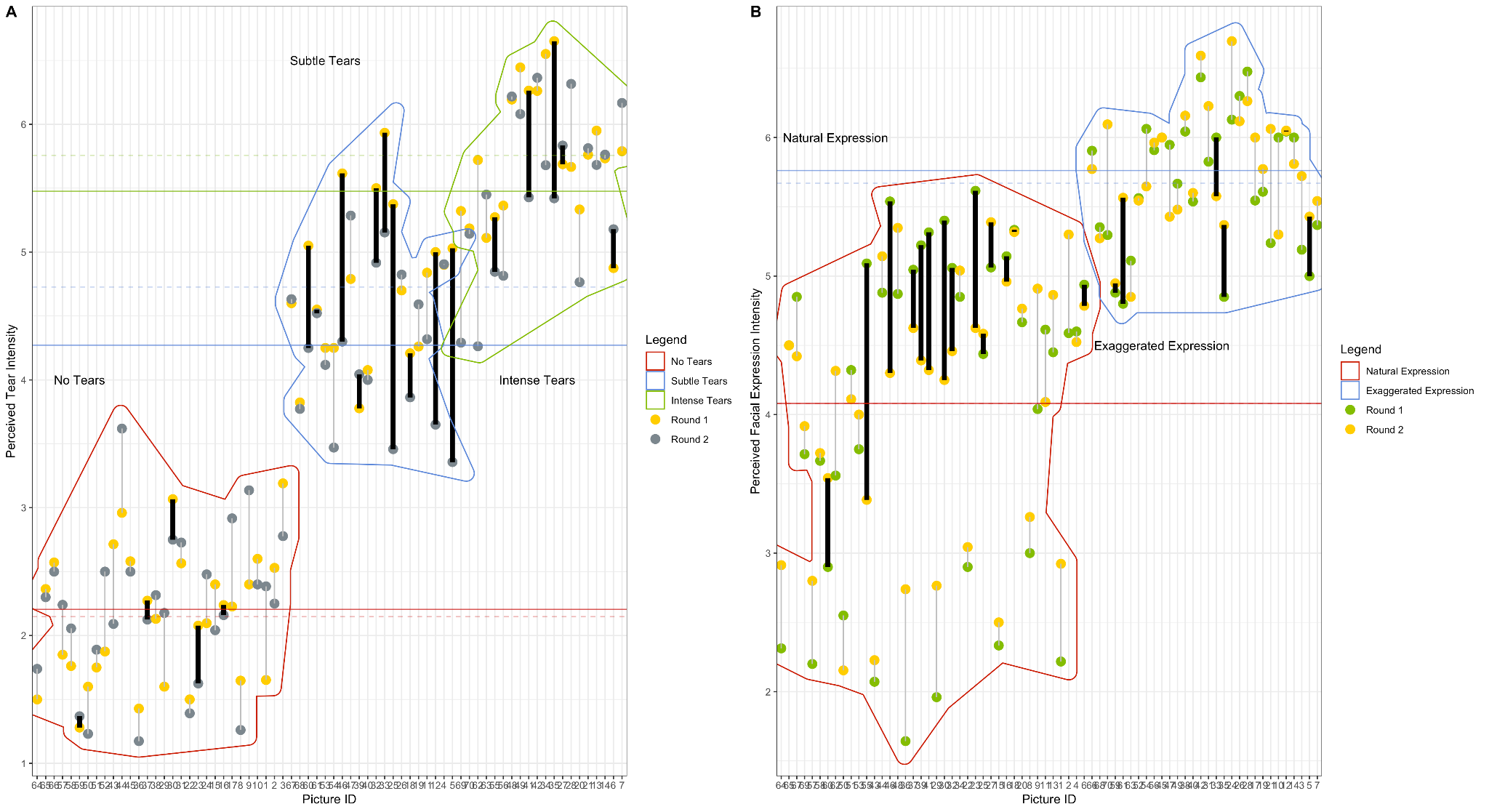


**6.2. Inter-Rater Reliabilities**

We explored inter-rater reliabilities for the pictures as in Study 1.

First, we computed two multilevel intercept-only models with perceived tear intensity and perceived facial expression intensity as outcomes. Participants were added as random effects, as well as pictures nested in actors. We observed a singular fit for these models and changed the random effect for pictures nested in actors to one with a global picture id (from 1 to 70). For perceived tear intensity we observed high consistency in how stimuli were perceived (ICC = 0.54) and less variance explained by differences in participants (ICC = 0.09). Similarly, we observed the same pattern for perceived facial expression intensity (stimuli ICC = 0.47; participant ICC = 0.06).

Second, we computed intraclass correlation coefficients and Gwet’s agreement coefficient for each separate picture based on ratings on perceived tear intensity and perceived facial expression intensity. An overview is provided in Supplementary Table 22. Most inter-rater reliabilities were acceptable though we observed 13 out of 70 pictures (18.6%) that showed low to poor inter-rater reliability (see red rows in Supplementary Table 22). We set the threshold for low inter-rater reliability at an ICC lower than 0.40 and an AC2 lower than 0.40. We set this to 0.40 due to only two measures for the pictures that were also based on intensity for which we expect more variation than for presence/absence measures used in Study 1. When using the 0.50 threshold 22 pictures showed low to poor reliability. Notably, five out of 10 actors showed poor inter-rater reliability for picture ID 0 being the sad picture with a natural facial expression and no tears. We recommend authors to consult individual inter-rater reliabilities if they only want to use single stimuli instead of the whole dataset.

**Supplementary Table 22**

*Overview of Inter-Rater Reliabilities for the Pictures in Study 3b Including Intra-Class Correlation Coefficient (ICC) and Gwet’s Agreement Coefficient (AC2). Pictures with Low Reliabilities on Either Measure are Highlighted in Red*

| Actor_ID | Picture_ID | ICC | | | | Gwet’s AC_2_ | | | | n_raters |
| --- | --- | --- | --- | --- | --- | --- | --- | --- | --- | --- |
|  |  | Est. | | 95% CI | | Est. | | 95% CI | |  |
| 1 | 0 | 0.00 | -0.07 | | 0.99 | 0.07 | -0.58 | | 0.72 | 13 |
| 1 | 1 | 0.65 | 0.23 | | 1.00 | 0.41 | 0.11 | | 0.70 | 20 |
| 1 | 2 | 0.68 | 0.26 | | 1.00 | 0.54 | -3.25 | | 1.00 | 18 |
| 1 | 3 | 0.01 | -0.04 | | 0.98 | 0.63 | -0.33 | | 1.00 | 21 |
| 1 | 4 | 0.42 | 0.10 | | 1.00 | 0.31 | -4.70 | | 1.00 | 28 |
| 1 | 5 | 0.00 | -0.03 | | 0.98 | 0.59 | -0.08 | | 1.00 | 28 |
| 1 | 6 | 0.12 | -0.01 | | 0.99 | 0.53 | -1.43 | | 1.00 | 24 |
| 2 | 0 | 0.59 | 0.19 | | 1.00 | 0.68 | -3.83 | | 1.00 | 23 |
| 2 | 1 | 0.36 | 0.06 | | 1.00 | 0.37 | -0.38 | | 1.00 | 22 |
| 2 | 2 | 0.60 | 0.19 | | 1.00 | 0.33 | -0.19 | | 0.84 | 20 |
| 2 | 3 | -0.02 | -0.04 | | 0.96 | 0.75 | 0.55 | | 0.95 | 22 |
| 2 | 4 | 0.52 | 0.14 | | 1.00 | 0.40 | -4.54 | | 1.00 | 20 |
| 2 | 5 | 0.34 | 0.05 | | 1.00 | 0.55 | -0.66 | | 1.00 | 22 |
| 2 | 6 | -0.05 | -0.05 | | 0.48 | 0.10 | -0.42 | | 0.63 | 21 |
| 3 | 0 | 0.02 | -0.03 | | 0.98 | 0.30 | -2.09 | | 1.00 | 24 |
| 3 | 1 | 0.71 | 0.30 | | 1.00 | 0.61 | -0.64 | | 1.00 | 25 |
| 3 | 2 | 0.69 | 0.28 | | 1.00 | 0.47 | -3.76 | | 1.00 | 24 |
| 3 | 3 | 0.24 | 0.02 | | 1.00 | 0.36 | -1.44 | | 1.00 | 22 |
| 3 | 4 | 0.19 | 0.01 | | 1.00 | 0.41 | -3.04 | | 1.00 | 22 |
| 3 | 5 | -0.06 | -0.06 | | -0.06 | 0.53 | -0.51 | | 1.00 | 17 |
| 3 | 6 | -0.04 | -0.06 | | 0.96 | 0.49 | -0.97 | | 1.00 | 16 |
| 4 | 0 | 0.58 | 0.18 | | 1.00 | 0.60 | -3.04 | | 1.00 | 23 |
| 4 | 1 | 0.76 | 0.36 | | 1.00 | 0.68 | -1.70 | | 1.00 | 24 |
| 4 | 2 | 0.79 | 0.39 | | 1.00 | 0.60 | -5.23 | | 1.00 | 23 |
| 4 | 3 | 0.27 | 0.03 | | 1.00 | 0.69 | 0.44 | | 0.95 | 24 |
| 4 | 4 | 0.38 | 0.06 | | 1.00 | 0.38 | -2.26 | | 1.00 | 17 |
| 4 | 5 | 0.03 | -0.04 | | 0.99 | 0.11 | -2.31 | | 1.00 | 18 |
| 4 | 6 | -0.05 | -0.06 | | 0.62 | 0.52 | -1.60 | | 1.00 | 19 |
| 5 | 0 | 0.07 | -0.04 | | 0.99 | -0.03 | -1.05 | | 0.99 | 17 |
| 5 | 1 | 0.23 | 0.00 | | 1.00 | 0.33 | -2.33 | | 1.00 | 16 |
| 5 | 2 | 0.70 | 0.29 | | 1.00 | 0.44 | -5.09 | | 1.00 | 22 |
| 5 | 3 | 0.05 | -0.02 | | 0.99 | 0.70 | 0.32 | | 1.00 | 24 |
| 5 | 4 | 0.02 | -0.03 | | 0.98 | 0.60 | -1.69 | | 1.00 | 26 |
| 5 | 5 | 0.08 | -0.02 | | 0.99 | 0.50 | 0.49 | | 0.51 | 25 |
| 5 | 6 | -0.05 | -0.06 | | 0.46 | 0.58 | -0.22 | | 1.00 | 19 |
| 6 | 0 | 0.51 | 0.14 | | 1.00 | 0.66 | -4.37 | | 1.00 | 23 |
| 6 | 1 | 0.58 | 0.10 | | 1.00 | 0.54 | -0.81 | | 1.00 | 8 |
| 6 | 2 | 0.73 | 0.32 | | 1.00 | 0.47 | -2.01 | | 1.00 | 19 |
| 6 | 3 | 0.00 | -0.04 | | 0.98 | 0.59 | -0.57 | | 1.00 | 23 |
| 6 | 4 | 0.38 | 0.08 | | 1.00 | 0.44 | -0.47 | | 1.00 | 30 |
| 6 | 5 | 0.24 | 0.03 | | 1.00 | 0.63 | -0.54 | | 1.00 | 28 |
| 6 | 6 | 0.01 | -0.04 | | 0.98 | 0.73 | -0.96 | | 1.00 | 22 |
| 7 | 0 | -0.04 | -0.05 | | 0.88 | 0.43 | -0.34 | | 1.00 | 22 |
| 7 | 1 | 0.27 | 0.03 | | 1.00 | 0.36 | -5.39 | | 1.00 | 21 |
| 7 | 2 | 0.75 | 0.33 | | 1.00 | 0.50 | -2.11 | | 1.00 | 18 |
| 7 | 3 | -0.05 | -0.05 | | -0.05 | 0.59 | 0.21 | | 0.97 | 20 |
| 7 | 4 | -0.04 | -0.05 | | 0.86 | 0.21 | -1.24 | | 1.00 | 21 |
| 7 | 5 | 0.16 | 0.00 | | 1.00 | 0.62 | -0.87 | | 1.00 | 23 |
| 7 | 6 | 0.07 | -0.02 | | 0.99 | 0.58 | -0.15 | | 1.00 | 25 |
| 8 | 0 | 0.28 | 0.00 | | 1.00 | 0.71 | -3.21 | | 1.00 | 13 |
| 8 | 1 | 0.55 | 0.17 | | 1.00 | 0.58 | -2.26 | | 1.00 | 27 |
| 8 | 2 | 0.67 | 0.26 | | 1.00 | 0.48 | -1.76 | | 1.00 | 22 |
| 8 | 3 | -0.05 | -0.06 | | 0.87 | 0.57 | -0.74 | | 1.00 | 17 |
| 8 | 4 | 0.56 | 0.16 | | 1.00 | 0.57 | 0.09 | | 1.00 | 17 |
| 8 | 5 | 0.30 | 0.05 | | 1.00 | 0.49 | -0.07 | | 1.00 | 26 |
| 8 | 6 | 0.22 | 0.02 | | 1.00 | 0.52 | -2.75 | | 1.00 | 27 |
| 9 | 0 | 0.05 | -0.02 | | 0.99 | 0.36 | 0.04 | | 0.68 | 25 |
| 9 | 1 | 0.44 | 0.09 | | 1.00 | 0.57 | 0.50 | | 0.64 | 18 |
| 9 | 2 | 0.74 | 0.32 | | 1.00 | 0.61 | -4.39 | | 1.00 | 19 |
| 9 | 3 | 0.17 | 0.00 | | 1.00 | 0.72 | 0.45 | | 0.99 | 24 |
| 9 | 4 | 0.27 | 0.03 | | 1.00 | 0.61 | 0.59 | | 0.63 | 23 |
| 9 | 5 | -0.05 | -0.06 | | 0.43 | 0.48 | -0.63 | | 1.00 | 19 |
| 9 | 6 | 0.12 | -0.02 | | 0.99 | 0.57 | -0.33 | | 1.00 | 20 |
| 10 | 0 | 0.28 | 0.04 | | 1.00 | 0.37 | -1.14 | | 1.00 | 23 |
| 10 | 1 | 0.61 | 0.20 | | 1.00 | 0.64 | 0.42 | | 0.87 | 20 |
| 10 | 2 | 0.68 | 0.26 | | 1.00 | 0.40 | -1.96 | | 1.00 | 22 |
| 10 | 3 | -0.03 | -0.05 | | 0.96 | 0.72 | 0.05 | | 1.00 | 19 |
| 10 | 4 | 0.29 | 0.04 | | 1.00 | 0.46 | -1.54 | | 1.00 | 22 |
| 10 | 5 | -0.01 | -0.04 | | 0.97 | 0.51 | -0.68 | | 1.00 | 24 |
| 10 | 6 | 0.16 | 0.00 | | 1.00 | 0.63 | -2.08 | | 1.00 | 21 |
